# deMEM: a novel divide-and-conquer framework based on de Bruijn graph for scalable multiple sequence alignment

**DOI:** 10.1093/gigascience/giaf163

**Published:** 2026-01-05

**Authors:** Yanming Wei, Zhaoyang Huang, Pinglu Zhang, Yizheng Wang, Yan Li, Liang Yu, Quan Zou

**Affiliations:** School of Computer Science and Technology, Xidian University, No. 266, Xinglong Section of Xifeng Road, Chang’an Zone, Xi’an, 710126, China; Institute of Digital Health, Yangtze Delta Region Institute (Quzhou), University of Electronic Science and Technology of China, University of Electronic Science and Technology of China, No. 1, Chengdian Road, Kecheng Zone, Quzhou, 324003, China; School of Computer Science and Technology, Xidian University, No. 266, Xinglong Section of Xifeng Road, Chang’an Zone, Xi’an, 710126, China; Institute of Digital Health, Yangtze Delta Region Institute (Quzhou), University of Electronic Science and Technology of China, University of Electronic Science and Technology of China, No. 1, Chengdian Road, Kecheng Zone, Quzhou, 324003, China; Institute of Fundamental and Frontier Sciences, University of Electronic Science and Technology of China, No. 2006, Xiyuan Avenue, Pidu Zone, Chengdu, 610054, China; Institute of Digital Health, Yangtze Delta Region Institute (Quzhou), University of Electronic Science and Technology of China, University of Electronic Science and Technology of China, No. 1, Chengdian Road, Kecheng Zone, Quzhou, 324003, China; Institute of Fundamental and Frontier Sciences, University of Electronic Science and Technology of China, No. 2006, Xiyuan Avenue, Pidu Zone, Chengdu, 610054, China; School of Management, Xi’an Polytechnic University, No.19, Jinhua South Road, Xi’an, 710048, China; School of Computer Science and Technology, Xidian University, No. 266, Xinglong Section of Xifeng Road, Chang’an Zone, Xi’an, 710126, China; Institute of Digital Health, Yangtze Delta Region Institute (Quzhou), University of Electronic Science and Technology of China, University of Electronic Science and Technology of China, No. 1, Chengdian Road, Kecheng Zone, Quzhou, 324003, China; Institute of Fundamental and Frontier Sciences, University of Electronic Science and Technology of China, No. 2006, Xiyuan Avenue, Pidu Zone, Chengdu, 610054, China

**Keywords:** multiple sequence alignment, maximum exact match, de Bruijn graph, parallel algorithm design

## Abstract

**Background:**

Multiple sequence alignment (MSA) continues to be a central challenge in comparative genomics, where the quality of alignment plays a crucial role in determining the accuracy of downstream analyses. However, the challenge of large-scale alignment remains significant.

**Findings:**

This article introduces deMEM, a novel and effective framework for DNA multiple sequence alignment, which enables existing MSA methods such as MAFFT to handle extremely large sequences. deMEM is a 3-stage alignment process: (i) representing maximum exact matches using a de Bruijn graph and clustering them based on their area, (ii) employing a novel divide-and-conquer framework for alignment, and (iii) providing profile–profile alignment between different clusters.

**Conclusions:**

DeMEM enables existing methods like MAFFT to align an extremely large number of sequences, including long sequences that cannot be directly aligned, such as those in a dataset of a thousand monkeypox virus genomes. The deMEM package is free and available at https://github.com/malabz/deMEM.

## Introduction

Multiple sequence alignment (MSA) is a fundamental problem in bioinformatics. The quality of sequence alignment significantly impacts biological sequence analysis, especially that in next-generation sequencing [1, 2]. MSA results are widely used in various applications, including *de novo* genome assembly [3, 4], detection of single-cell genomes based on sequence alignment [5], and taxonomic assignment of newly sequenced data [6, 7].

In the past few decades, researchers have shown an increased interest in developing efficient MSA methods to enhance alignment accuracy. The guide tree for aligning MSA is a heuristic approach that aligns sequences based on a prebuilt guide tree [8]. The guide tree can be categorized into 2 types: the center star guide tree and the distance estimation tree, with the latter serving as the basis for progressive alignment. The center star guide tree strategy tree has been utilized in HAlign series [9–12], while the progressive alignment method is employed in several tools, such as Clustal [13], MAFFT [14], MUSCLE 3 [15], and FAMSA [16]. WMSA [17] combined center star tree and distance-based guide tree for alignment. The center star guide tree can align a large number of sequences with relatively low alignment quality. In contrast, the progressive alignment method generally produces slightly better-quality alignments, although it is still limited by the quality of the guide tree. To improve alignment quality, researchers have developed postprocessing methods, such as ReformAlign [18], TPMA [19], and ReAlign-N [20].

To address the challenge of large-scale, high-quality MSA, researchers have developed a seed-and-extension strategy and a graph-based strategy [2, 21–23]. The seed-and-extension strategy alignment reduces the MSA problem by focusing on aligning and extending seed regions. Minimap2 [24] employed the seed-and-extension strategy for pairwise sequence alignment. FAME [25] designed a state-of-art model for aligning long sequences through 3 steps: identifying common seeds based on the determined seed patterns, creating chains from seeds, and generating splitting alignments by chains. The FMAlign series [26, 27], inspired by FAME, generated the multiple sequences chain by maximum exact matches (MEMs) based on the FM-index. FMAlign2 further generated MEM based on LCP extension and supported the sequence search with MEMs. Graph-based alignment methods provide another approach to solve MSA. EulerAlign [28, 29] proposed MSA by generating and aligning sequences with a consensus sequence determined by the de Bruijn graph [30], and POA [31] proposed another graph representation to express and generate MSA. abPOA [32] significantly enhances computational efficiency through adaptive-band dynamic programming and SIMD parallelization. deBGA [33] utilizes aligning sequence reads based on the de Bruijn graph. MEMs are fundamental for constructing the de Bruijn graph in MSA. SplitMEM [34] is an efficient method for generating the de Bruijn graph for multiple sequences or genomes. Baier et al. [35] enhanced SplitMEM by the Burrows–Wheeler transform (BWT) to generate MEMs, which significantly reduced the time complexity of the process to O(|*Σ*|), where *Σ* is the length of all sequences.

Although there are many MSA methods, most have suffered from various methodological limitations. First, guide-tree based methods generally been restricted to the quality of the guide tree and the principle of “once a gap, always a gap,” whereby any gap inserted during progressive profile–profile alignment remains fixed and cannot be corrected or refined in subsequent steps. Second, due to the nature of MEMs, seed-and-extension strategy-based methods focus on high-similarity sequence alignment, with little attention to low-similarity sequence alignment. Third, POA is used for generating consensus sequences, particularly in third-generation sequencing, but is neither considered nor discussed by the subalignment methods in the seed-and-extension strategy, which limits the application of POA. Lastly, the research for the de Bruijn graph has tended to focus on third-generation long-read analysis rather than MSA.

To address these challenges, we developed deMEM, an efficient and accurate multiple sequence alignment method based on the divide-and-conquer strategy. It works by (i) splitting the sequences into clusters, using an enhanced version of SplitMEM; (ii) aligning clusters into profiles by the MEMs; and (iii) merging profiles by profile–profile alignment. Our method enhances alignment quality and demonstrates superior performance compared to traditional, seed-and-extension–free MSA strategies such as MAFFT [14], WMSA [17], and abPOA [32] on low-similarity datasets. Additionally, it supports the alignment of extremely long sequences that these seed-and-extension–free methods cannot handle. Furthermore, deMEM outperforms sequence division methods such as FMAlign2 [27] and FAME [25] in handling challenging alignment tasks, such as aligning extremely long sequences.

## Findings

### The framework of deMEM

**Figure 1:**
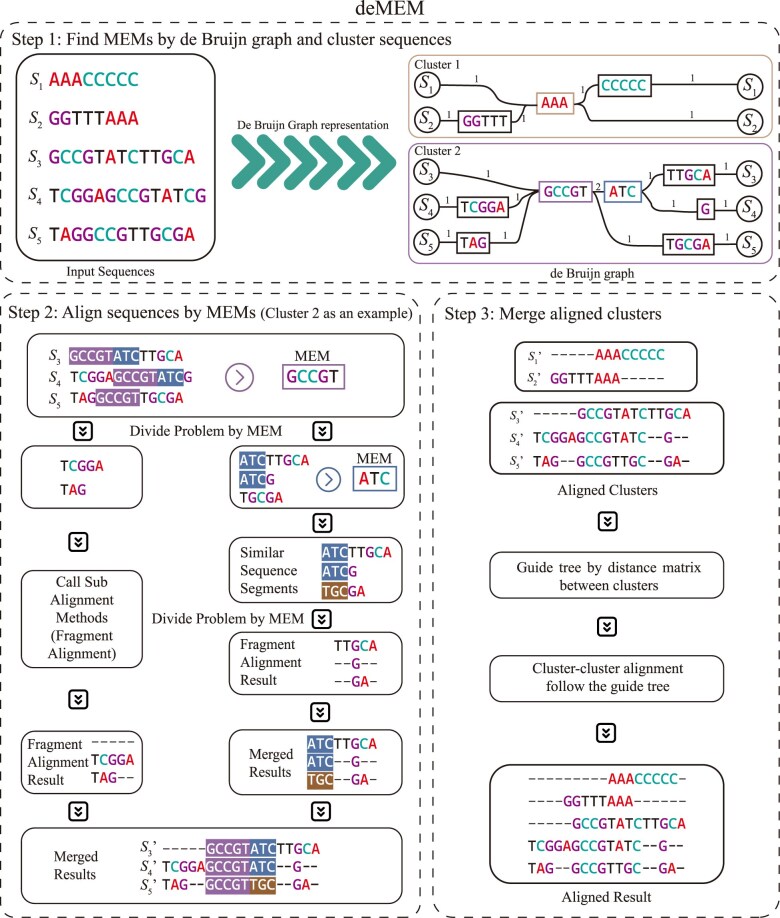
The framework of deMEM. (a) Step 1: Find MEMs by the de Bruijn graph and generate clusters: sequences are read and converted into clusters using SplitMEM [34], which represents the sequences as the de Bruijn graph. The number on the edge in the de Bruijn graph represents the edge weight. (b) Step 2: Align sequences by MEMs: For each cluster, sequences are aligned by a divide-and-conquer framework to process the MEMs. For every MEM, the alignment problem is divided into 3 subproblems: the left subproblem, the right subproblem, and the down subproblem. For sequences not covered by any MEM, the SSW library [36] is used to identify similar fragments. If no similar fragment is found, the sequence is processed in the down subproblem. For subproblems without MEMs, external MSA methods such as MAFFT [14], WMSA [17], or abPOA [32] are employed for alignment. The resulting alignments are then merged to form an aligned cluster. (c) Step 3: Merge aligned clusters to generate MSA: aligned clusters are combined using an external profile–profile merge strategy, such as MAFFT profile merge, WMSA, or abPOA, to generate the final MSA result.

Our developed framework is named deMEM. The architecture of deMEM can be described as follows (Fig. 1):

- Step 1: Input the sequence file *S*, convert sequences to the *k*-mer de Bruijn graph representation with determined threshold *k*, find MEMs, and cluster sequences based on MEMs.
- Step 2: We generated *n* clusters in step 1. For each cluster ${{C}_i},\ i = 1,2,\cdots,n$, we align the cluster by our divide-and-conquer framework based on MEMs obtained from step 1. All clusters are aligned into profiles ${{P}_i},\ i = 1,2,\cdots,n$.
- Step 3: Align *n* clusters by a determined method, such as MAFFT profile merge [14], WMSA [17], or abPOA [32].

### Sequence clustering by the de Bruijn graph

We utilize a clustering algorithm based on the BWT-enhanced SplitMEM [34] algorithm [35]. For graph construction, we employ a disjoint-set union data structure [37] to represent the cluster affiliation of each sequence. In particular, we modify Algorithm 2 in the BWT-enhanced SplitMEM [35] to calculate the cluster. The pseudo-code of modified algorithm is shown in Algorithm S1.

As shown in Algorithm S1, this algorithm uses the disjoint-set data structure to measure the affiliation of sequences. When identical MEMs are found in different sequences, we merge the 2 disjoint-set unions that represent these sequences. Once the sequence affiliations are established, we apply the original strategy from SplitMEM, which uses Depth First Search (DFS) to traverse the de Bruijn graph and generate MEMs for each cluster. After generating the MEMs for the clusters, we leverage this information to perform MSA for each cluster using the divide-and-conquer framework.

In conclusion, the time complexity of sequence clustering is ${\mathrm{O}}( {n( {\log \sigma + \alpha ( {n,\ n} )} )} )$, where $\sigma $ is the size of the alphabet ($\sigma = 4$ in DNA sequences), and $\alpha ( {n,n} )$ is the inverse Ackermann function.

### Divide-and-conquer framework for MSA using MEMs

In this section, we describe the divide-and-conquer framework, where the inputs are MEMs provided by Algorithm S1. The core of deMEM is the divide-and-conquer framework, where MEMs are used to split the alignment problem into smaller subproblems. For each cluster, we sort the MEMs by area, with the largest MEM being the first in the array. Once sorting is complete, the sorted MEMs with the sequences are assigned to a cluster, which is then fed into the divide-and-conquer framework to generate an MSA. We start by defining MEM and its area in multiple strings, then define MEM with similar fragments and their area, to support the representation of sequences that lack MEMs but share similarity with them. After defining MEM with similar fragments, we proceed to introduce the alignment process within the divide-and-conquer framework, using the sorted MEMs to produce the final alignment results for the sequences corresponding to the clusters.

#### The definition of MEM

In this section, we discuss the definition of MEM in detail. Considering the nature of MEMs, when different sequences share similar strings, our method treats these similar strings as fragments and merges them into the MEM. We first provide a formal definition of MEM, followed by the introduction of the concept of MEM with similar fragments, which allows us to represent these MEMs during the sequence alignment process.

The definition of MEM in 2 strings is an exact match between 2 strings that cannot be extended in either direction toward the beginning or end of the 2 strings without allowing for a mismatch [38]. Since our problem involves multiple sequences, we need to extend the definition of MEM to accommodate multiple sequences. Definition 1 provides the definition of MEM in multiple strings:

**Definition 1**: MEM ${\boldsymbol{M}}$ in multiple strings ${{s}_1},{{s}_2},\cdots,{{s}_n}$ is an exact match between multiple strings with match length *L* and cannot be extended in either direction toward the beginning or end of multiple strings without allowing for a mismatch, with the intervals $[ {{{x}_1},{{x}_1} + L} ),[ {{{x}_2},{{x}_2} + L} ),\cdots,[ {{{x}_n},{{x}_n} + L} )$ corresponding to strings ${{s}_1},{{s}_2},\cdots,{{s}_n}$. In other words, ${\boldsymbol{M}} = \{ {L,( {1,{{x}_1}} ),\ ( {2,{{x}_2}} ),\cdots,( {n,{{x}_n}} )} \}$, which means the length of MEM is *L*, and the MEM occurs at sequences ${{s}_1},{{s}_2},\cdots,{{s}_n}$, which begins at ${{x}_1},{{x}_2},\cdots,{{x}_n}$. The number of strings contained in ${\boldsymbol{M}}$ is $| {\boldsymbol{M}} | = n$. If MEM ${\boldsymbol{M}}$ only occurs at sequence ${{s}_{I{{D}_1}}},{{s}_{I{{D}_2}}},\cdots,{{s}_{I{{D}_d}}}$, which occurs at the intervals $[ {{{x}_{I{{D}_1}}},{{x}_{I{{D}_1}}} + L} ),[ {{{x}_{I{{D}_2}}},{{x}_{I{{D}_2}}} + L} ),\cdots,[ {{{x}_{I{{D}_d}}},{{x}_{I{{D}_d}}} + L} )$, we define the MEM as ${\boldsymbol{M}} = \{ {L,( {{{s}_{I{{D}_1}}},{{x}_{I{{D}_1}}}} ),\ ( {{{s}_{I{{D}_2}}},{{x}_{I{{D}_2}}}} ),\cdots,( {{{s}_{I{{D}_d}}},{{x}_{I{{D}_d}}}} )} \}$, and the length of ${\boldsymbol{M}}$ is $| {\boldsymbol{M}} | = d$.

As shown in Definition 1, MEMs in multiple strings can be common seeds in measuring the similarity of all sequences, but the distances in exact matches must be measured in sequences. As a result, we need to define the area of MEM for measuring the importance of every MEM. The definition of the MEM area in multiple sequences is shown in Definition 2.

**Figure 2:**
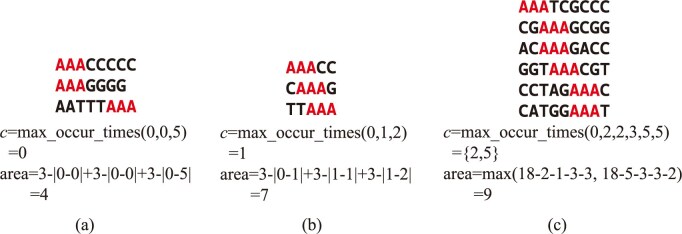
Examples for MEM and MEM area calculation. (a) The definition of MEM is ${{{\boldsymbol{M}}}_1} = \{ {3,( {0,0} ),{\boldsymbol{\ }}( {1,0} ),( {2,5} )} \}$. The center of ${{{\boldsymbol{M}}}_1}$ is 0, because 0 occurs twice and 5 occurs only once. The area of MEM is calculated by formula (1). (b) The definition of MEM is ${{{\boldsymbol{M}}}_2} = \{ {3,( {0,0} ),{\boldsymbol{\ }}( {1,1} ),( {2,2} )} \}$. The center of ${{{\boldsymbol{M}}}_2}$ is 1; because 0, 1, and 2 occur once, we choose the medium number 1 to represent the center. (c) The definition of MEM is ${{{\boldsymbol{M}}}_3} = \{ {3,( {0,0} ),{\boldsymbol{\ }}( {1,2} ),( {2,2} ),( {3,3} ),( {4,5} ),( {5,5} )} \}$. The center of ${{{\boldsymbol{M}}}_3}$ can be 2 or 5, because both 2 and 5 occur twice in ${{{\boldsymbol{M}}}_3}$. Because 2 and 5 both are medium numbers, we need to calculate area to determine the center. If we choose 2 to be the center of ${{{\boldsymbol{M}}}_3}$, the area is 9; in other words, by choosing 5 to be the center of ${{{\boldsymbol{M}}}_3}$, we can calculate the area of ${{{\boldsymbol{M}}}_3}$ as 5. As a result, we choose 2 to be center of ${{{\boldsymbol{M}}}_3}$.

**Definition 2**: The area *a* of MEM ${\boldsymbol{M}}$ is defined as formula (1):


(1) \begin{eqnarray*}
a = \mathop {\max }\limits_c \left( {\mathop \sum \limits_i^{IDs} \left( {L - \left| {{{x}_i} - c} \right|} \right)} \right)
\end{eqnarray*}

where $IDs$ is the set that contains all sequence identifiers in this MEM, and *c* means the “center” of every sequence in the MEM. It is worth noting that the definition of “center” refers to the position with the highest occurrence frequency in the MEM, which is set as the maximum area to ensure a unique area calculation. In particular, center *c* can be calculated as formula (2):


(2) \begin{eqnarray*}
c = \mathop {\max \_{\mathrm{occur}}\_{\mathrm{times}}}\limits_{i \in IDs} \left( {{{x}_i}} \right)
\end{eqnarray*}

where the max_occur_times function calculates the highest occurrence in the list of start positions $\{ {{{x}_i},i \in IDs} \}$. If multiple values have the same maximum frequency, the middle value (i.e., the median among the tied candidates) is selected. If there are two middle values, we compute the corresponding area *a* for each candidate ${{x}_i}$ and choose the one with the maximum area. As shown in formula (2), the meaning of *c* is the “center” of the MEM. If we choose any other $c^{\prime} \neq c$, we cannot determine the area *a* uniquely. The example of area calculation is shown in Fig. 2.

In Definition 1, a MEM is defined as having the same length across sequences. However, for sequences not contained in a given MEM, we identify similar fragments using the SSW library [36], which applies the Smith–Waterman pairwise alignment [39] to measure the similarity between the MEM and the sequence. In our implementation, the Smith–Waterman scoring parameters are set as follows: match = 2, mismatch = −2, gap open penalty = −3, and gap extension penalty = −1. To represent these similar fragments in the alignment process, we extend the concept of MEM and introduce the notion of “MEM with similar fragments.” The formal definition of a MEM with similar fragments is provided in Definition 3, and the corresponding area is given in Definition 4:

**Definition 3**: MEM with similar fragments ${\boldsymbol{MX}}$ in multiple strings ${{s}_1},{{s}_2},\cdots,{{s}_n}$ is an exact match between multiple strings with match length *L* and cannot be extended in either direction toward the beginning or end of multiple strings without allowing for a mismatch. After finding similar parts in MEM, strings ${{s}_{n + 1}},\cdots,{{s}_f}$ had similar parts with ${\boldsymbol{MX}}$, with the interval $[ {{{x}_1},{{x}_1} + L} ),[ {{{x}_2},{{x}_2} + L} ),\cdots,[ {{{x}_n},{{x}_n} + L} ),[ {{{x}_{n + 1}},{{y}_{n + 1}}} ),\cdots,[ {{{x}_f},{{y}_f}} )$ corresponding to string ${{s}_1},{{s}_2},\cdots,{{s}_f}$. In other words, ${\boldsymbol{MX}} = \{ {L,( {I{{D}_1},{{x}_1},0} ),\ \cdots,( {I{{D}_n},{{x}_n},0} ),( {I{{D}_{n + 1}},{{x}_{n + 1}},{{y}_{n + 1}} - {{x}_{n + 1}} - L} ),\cdots,}$${( {I{{D}_f},{{x}_f},{{y}_f} - {{x}_f} - L} )} \}$ which means the length of MEM is *L*, and the MEM occurs at sequences $I{{D}_1},\cdots,I{{D}_n}$ that begin at ${{x}_1},\cdots,{{x}_n}$, which have similar strings in sequences $I{{D}_{n + 1}},\cdots,I{{D}_f}$ that start at ${{x}_{n + 1}},\cdots,{{x}_f}$ with length ${{y}_{n + 1}} - {{x}_{n + 1}},\cdots,{{y}_f} - {{x}_f}$. The number of strings contained in ${\boldsymbol{MX}}$ is $| {{\boldsymbol{MX}}} | = f$.

**Definition 4**: The area *a* of MEM with similar fragment ${\boldsymbol{MX}}$ is defined in formula (3):


(3) \begin{eqnarray*}
a = \mathop {\max }\limits_c \left( {\mathop \sum \limits_i^{IDs} \left( {{{y}_i} - {{x}_i} - \left| {{{x}_i} - c} \right|} \right)} \right)
\end{eqnarray*}

where $IDs$ means sequence identifiers in this MEM, $IDs$ is the set containing all sequence IDs in this MEM, and *c* is the center of every sequence in MEM. The calculation of center *c* is same as in formula (2).

#### Align by sorted MEMs

**Figure 3:**
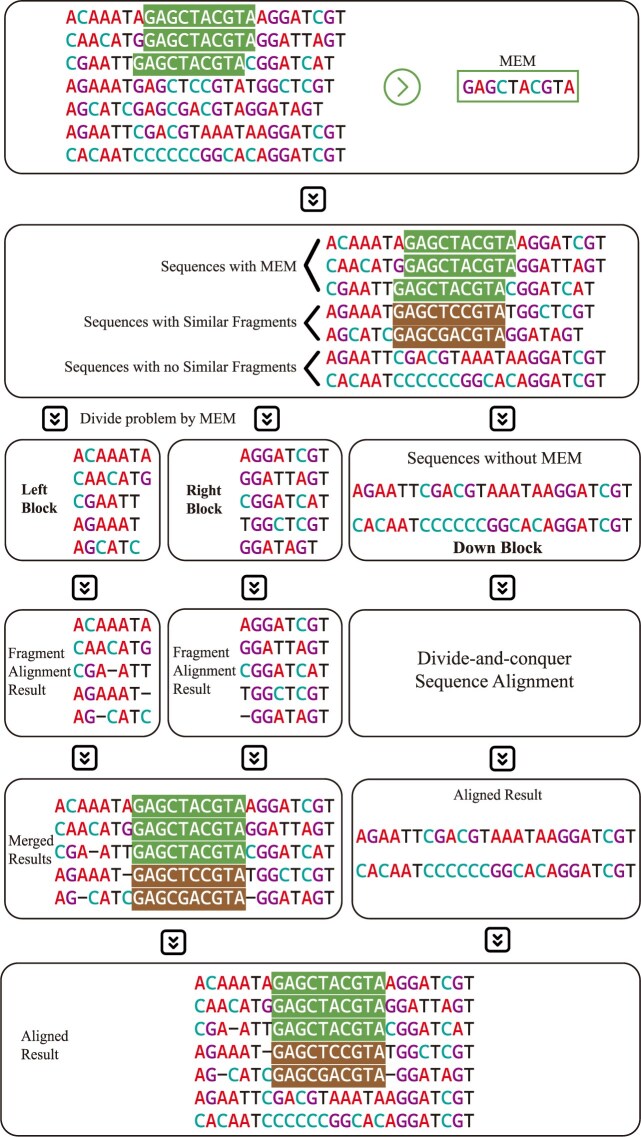
Details for aligning sequences by sorted MEMs. We found MEM “GAGCTACGTA” in these sequences, and the MEM occurs in the first, second, and third sequences (colored area). After that, we called the SSW function and found the similar sequences: “GAGCTCCGTA” and “GAGCGACTA”. As a conclusion, the MEM with a similar fragment is on the first to fifth strings. After generating the MEM with a similar fragment, the left and right blocks are generated, and the down block is also generated. After finding a similar sequence segment, we divide the sequences into 4 parts: left block, right block, down block, and MEM with a similar fragment. The left, right, and down blocks are aligned by the subalignment function (BlockAlign function in Algorithm S2). We need to wait for the left and right block alignment calculation process to generate the sequence alignment result for the sequences in MEM with similar fragments. After generating the sequence result, we need to wait for the block to make an alignment with sequences in MEM and sequences out MEM to generate the final result.

In this section, we use sorted MEMs to generate alignment. It is worth noting that MEMs are sorted by area, with the MEM having the largest area placed first in the array. For every MEM, we follow its guidance to divide the corresponding sequences into 3 parts: left block, right block, and down block (details shown in Fig. 3). For sequences not included in the MEM, we use the SSW library [36] to find similar fragments and incorporate these fragments into the MEM, resulting in a new MEM referred to as “MEM with similar fragments.” Sequences in the “MEM with similar fragments” are divided into left and right blocks, while sequences not included in the “MEM with similar fragments” are placed in the down block. The core of the divide-and-conquer framework for aligning sequences using sorted MEMs is shown in Algorithm S2, and the illustration of Algorithm S2 is shown in Fig. 3.

As shown in Algorithm S2, the alignment procedure can be summarized as follows: we sort MEM blocks according to their area, then call the main divide-and-conquer function for alignment based on MEM blocks. The internal logic of the divide-and-conquer function can be concluded as follows: first, the MEM may not contain all input sequences, and therefore, we use the SSW algorithm to identify the sequences not covered by the MEM. After identifying these sequences, we merge the MEM with the corresponding intervals found by the SSW. This combined block is treated as the MEM block with fragment parts. Using the MEM block with fragment parts, we divide the remaining sequences into 3 subproblems: align the left part, the right part, and the down part. For each MEM, we determine the corresponding appearance in each sequence. The divide-and-conquer function is recursively called to align the subproblems. It is important to note that, if the down part is present, we must align the sequences in the down part with those in the MEM that contain the fragments, each of which has been aligned previously. To accelerate the divide-and-conquer procedure, we adopt a parallelization strategy similar to that used in FORAlign [40], using the fork-join model with work stealing strategy to enhance computational efficiency.

### Profile–profile alignment and fragment alignment based on existing approaches

In previous sections, we introduced the alignment based on MEMs. In Figure 1, step 2, after processing MEM, we call the subalignment methods to make alignment. The subalignment method is the same as the father alignment method, except for the condition of no MEMs. If any part has no MEMs, we call the fragment alignment method. We determine the fragment alignment method, as in MAFFT FFT-NS-1 [14], WMSA [17] or abPOA [32]. Like the subalignment, we also compute profiles alignment by these methods. Since these methods cannot make profile–profile alignment directly, we modify them to support profile alignment. In particular, we made a list for representing sequences to profiles and call profile–profile alignment to make the real alignment.

### Time and space analysis for the whole algorithm

deMEM can be divided into 3 modules: (A) identifying MEMs and clustering sequences based on the de Bruijn graph; (B) aligning sequences within each cluster, using the divide-and-conquer framework based on MEMs to generate profiles; and (C) performing profile–profile alignment between different clusters. The time and space complexity analysis of the algorithm is presented as follows:

- Find MEMs and make clusters based on the de Bruijn graph: assume we have *n* sequences, and the length of the sequences is *S*. We use BWT-enhanced SplitMEM to find MEMs. The time complexity of this algorithm is $O( {( {n + S} )\log \sigma } ) \approx O( {n + S} )$, and the space complexity is $O( {n + S} )$ to store all nodes in the de Bruijn graph. To make clusters based on MEMs, we need to use the disjoint set for making clusters, and the time complexity of clustering is $O( {( {n + S} )\alpha ( {n,n} )} ) \approx O( {n + S} )$. In conclusion, the time and space complexity of step (A) is $O( {n + S} )$.
- Divide-and-conquer framework based on MEMs: assume we have *n* sequences with the minimum sequence length *m*, and we can infer that the graph length in step (A) has a maximum of $O( {nm} )$ nodes. For every MEM, calculate the area needs ${\mathrm{O}}( {n\log n} )$ time. The graph has at most $O( {nm} )$ nodes, and the time complexity of the sort is $O( {nm\log ( {nm} )} )$. Next, we align blocks by sorted MEMs. For every MEM, assume that this size of MEM is $A \times y$, and the size of the aligned region is $n \times m$, where *A* is the number of sequences, *y* is the length of the MEM, *n* is the number of sequences, and *m* is the length of all sequences. Time complexity of region alignment is $T( {n,m} ) = T( {A,l} ) + T( {A,r} ) + T( {n - A,m} ) + S( {n,m} )$. We discuss the result of $S( {n,m} )$: first, we try to use SSW to find the similarity. We need to find $n - A$ sequences, and the time complexity of SSW is ${\mathrm{O}}( {( {n - A} )ky} )$ (with K-band) or ${\mathrm{O}}( {( {n - A} )my} )$ (without K-band). Then, we divide other MEMs into 3 parts, which need $O( {nm} )$ time; next, we wait for the results of the subfunction, as well as merge and refine the results, which need $O( A )$. Finally, the overall alignment condition is that there are no MEMs in any part. We refer to this as MAFFT, WMSA, or abPOA for alignment purposes. The time complexity of the entire algorithm may be affected by the chosen method. If we use WMSA with the K-band, the time complexity of alignment is $O( {xyk} )$; otherwise, the time complexity of alignment is $O( {x{{y}^2}} )$. In conclusion, $S( {n,m} ) = O( {knm} )$ (with K-band) ~ $O( {n{{m}^2}} )$ (without K-band). The analysis for $T( {n,m} )$ is similar to [41]. As for a result, $T( {n,m} ) = O( {knm} )$ (with K-band) ~ $O( {n{{m}^2}} )$ (without K-band).
- Profile–profile alignment between different clusters: assume we generate *C* clusters in step (A), the maximum length of profile is *P*. The profile–profile alignment step requires a progressive profile–profile alignment process. Since $C \ll P$, the time complexity of this step is ${\mathrm{O}}( {{{C}^3} + {{C}^2}{{P}^2}} ) \approx {\mathrm{O}}( {{{C}^2}{{P}^2}} )$, and the space complexity is ${\mathrm{O}}( {{{C}^2}{{P}^2}} )$.

As a result, the time and space complexity of the whole deMEM algorithm is ${\mathrm{O}}( {Cn{{m}^2} + {{C}^2}{{P}^2}} )$, where *C* represents the number of clusters, *n* is the number of sequences, *m* is the length of the longest sequence, and *P* is the length of the aligned profiles.

### Datasets and measurement

To evaluate the alignment results of our proposed method, we developed a software package called deMEM. In this section, we first introduce the datasets used to compare deMEM with other methods, followed by a description of the test methods. Finally, we outline the evaluation metrics and computational resources utilized in this experiment.

**Experimental datasets**: Because deMEM divides sequences into multiple parts, multiple conditions are required to demonstrate the advantages of our method. To comprehensively evaluate its performance, we conducted experiments on both real and simulated datasets. Thus, we choose the following datasets, shown in Table 1 and Table 2.

**Table 1:** Description of the datasets tested in deMEM (real data)

Dataset name	Source of dataset	Sequences	Average sequence length	Length distribution	References
mt1x	Mt genomes	672	16,568.3	16,555~16,578	[11, 17, 25, 42]
mt20x		13,440			
Complete156	SARS-CoV-2	156	29,855.1	29,409~29,927	[17, 42]
Mix1t		1,024	27,556.8	64~29,981	
MPoX	Monkey Pox virus	1,739	197,084.9	183,230~210,918	[42, 43]
Variola	Variola virus	4	186,374.3	186,064~186,677	[25]
Mycoplasma	*Mycoplasma bovis*		579,708.8	579,504~579,977	
Streptococcus	*Streptococcus pneumonia*		2,160,522	2,111,882~2,184,682	
Ecoli	*Escherichia coli*		4,633,445.8	4,578,159~4,686,137	
Neisseria	*Neisseria meningitidis*	5	2,190,087.6	2,145,295~2,272,360	First collected
23sr	*Mycobacterium* 23S rRNA	641	3,113.1	1,909~3,485	[11]

**Table 2:** Description of the datasets tested in deMEM (simulated data)

Test Name	Sequences	Average length	Length distribution	Test cases	Reference
RNA-255	255	1,527	1,518~1,542	10	[44]
RNA-511	511	1,528	1,518~1,542		
RNA-1023	1,023	1,527	1,517~1,542		
RNA-2047	2,047	1,527	1,517~1,542		
RNA-4095	4,095	1,527	1,516~1,542		
mt-similarity	112	15,860 ± 115	15,719 ± 220~15,992 ± 12	9	[11]
SARS-CoV-2-similarity	112	29,675 ± 118	29,404 ± 316~30,000 ± 0		

In Table 1, we collected the *Neisseria meningitidis* sequences to show the quality of our methods.

**Experimental methods**: We compared our method with FAME [25] and FMAlign2 [27], both of which use chain-based strategies. As described before, we employed MAFFT FFT-NS-1 [14], abPOA [32], and WMSA [17] to calculate subalignments. Our experiments can be divided into 2 main parts: (i) evaluating the improvements by FAME, FMAlign2, and our method for subalignment strategies, such as MAFFT FFT-NS-1, and (ii) comparing the different subalignment strategies, such as MAFFT FFT-NS-1, abPOA, and WMSA, under 2 approaches: treating all sequences as a cluster (methods *-H in result tables) or grouping sequences into multiple clusters (methods *-L in result tables). It is worth noting that if deMEM does not find any MEMs, the program falls back to the original alignment method. For consistency, since different alignment methods employ distinct scoring systems (e.g., abPOA uses a 2-piece gap affine penalty scoring system, while WMSA and MAFFT use a simple gap affine penalty scoring system), all alignment methods were evaluated using their default parameters.

**Experimental metrics**: We measure the real data alignment results by the SP score introduced in [26], with match score = 0, mismatch score = −1, and gap score = −2. A lower SP score indicates fewer inserted gaps, reflecting improved alignment consistency and overall quality. The quality improvement, denoted as $S{{P}_{imp}}( {{\mathrm{M}}1,\ {\mathrm{M}}2} )$, can be calculated as formula (4):


(4) \begin{eqnarray*}
S{{P}_{imp}}\left( {{\mathrm{M}}1,{\mathrm{\ M}}2} \right) = \frac{{{\mathrm{M}}1 - {\mathrm{M}}2}}{{\left| {{\mathrm{M}}1} \right|}}
\end{eqnarray*}

where ${\mathrm{M}}1$ and ${\mathrm{M}}2$ represent the SP scores generated by 2 different methods. Here, $S{{P}_{imp}}( {{\mathrm{M}}1,\ {\mathrm{M}}2} )$ quantifies the percentage improvement of method ${\mathrm{M}}2$ relative to method ${\mathrm{M}}1$. For simulated datasets, we use the Q and TC score designed in MUSCLE [15] for measuring the results for all methods.

**Computational resources**: Our experiment is tested on a workstation with 1 TB main memory on an Intel Xeon Gold 6230 CPU processor with 80 cores with 2.10 GHz CPU frequency under the Ubuntu 20.04 operating system.

### Experimental results on real datasets

**Figure 4:**
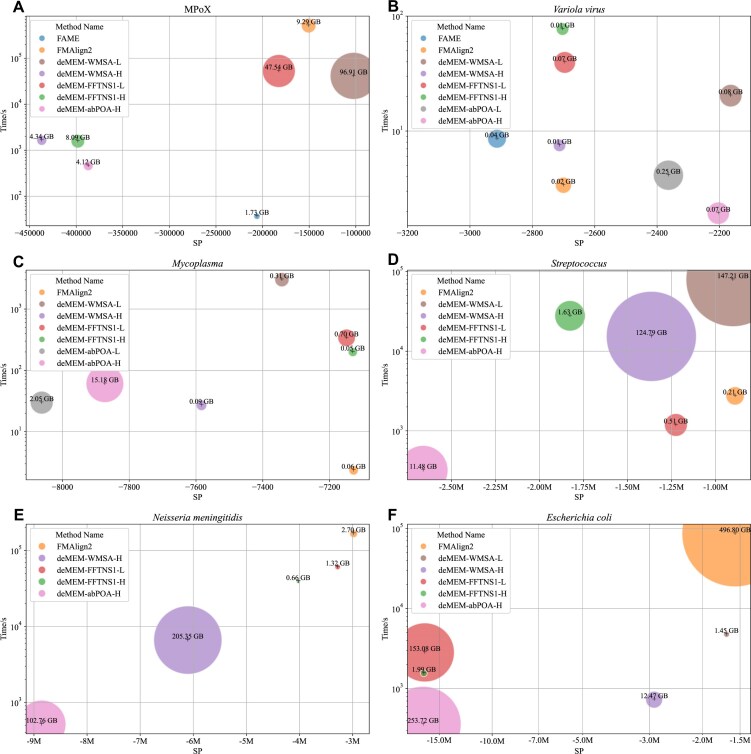
Illustration of real dataset results with different methods. The circled area indicates the memory usage of the determined method. This figure shows the best SP values between the same method, excluding the out-of-memory methods. Lower absolute SP scores indicate better alignment quality.

In this section, we present the results of real datasets. A summary of results is provided in Fig. 4, with detailed results provided in Supplementary Table S1. From these results, we observe that our method improves the SP scores, particularly in extremely large datasets. Compared to seed-and-extension MSA methods, our method achieved higher SP scores than FMAlign2 and FAME. A key advantage of deMEM is its ability to integrate both vertical and horizontal sequence information, leading to more comprehensive sequence alignments. For extremely large and long sequences, such as those in the MPoX dataset, although FAME achieved faster alignment with lower memory consumption, it produced lower-quality results compared to deMEM. Under our experimental metrics, where higher (less negative) SP scores indicate better alignments, deMEM improves SP scores on the MPoX dataset by approximately 50.3% ($S{{P}_{imp}}( {{\mathrm{FAME}},\ {\mathrm{deMEM}}} )$) and 32.0% ($S{{P}_{imp}}( {{\mathrm{FMAlign}}2,\ {\mathrm{deMEM}}} )$) compared with FAME and FMAlign2 (Supplementary Table S1), as calculated using formula (4).

#### Comparison between seed-and-extension MSA methods

To evaluate the enhancement for SP scores between FMAlign2, FAME, and our method, we independently ran the MAFFT FFT-NS-1 method and calculated the SP score using the aforementioned methods. The results are presented in Table 3. Due to the limitations of MAFFT FFT-NS-1, it can only directly align sequences in the cases listed in Table 3. It is worth noting that, compared to FAME and FMAlign2, our method enhanced the performance of FFT-NS-1 when aligning large sequences. Specifically, compared to FAME, deMEM can align extremely long sequences, without any decrease in SP scores, and demonstrates improved robustness, consistently aligning sequences with stability. Compared to FMAlign2, our method performs better on mt sequences. For extremely large and long sequences, such as *Mycoplasma bovis* sequences, deMEM offers modest improvements in alignment quality but uses significantly less memory.

**Table 3:** Result differences between seed-and-extension methods and MAFFT FFT-NS-1. “Block size” in this table means the maximum SP score aligned by the determined MEM block size. ΔSP means the difference value between the determined method with MAFFT FFT-NS-1. Bold text indicates the method (row) with the highest ΔSP score for this dataset (column).

	mt1x		mt20x		*Mycoplasma bovis*	
Method Name	Block size	ΔSP	Block size	ΔSP	Block size	ΔSP
FAME	−	−52.3	−	−52.2	−	−
FMAlign2	20	9.1	500	7.4	500	**21.2**
deMEM-FFTNS1-L	50	6.2	15,000	7.6	10,000	0.0
deMEM-FFTNS1-H	100	**11.6**	100	**11.6**	5,000	18.7

#### Comparison between subalignment methods in deMEM

deMEM supports various subalignment methods, including abPOA, FFT-NS-1, and WMSA. The quality and speed of alignment are influenced by the choice of subalignment method. Each method has its own advantages: abPOA can align smaller sequences quickly with minimal memory usage, but it is less suitable for long and large sequences; FFT-NS-1 is optimal for aligning highly similar sequences; and WMSA fits in aligning large and long sequences with low similarity. As shown in Fig. 4, our method effectively aligns extremely large datasets, demonstrating its scalability.

**Figure 5:**
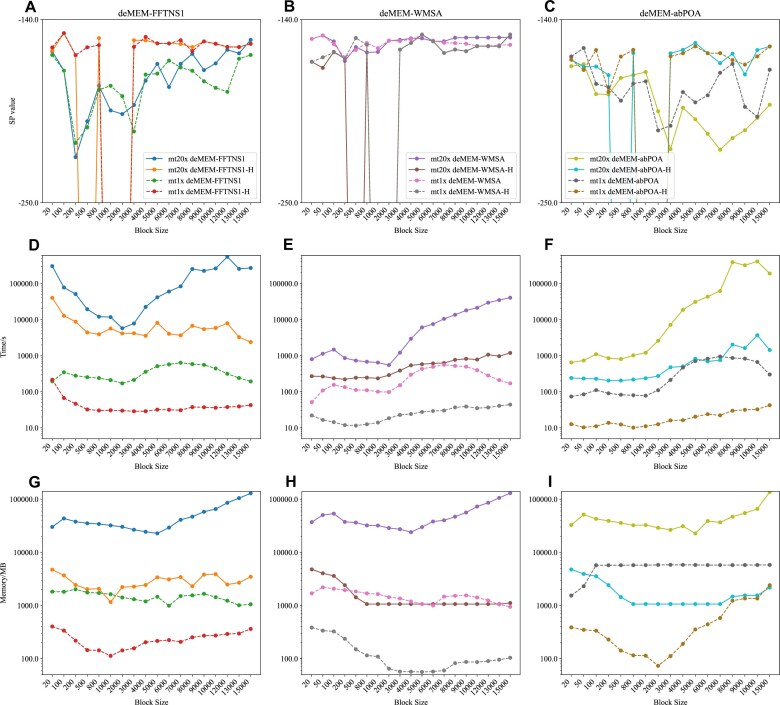
SP scores, time, and memory change with block size in mt1x and mt20x datasets.

To further evaluate the impact between different methods, we tested the mt1x and mt20x datasets using these subalignment methods, as shown in Fig. 5. The results indicate that subalignment methods primarily influence memory and runtime efficiency. Notably, abPOA exhibited slow alignment speeds because it was executed on a single-threaded process, significantly affecting performance.

### Experimental results on simulated datasets

**Figure 6:**
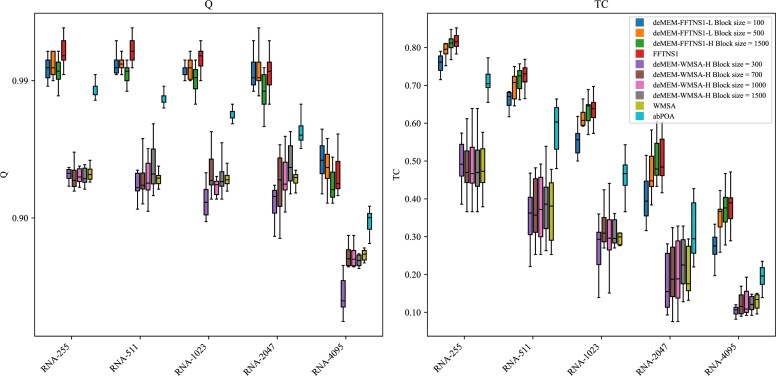
Q and TC scores in RNA simulated tests. Only the enhanced deMEM methods are shown.

**Figure 7:**
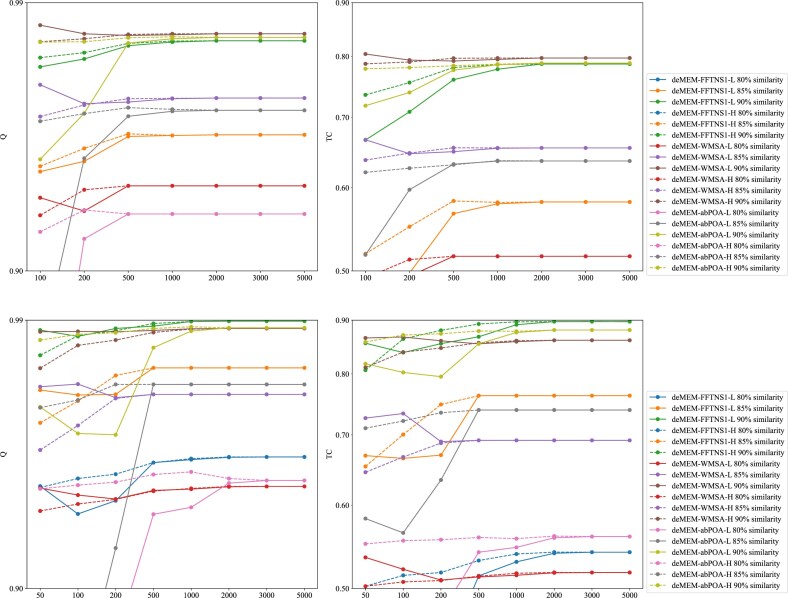
Q and TC scores in two similarity datasets, showing only Q and TC scores with 80%~90% similarity. Upper: mt-similarity dataset; lower: SARS-CoV-2 similarity dataset.

Simulated datasets provide real alignment results, allowing for a direct comparison between alignment results and actual alignments. We evaluated our method on 2 simulation datasets, as shown in Fig. 6 and Fig. 7. Fig. 6 presents the Q and TC score improvements between subalignment methods and raw alignment methods. Our method achieved a slightly higher-quality alignment result compared to WMSA raw methods in simulated RNA test cases. Fig. 7 highlights the impact of small MEM blocks on alignment accuracy. The results indicate that incorporating small MEM blocks enhances both Q and TC scores, leading to improved overall alignment quality.

## Conclusion

In this article, we introduced deMEM, a novel framework for MSA that supports horizontal and vertical problem decomposition based on MEMs. The method follows a 3-step approach: first, deMEM identifies MEMs and clusters with all input sequences using the de Bruijn graph. Next, sequences in clusters are aligned into profiles by the divide-and-conquer framework based on MEMs. Lastly, profiles are progressively aligned to obtain the final MSA. The core of deMEM is the divide-and-conquer framework, where MEMs are used to split the alignment problem into smaller subproblems in both vertical and horizonal conditions. Experiments demonstrated that our method outperforms comparable tools on large-scale datasets, such as a thousand MPoX genomes, achieving approximately 50.3% and 32.0% higher SP scores than FAME and FMAlign2, respectively. Additionally, experiments on simulated data indicate that deMEM achieves improved alignment accuracy with slight enhancements. As a future work, we plan to improve the deMEM method to optimize the space usage to support extremely long and large-scale sequence alignment. Moreover, we will focus on protein sequence alignment to extend the application of our method.

The deMEM package is freely available at GitHub [45]. It has been tested on the Linux and Windows. This package is also available on conda.

## Availability of source code and requirements

- Project name: deMEM
- Project homepage: https://github.com/malabz/deMEM
- Operating system(s): Linux (Recommended) & Windows
- Programming language: C++
- Other requirements: Anaconda (Recommended)
- License: MIT
- Any restrictions to use by nonacademics: License needed

## Supplementary Material

giaf163_Supplemental_Files

giaf163_Authors_Response_To_Reviewer_Comments_Original_Submission

giaf163_GIGA-D-25-00459_Original_Submission

giaf163_GIGA-D-25-00459_Revision_1

giaf163_Reviewer_1_Report_Original_Submission Andreas Grigorjew -- 11/26/2025

giaf163_Reviewer_2_Report_Original_Submission Antoine Limasset, Ph.D -- 12/4/2025

## Data Availability

The source code is publicly available at GitHub [45], and the datasets are publicly available at Zenodo [46].

## Additional Files

**Supplementary Algorithm S1**. Sequence clustering based on a compressed de Bruijn graph.

**Supplementary Algorithm S2**. Align sequences by MEMs.

**Supplementary Table S1**. Results in real datasets. “Block size” in this table means the maximum SP score aligned by the determined MEM block size. “-” in SP means the corresponding method cannot make the alignment. We tested FAME as the default argument.

## Abbreviations

BWT: Burrows–Wheeler transform; DFS: Depth First Search; LCP: Longest Common Prefix; MEM: maximum exact match; MSA: multiple sequence alignment; POA: Partial Order Alignment; Q: Quality; SIMD: Single Instruction Multiple Data; SP: Sum of Pairs; TC: Total Columns.

## References

[1] Wang GH, Liu YL, Zhu DX, et al. Bioinformatics methods and biological interpretation for next-generation sequencing data. Biomed Res Int. 2015;2015:1–2. 10.1155/2015/690873. PMC4576002 26436095

[2] Yin C, Wang R, Qiao J, et al. NanoCon: contrastive learning-based deep hybrid network for nanopore methylation detection. Bioinformatics. 2024;40:btae046. 10.1093/bioinformatics/btae046. 38305428 PMC10873575

[3] Chitsaz H, Yee-Greenbaum JL, Tesler G, et al. Efficient de novo assembly of single-cell bacterial genomes from short-read data sets. Nat Biotechnol. 2011;29:915–21. 10.1038/nbt.1966. 21926975 PMC3558281

[4] Sohn JI, Nam JW. The present and future of de novo whole-genome assembly. Brief Bioinform. 2018;19:23–40. 10.1093/bib/bbw096. 27742661

[5] Muyas F, Sauer CM, Valle-Inclan JE, et al. De novo detection of somatic mutations in high-throughput single-cell profiling data sets. Nat Biotechnol. 2024;42:758–67. 10.1038/s41587-023-01863-z. 37414936 PMC11098751

[6] Tian Q, Zhang P, Zhai Y, et al. Application and comparison of machine learning and database-based methods in taxonomic classification of high-throughput sequencing data. Genome Biol Evolut. 2024;16:evae102. 10.1093/gbe/evae102. PMC11135637 38748485

[7] Wang L, Ding Y, Tiwari P, et al. A deep multiple kernel learning-based higher-order fuzzy inference system for identifying DNA N4-methylcytosine sites. Inform Sci. 2023;630:40–52. 10.1016/j.ins.2023.01.149.

[8] Chao J, Tang F, Xu L. Developments in algorithms for sequence alignment: a review. Biomolecules. 2022;12:546. 10.3390/biom12040546. 35454135 PMC9024764

[9] Zou Q, Hu Q, Guo M, et al. HAlign: fast multiple similar DNA/RNA sequence alignment based on the centre star strategy. Bioinformatics. 2015;31:2475–81. 10.1093/bioinformatics/btv177. 25812743

[10] Wan S, Zou Q. HAlign-II: efficient ultra-large multiple sequence alignment and phylogenetic tree reconstruction with distributed and parallel computing. Algorithms Mol Biol. 2017;12:25. 10.1186/s13015-017-0116-x. 29026435 PMC5622559

[11] Tang F, Chao J, Wei Y, et al. HAlign 3: fast multiple alignment of ultra-large numbers of similar DNA/RNA sequences. Mol Biol Evol. 2022;39:msac166. 10.1093/molbev/msac166. 35915051 PMC9372455

[12] Zhou T, Zhang P, Zou Q, et al. HAlign 4: a new strategy for rapidly aligning millions of sequences. Bioinformatics. 2024;40:btae718. 10.1093/bioinformatics/btae718. 39607773 PMC11646084

[13] Higgins DG, Sharp PM. CLUSTAL: a package for performing multiple sequence alignment on a microcomputer. Gene. 1988;73:237–44. 10.1016/0378-1119(88)90330-7. 3243435

[14] Katoh K, Misawa K, Kuma K, et al. MAFFT: a novel method for rapid multiple sequence alignment based on fast fourier transform. Nucleic Acids Res. 2002;30:3059–66. 10.1093/nar/gkf436. 12136088 PMC135756

[15] Edgar RC . MUSCLE: multiple sequence alignment with high accuracy and high throughput. Nucleic Acids Res. 2004;32:1792–97. 10.1093/nar/gkh340. 15034147 PMC390337

[16] Deorowicz S, Debudaj-Grabysz A, Gudys A. FAMSA: fast and accurate multiple sequence alignment of huge protein families. Sci Rep. 2016;6:33964. 10.1038/srep33964. 27670777 PMC5037421

[17] Wei Y, Zou Q, Tang F, et al. WMSA: a novel method for multiple sequence alignment of DNA sequences. Bioinformatics. 2022;38:5019–25. 10.1093/bioinformatics/btac658. 36179076

[18] Lyras DP, Metzler D. ReformAlign: improved multiple sequence alignments using a profile-based meta-alignment approach. BMC Bioinf. 2014;15:265. 10.1186/1471-2105-15-265. PMC4133627 25099134

[19] Zhai Y, Chao J, Wang Y, et al. TPMA: a two pointers meta-alignment tool to ensemble different multiple nucleic acid sequence alignments. PLoS Comput Biol. 2024;20:e1011988. 10.1371/journal.pcbi.1011988. 38557416 PMC11008887

[20] Zhai Y, Zhou T, Wei Y, et al. ReAlign-N: an integrated realignment approach for multiple nucleic acid sequence alignment, combining global and local realignments. NAR Genomics Bioinformatics. 2024;6:lqae170. 10.1093/nargab/lqae170. 39703429 PMC11655299

[21] Liu Y, Shen X, Gong Y, et al. Sequence alignment/map format: a comprehensive review of approaches and applications. Briefings Bioinf. 2023;24:bbad320. 10.1093/bib/bbad320. 37668049

[22] Li H, Liu B. BioSeq-Diabolo: biological sequence similarity analysis using Diabolo. PLoS Comput Biol. 2023;19:e1011214. 10.1371/journal.pcbi.1011214. 37339155 PMC10313010

[23] Li H, Pang Y, Liu B. BioSeq-BLM: a platform for analyzing DNA, RNA, and protein sequences based on biological language models. Nucleic Acids Res. 2021;49:e129. 10.1093/nar/gkab829. 34581805 PMC8682797

[24] Li H . Minimap2: pairwise alignment for nucleotide sequences. Bioinformatics. 2018;34:3094–100. 10.1093/bioinformatics/bty191. 29750242 PMC6137996

[25] Naznooshsadat E, Elham P, Ali S-Z. FAME: fast and memory efficient multiple sequences alignment tool through compatible chain of roots. Bioinformatics. 2020;36:3662–68. 10.1093/bioinformatics/btaa175. 32170927

[26] Liu H, Zou Q, Xu Y. A novel fast multiple nucleotide sequence alignment method based on FM-index. Brief Bioinform. 2022;23:bbab519. 10.1093/bib/bbab519. 34893794

[27] Zhang P, Liu H, Wei Y, et al. FMAlign2: a novel fast multiple nucleotide sequence alignment method for ultralong datasets. Bioinformatics. 2024;40:btae014. 10.1093/bioinformatics/btae014. 38200554 PMC10809904

[28] Zhang Y, Waterman MS. An Eulerian path approach to global multiple alignment for DNA sequences. J Comput Biol. 2003;10:803–19. 10.1089/106652703322756096. 14980012

[29] Zhang Y, Waterman MS. An eulerian path approach to local multiple alignment for DNA sequences. Proc Natl Acad Sci USA. 2005;102:1285–90. 10.1073/pnas.0409240102. 15668398 PMC547885

[30] De Bruijn NG . A combinatorial problem. Proc K Ned Akad Wet. 1946;49:758–64.

[31] Lee C, Grasso C, Sharlow MF. Multiple sequence alignment using partial order graphs. Bioinformatics. 2002;18:452–64. 10.1093/bioinformatics/18.3.452. 11934745

[32] Gao Y, Liu Y, Ma Y, et al. abPOA: an SIMD-based C library for fast partial order alignment using adaptive band. Bioinformatics. 2021;37:2209–11. 10.1093/bioinformatics/btaa963. 33165528

[33] Liu B, Guo H, Brudno M, et al. deBGA: read alignment with de Bruijn graph-based seed and extension. Bioinformatics. 2016;32:3224–32. 10.1093/bioinformatics/btw371. 27378303

[34] Marcus S, Lee H, Schatz MC. SplitMEM: a graphical algorithm for pan-genome analysis with suffix skips. Bioinformatics. 2014;30:3476–83. 10.1093/bioinformatics/btu756. 25398610 PMC4253837

[35] Baier U, Beller T, Ohlebusch E. Graphical pan-genome analysis with compressed suffix trees and the Burrows-Wheeler transform. Bioinformatics. 2016;32:497–504. 10.1093/bioinformatics/btv603. 26504144

[36] Zhao M, Lee WP, Garrison EP, et al. SSW library: an SIMD Smith-Waterman C/C++ library for use in genomic applications. PLoS One. 2013;8:e82138. 10.1371/journal.pone.0082138. 24324759 PMC3852983

[37] Tarjan RE . A class of algorithms which require nonlinear time to maintain disjoint sets. J Comput System Sci. 1979;18:110–27. 10.1016/0022-0000(79)90042-4.

[38] Khan Z, Bloom JS, Kruglyak L, et al. A practical algorithm for finding maximal exact matches in large sequence datasets using sparse suffix arrays. Bioinformatics. 2009;25:1609–16. 10.1093/bioinformatics/btp275. 19389736 PMC2732316

[39] Smith TF, Waterman MS. Identification of common molecular subsequences. J Mol Biol. 1981;147:195–97. 10.1016/0022-2836(81)90087-5. 7265238

[40] Wei Y, Zhou T, Zhai Y, et al. FORAlign: accelerating gap-affine DNA pairwise sequence alignment using FOR-blocks based on Four Russians approach with linear space complexity. Brief Bioinform. 2025;26:bbaf061. 10.1093/bib/bbaf061. PMC11846685 39987460

[41] Hirschberg DS . A linear space algorithm for computing maximal common subsequences. Commun ACM. 1975;18:341–43. 10.1145/360825.360861.

[42] Kong X, Shen C, Tang J. CUK-Band: a CUDA-based multiple genomic sequence alignment on GPU. Singapore: Springer Nature; 2024:84–95.

[43] Ma Y, Chen M, Bao Y, et al. MPoxVR: a comprehensive genomic resource for monkeypox virus variant surveillance. Innovation. 2022;3:100296. 10.1016/j.xinn.2022.100296. 36039088 PMC9418550

[44] Chen J, Chao J, Liu H, et al. WMSA 2: a multiple DNA/RNA sequence alignment tool implemented with accurate progressive mode and a fast win-win mode combining the center star and progressive strategies. Brief Bioinform. 2023;24:bbad190. 10.1093/bib/bbad190. 37200156

[45] Wei Y, Huang Z, Zhang P, et al. deMEM: a novel divide-and-conquer framework based on de Bruijn graph for scalable multiple sequence alignment. GitHub. 2025.; https://github.com/malabz/deMEM. Accessed 15 December 2025.

[46] Wei Y, Huang Z, Zhang P, et al. deMEM: a novel divide-and-conquer framework based on de Bruijn graph for scalable multiple sequence alignment. Zenodo. 2025. 10.5281/zenodo.14989520. Accessed 15 December 2025. PMC12878729 41489486

