# Supplementary material for: deMEM: a novel divide-and-conquer framework based on de Bruijn graph for scalable multiple sequence alignment
Source: Gigascience. 2026 Jan 5;15:giaf163. doi: 10.1093/gigascience/giaf163 (PMC12878729; doi:10.1093/gigascience/giaf163)
Supplement: giaf163_Supplemental_Files [file giaf163_supplemental_files.zip › Algorithms_supplementary_material-20251215.docx]

# Supplementary Materials for

deMEM: a novel divide-and-conquer framework based on de Bruijn graph for scalable multiple sequence alignment

Yanming Wei^1,2^, Zhaoyang Huang^1^, Pinglu Zhang^2,3^, Yizheng Wang^2,3^, Yan Li^4^, Liang Yu^1,*,¶^, Quan Zou^2,3,*,¶^

^1^School of Computer Science and Technology, Xidian University, Xi’an 710126, Shaanxi, China

^2^Yangtze Delta Region Institute (Quzhou), University of Electronic Science and Technology of China, Quzhou 324003, Zhejiang, China

^3^Institute of Fundamental and Frontier Sciences, University of Electronic Science and Technology of China, Chengdu 610054, Sichuan, China

^4^School of Management, Xi'an Polytechnic University, Xi’an 710121, Shaanxi, China

*To whom correspondence should be addressed. Email: [lyu@xidian.edu.cn](mailto:lyu@xidian.edu.cn), [zouquan@nclab.net](mailto:zouquan@nclab.net)

^¶^These authors should be considered as co-corresponding authors.

## The pseudo code of deMEM

As demonstrated in the “Sequence Clustering by de Bruijn Graph” section, we present the pseudocode for the modified BWT-enhanced SplitMEM [1] to illustrate the sequence clustering process:

| Algorithm S1: Sequence clustering based on compressed de Bruijn graph. |
| --- |
| Input: Multiple sequences$S_{1},\ldots,S_{n}$, summary length of these sequences $L$, k-mer size $k$  Output: Clusters $C_{1},C_{2},\ldots C_{m}$ contains $n$ sequences  Cluster_sequences($S_{1},\ldots,S_{n}$) {  create_BIT_vectors($k,BWT, G$); // this function represents Algorithm 2 in BWT-enhanced SplitMEM line 2-8  $SA$=$S_{1}\#\ldots\#S_{n}\$$; // add separate symbol “#” and end of string symbol “$” to concatenate input sequences  dsu = new array($n$); // define disjoint-set data structure  for(i = 0; i < $n$; ++ i) dsu[i] = i; // initialize each element in the disjoint set to itself  for(p = $L$; p >= 2; --p) {  number = which_cluster(${SA}_{p}$); // this function represents Algorithm 2 in [1] line 10-16  if(number != ⊥) {  G[cur].posList.push_front(p);  G[number].adjList.push_front(cur);  G[number].len = $k$;  dsu_merge(cur, number); // found same MEM and merge two disjoint-set unions  cur = number;  }  else G[cur].len++;  }  clusters = new array($n$, vector()); // The variable “clusters” is two-dimensional vector  for (i = 0; i < $n$; ++ i) clusters[dsu[i]].append(i); // set sequence i to cluster dsu[i]  return clusters;  } |

As demonstrated in the “Align by sorted MEMs” section, we present the pseudocode for the core algorithm of deMEM in Algorithm S2, to illustrate the alignment process:

| Algorithm S2: Align sequences by MEMs |
| --- |
| Input: Multiple sequences$S_{1},\ldots,S_{n}$, interval for these sequences $\left[ B_{1},E_{1} \right), \ldots,\left[ B_{n}, E_{n} \right)$, determined MEM block $\boldsymbol{MX}$ with $\left\vert\boldsymbol{MX} \right\vert=m (m\leq n)$, other MEMs $\boldsymbol{MX}_{1},\boldsymbol{MX}_{2},\ldots,\boldsymbol{MX}_{b}$  Output: Aligned sequences, which aligned intervals $\left[ B_{1},E_{1} \right), \ldots,\left[ B_{n}, E_{n} \right)$ for corresponding sequences $S_{1},\ldots,S_{n}$  BlockAlign($S_{1},\ldots,S_{n},B_{1},\ldots,B_{n},E_{1},\ldots,E_{n},b,\boldsymbol{MX}_{1},\ldots\boldsymbol{MX}_{b}$) {  if(b == 0) CallSubAlignment($S_{1},\ldots,S_{n},B_{1},\ldots,B_{n},E_{1},\ldots,E_{n}$);  else {  SortbyArea($\boldsymbol{MX}_{1},\ldots,\boldsymbol{MX}_{b}$);  Align($S_{1},\ldots,S_{n},B_{1},\ldots,B_{n},E_{1},\ldots,E_{n},\boldsymbol{MX}_{1},\ldots\boldsymbol{MX}_{b}$);  }  }  Align($S_{1},\ldots,S_{n},B_{1},\ldots,B_{n},E_{1},\ldots,E_{n},\boldsymbol{MX},\boldsymbol{MX}_{1},\boldsymbol{MX}_{2},\ldots,\boldsymbol{MX}_{M}$) {  // Part 1: use SSW to find sequences  // $\boldsymbol{MX}$ cover sequences $S_{1},\ldots,S_{m}$. Formally, $\boldsymbol{MX}=\{L, \left( 1, L_{1} \right), \ldots,(m,L_{m})\}$  $\boldsymbol{MX}^{'}$=FindSimilar($\boldsymbol{MX},\left[ S_{m+1},\ldots,S_{n} \right],\left[ B_{m+1},E_{m+1} \right), \ldots,\left[ B_{n}, E_{n} \right)$) // Find similar fragments in remaining sequences  AlignBlock($\boldsymbol{MX}^{'}$); // Align MEM block with similar blocks, $\boldsymbol{MX}'$ covers sequences $S_{1},\ldots,S_{m+k}$  // Part 2: divide other blocks into left blocks, right blocks or down blocks  for (i=1; i<=$M$; ++i) { // check other MEMs  for ($I_{j}=[{LL}_{j},{RR}_{j})$ in $\boldsymbol{MX}_{i}$) {  if ($S_{j}$ in $\boldsymbol{MX}^{'}$) {  if ($I_{j}$ across $\left[ L_{j},R_{j} \right)$) $I_{j}$.SplitandAppend(this_left, this_right);  else if ($I_{j}$ in left) this_left.append($I_{j}$);  else this_right.append($I_{j}$);  }  else this_down.append($I_{j}$);  }  // check the size of parted blocks, if size < 2, drop it  if(this_left.size>=2) left_blocks.append(this_left);  if(this_right.size>=2) right_blocks.append(this_right);  if(this_down.size>=2) down_blocks.append(this_down);  }  // Part 3: call sub-alignment process recursively  BlockAlign($S_{1},\ldots,S_{m+k},B_{1},\ldots,B_{m+k},L_{1},\ldots,L_{m+k},$ left_blocks.size, left_blocks);  BlockAlign($S_{1},\ldots,S_{m+k},R_{1},\ldots,R_{m+k},E_{1},\ldots,E_{m+k},$ right_blocks.size, right_blocks);  if ($m+k\neq n$) {  BlockAlign($S_{m+k+1},\ldots,S_{n}, B_{m+k+1},\ldots,B_{n}, E_{m+k+1},\ldots,E_{n}$, down_blocks.size, down_blocks);  ProfileProfileAlign($\left[ S_{1},\ldots,S_{m+k},B_{1},\ldots,B_{m+k},E_{1},\ldots,E_{m+k} \right]$, $[S_{m+k+1},\ldots,S_{n},B_{m+k+1},\ldots, B_{n}, E_{m+k+1},\ldots,E_{n}]$); // Profile-profile alignment of two parts  }  } |

It’s worth noting that, in Algorithm S2, the sequence interval $I_{j}=[{LL}_{j},{RR}_{j})$ in Part 2 may across the MEM $\boldsymbol{MX}\mathbf{'}$ interval $\left[ L_{j}, R_{j} \right)$, and the effective range in the alignment process of sequence $S_{j}$ is $\left[ B_{j},E_{j} \right)$. Therefore, the interval $[{LL}_{j},{RR}_{j})$ must be split, resulting in two sub-intervals: this_left=$[B_{j},L_{j})$, this_right=$[R_{j},E_{j})$.

## References

1. Baier U, Beller T and Ohlebusch E. Graphical pan-genome analysis with compressed suffix trees and the Burrows-Wheeler transform. Bioinformatics. 2016;32 4:497-504. doi:10.1093/bioinformatics/btv603.
