# Supplementary material for: deMEM: a novel divide-and-conquer framework based on de Bruijn graph for scalable multiple sequence alignment
Source: Gigascience. 2026 Jan 5;15:giaf163. doi: 10.1093/gigascience/giaf163 (PMC12878729; doi:10.1093/gigascience/giaf163)
Supplement: giaf163_Supplemental_Files [file giaf163_supplemental_files.zip › Table S1_supplementary_material.docx]

Table S1. Results in real datasets. “Block size” in this table means the maximum SP score aligned by the determined MEM block size. “-” in SP means the corresponding method can not make alignment. We tested FAME as the default argument

| Method Name | mt1x | | | | | | | mt20x | | | | | | | Complete156 | | | | | |
| --- | --- | --- | --- | --- | --- | --- | --- | --- | --- | --- | --- | --- | --- | --- | --- | --- | --- | --- | --- | --- |
|  | Block Size | | SP | | Time  /s | Memory  /MB | | Block Size | | SP | Time  /s | | Memory  /MB | | Block Size | SP | | Time  /s | | Memory  /MB |
| FAME | - | | -212.1 | | 9.0 | 119.2 | | - | | -211.9 | **35.4** | | 2109.7 | | 7 | -403.3 | | 4.5 | | 99.1 |
| FMAlign2 | 20 | | -150.7 | | **5.9** | 314.3 | | 500 | | -152.3 | 1202.1 | | 4890.1 | | 1000 | -261.6 | | **1.2** | | 105.8 |
| deMEM-abPOA-L | 10 | | -154.6 | | 88.0 | 1465.5 | | 20000 | | -158.2 | 882.0 | | 3827.0 | | 200 | -299.2 | | 12.6 | | 622.1 |
| deMEM-abPOA-H | 5000 | | -156.1 | | 20.4 | 354.3 | | 5000 | | -154.1 | 823.8 | | 1065.3 | | 800 | -338.2 | | 3.7 | | 73.3 |
| deMEM-FFTNS1-L | 50 | | -153.6 | | 215.0 | 2402.5 | | 15000 | | -152.2 | 270289.0 | | 128959.8 | | 20000 | -273.8 | | 32.9 | | 326.9 |
| deMEM-FFTNS1-H | 100 | | **-148.2** | | 67.1 | 337.3 | | 100 | | **-148.2** | 12810.0 | | 3683.1 | | 7000 | -261.8 | | 7.7 | | **42.2** |
| deMEM-WMSA-L | 5000 | | -149.5 | | 432.1 | 1068.9 | | 50 | | -149.5 | 1133.8 | | 50371.2 | | 20000 | **-244.5** | | 31.7 | | 352.7 |
| deMEM-WMSA-H | 5000 | | -149.1 | | 27.6 | **56.2** | | 5000 | | -149.0 | 581.2 | | **1065.3** | | 12000 | -244.8 | | 8.7 | | 49.0 |
| Method Name | Mix1t | | | | | | | 23sr | | | | | | | *Variola virus* | | | | | |
|  | Block Size | | SP | | Time  /s | Memory  /MB | | Block Size | | SP | Time  /s | | Memory  /MB | | Block Size | SP | | Time  /s | | Memory  /MB |
| FAME | - | | - | | - | - | | - | | - | - | | - | | - | -2913.3 | | 8.6 | | 37.7 |
| FMAlign2 | 2000 | | -46860.8 | | 1654.8 | 2290.6 | | 1000 | | -2367.0 | 12.8 | | 178.5 | | 100 | -2700.3 | | 3.4 | | 20.6 |
| deMEM-abPOA-L | 50 | | -14490.2 | | 1483.1 | 23572.9 | | 1200 | | -4348.5 | 61.9 | | 394.5 | | 800 | -2363.7 | | 4.1 | | 254.3 |
| deMEM-abPOA-H | 100 | | -51592.0 | | 3094.6 | 34026.5 | | 1200 | | -3377.5 | 2.9 | | **44.0** | | 1000 | -2203.7 | | **1.9** | | 76.7 |
| deMEM-FFTNS1-L | 100 | | -18886.8 | | 11462.0 | 68385.3 | | 2000 | | -2367.0 | 10.5 | | 526.2 | | 5000 | -2696.0 | | 39.5 | | 67.3 |
| deMEM-FFTNS1-H | 100 | | -18886.8 | | 10907.0 | 67101.7 | | 2000 | | -2367.0 | 11.1 | | 553.1 | | 800 | -2703.2 | | 77.2 | | 7.8 |
| deMEM-WMSA-L | 100 | | **-13036.7** | | 79.0 | 4677.1 | | 20 | | **-1742.6** | 10.0 | | 403.5 | | 20000 | **-2164.0** | | 20.3 | | 78.9 |
| deMEM-WMSA-H | 100 | | **-13036.7** | | **75.4** | **4298.9** | | 2000 | | -2408.5 | **2.6** | | 178.3 | | 500 | -2712.8 | | 7.5 | | **7.4** |
| Method Name | MPoX | | | | | | | *Mycoplasma bovis* | | | | | | | *Streptococcus pneumonia* | | | | | |
|  | Block Size | | SP | | Time  /s | Memory  /MB | | Block Size | | SP | Time  /s | | Memory  /MB | | Block Size | SP | | Time  /s | | Memory  /MB |
| FAME | - | | -206051.9 | | **36.8** | **1770.2** | | - | | - | - | | - | | - | - | | - | | - |
| FMAlign2 | 10 | | -150564.9 | | 510629.0 | 9512.8 | | 500 | | **-7127.8** | **2.4** | | 60.2 | | 2000 | **-889430.7** | | 2748.4 | | **217.5** |
| deMEM-abPOA-L | - | | Out of Memory | | | | | 500 | | -8062.0 | 29.8 | | 2102.9 | | - | Out of Memory | | | | |
| deMEM-abPOA-H | 10000 | | -387056.7 | | 459.3 | 4217.9 | | 5000 | | -7872.8 | 60.7 | | 15546.7 | | 1000 | -2660563.2 | | **319.8** | | 11752.0 |
| deMEM-FFTNS1-L | 12000 | | -182553.8 | | 52783.0 | 48684.3 | | 10000 | | -7149.0 | 339.2 | | 718.1 | | 1000 | -1225436.5 | | 1185.8 | | 520.5 |
| deMEM-FFTNS1-H | 10000 | | -398205.1 | | 1571.5 | 8288.8 | | 5000 | | -7130.3 | 199.0 | | **52.9** | | 10000 | -1826520.3 | | 27839.0 | | 1671.0 |
| deMEM-WMSA-L | 12000 | | **-102311.0** | | 41257.0 | 99238.5 | | 5000 | | -7342.7 | 3015.9 | | 319.0 | | 1000 | -901459.0 | | 79250.0 | | 150746.9 |
| deMEM-WMSA-H | 10000 | | -436952.5 | | 1642.2 | 4440.4 | | 5000 | | -7583.5 | 27.0 | | 96.5 | | 10000 | -1364741.2 | | 15354.0 | | 127781.6 |
| Method Name | | *Escherichia coli* | | | | | | | | | | *Nerisseria meningitidis* | | | | | | | | |
|  |  | Block  Size | | SP | | | Time  /s | | Memory  /MB | | | Block Size | | SP | | | Time  /s | | Memory  /MB | |
| FAME | | - | | - | | | - | | - | | | - | | - | | | - | | - | |
| FMAlign2 | | 100 | | -2894827 | | | **727** | | 12773 | | | 2000 | | **-2980447** | | | 166637 | | 2767 | |
| deMEM-abPOA-L | | - | | Out of Memory | | | | | | | | - | | Out of Memory | | | | | | |
| deMEM-abPOA-H | | 500 | | -16962803 | | | 358 | | 259807 | | | 1000 | | -8853709 | | | **515** | | 105222 | |
| deMEM-FFTNS1-L | | 2000 | | -1663531 | | | 4757 | | **1487** | | | 2000 | | -3281288 | | | 60664 | | 1347 | |
| deMEM-FFTNS1-H | | 500 | | -16868812 | | | 1555 | | 2040 | | | 5000 | | -4023464 | | | 39066 | | **678** | |
| deMEM-WMSA-L | | 2000 | | **-1558240** | | | 86174 | | 508726 | | | - | | Out of Memory | | | | | | |
| deMEM-WMSA-H | | 500 | | -16744612 | | | 2833 | | 156754 | | | 2000 | | -6102398 | | | 6624 | | 210283 | |
