# Supplementary material for: deMEM: a novel divide-and-conquer framework based on de Bruijn graph for scalable multiple sequence alignment
Source: Gigascience. 2026 Jan 5;15:giaf163. doi: 10.1093/gigascience/giaf163 (PMC12878729; doi:10.1093/gigascience/giaf163)
Supplement: giaf163_GIGA-D-25-00459_Original_Submission [file giaf163_giga-d-25-00459_original_submission.pdf]

GigaScience

deMEM: a novel divide-and-conquer framework based on de Bruijn graph for scalable multiple sequence alignment

--Manuscript Draft--

**Manuscript Number:**	GIGA-D-25-00459
**Full Title:**	deMEM: a novel divide-and-conquer framework based on de Bruijn graph for scalable multiple sequence alignment
**Article Type:**	Technical Note
**Funding Information:**	National Natural Science Foundation of China (62472344) — Dr. Liang Yu<br>National Natural Science Foundation of China (62452107) — Dr. Quan Zou<br>National Natural Science Foundation of China (62072353) — Dr. Liang Yu<br>National Natural Science Foundation of China (62272065) — Dr. Liang Yu<br>Xidian University Specially Funded Project for Interdisciplinary Exploration (TZJH2024027) — Dr. Liang Yu
**Abstract:**	Multiple sequence alignment (MSA) continues to be a central challenge in comparative genomics, where the quality of alignment plays a crucial role in determining the accuracy of downstream analyses. However, the challenge of large-scale alignment remains significant. This paper introduces deMEM, a novel and effective framework for DNA multiple sequence alignment, which enables existing MSA methods such as MAFFT, to handle extremely large sequences. deMEM is a three-stage alignment process: (i) representing Maximum Exact Matches using a de Bruijn graph and clustering them based on their area; (ii) employing a novel divide-and-conquer framework for alignment; (iii) profile-profile alignment between different clusters. deMEM enables existing methods like MAFFT to align an extremely large number of sequences, including long sequences that cannot be directly aligned, such as those in a dataset of a thousand monkeypox virus genomes. The deMEM package is free and available at https://github.com/malabz/deMEM.
**Corresponding Author:**	Liang Yu<br>Xidian University<br>Xi'an, Shaanxi CHINA
**Corresponding Author Secondary Information:**	
**Corresponding Author's Institution:**	Xidian University
**Corresponding Author's Secondary Institution:**	
**First Author:**	Yanming Wei
**First Author Secondary Information:**	
**Order of Authors:**	Yanming Wei
	Zhaoyang Huang
	Pinglu Zhang
	Yizheng Wang
	Yan Li
	Liang Yu

Powered by Editorial Manager® and ProduXion Manager® from Aries Systems Corporation

	Quan Zou
**Order of Authors Secondary Information:**	
**Additional Information:**	
**Question**	**Response**
Are you submitting this manuscript to a special series or article collection?	No
**Experimental design and statistics**<br><br>Full details of the experimental design and statistical methods used should be given in the Methods section, as detailed in our Minimum Standards Reporting Checklist. Information essential to interpreting the data presented should be made available in the figure legends.<br><br>Have you included all the information requested in your manuscript?	Yes
**Resources**<br><br>A description of all resources used, including antibodies, cell lines, animals and software tools, with enough information to allow them to be uniquely identified, should be included in the Methods section. Authors are strongly encouraged to cite Research Resource Identifiers (RRIDs) for antibodies, model organisms and tools, where possible.<br><br>Have you included the information requested as detailed in our Minimum Standards Reporting Checklist?	Yes
**Availability of data and materials**<br><br>All datasets and code on which the conclusions of the paper rely must be either included in your submission or deposited in publicly available repositories (where available and ethically appropriate), referencing such data using a unique identifier in the references and in	Yes

the “Availability of Data and Materials” section of your manuscript.<br><br>Have you have met the above requirement as detailed in our Minimum Standards Reporting Checklist?	
GigaScience has policies and guidelines in place for the use of generative AI-writing tools such as ChatGPT. If you have used such writing tools to assist with writing the manuscript this must be declared and cited in the text. Authors should not list AI-writing tools and other AI-assisted technologies as an author or co-author and should acknowledge that they are fully responsible for text generated or refined by AI-writing tools.<p><br><br>A summary of use (particularly in the introduction or among methods) needs to be included at the end of the paper, and the outputs should also be included as a supplementary file hosted in GigaDB or other open repositories. Please read our guidelines for more information. <p><br><br>By submitting to GigaScience, you are aware of the journal's AI-writing tools policy, and if you have declared use of such tools below, you have acknowledged this where appropriate in your manuscript and have made a summary of use and outputs available. <p><br><b>AI-assisted writing tools have been used in the preparation of this manuscript?	No

Powered by Editorial Manager® and ProduXion Manager® from Aries Systems Corporation

Manuscript (Tracked Version)
Click here to access/download;Manuscript;deMEM-20251103-diff.docx

deMEM: a novel divide-and-conquer framework based on de Bruijn graph for scalable multiple sequence alignment

Yanming Wei[1,2], Zhaoyang Huang[1], Pinglu Zhang[2,3], Yizheng Wang[2,3], Yan Li[4], Liang Yu[1,*,¶], Quan Zou[2,3,*,¶]

[1] School of Computer Science and Technology, Xidian University, Xi'an 710126, China
[2] Yangtze Delta Region Institute (Quzhou), University of Electronic Science and Technology of China, Quzhou 324003, China
[3] Institute of Fundamental and Frontier Sciences, University of Electronic Science and Technology of China, Chengdu 610054, China
[4] School of Management, Xi'an Polytechnic University, Xi'an 710121, Shaanxi, China
*To whom correspondence should be addressed: Email: lyu@xidian.edu.cn and zouquan@nclab.net.
¶These authors should be considered as co-corresponding authors.

Keywords: Multiple Sequence Alignment, Maximum Exact Match, de Bruijn Graph, Parallel Algorithm Design

Abstract

Multiple sequence alignment (MSA) continues to be a central challenge in comparative genomics, where the quality of alignment plays a crucial role in determining the accuracy of downstream analyses. However, the challenge of large-scale alignment remains significant. This paper introduces deMEM, a novel and effective framework for DNA multiple sequence alignment, which enables existing MSA methods such as MAFFT, to handle extremely large sequences. deMEM is a three-stage alignment process: (i) representing Maximum Exact Matches using a de Bruijn graph and clustering them based on their area; (ii) employing a novel divide-and-conquer framework for alignment; (iii) profile-profile alignment between different clusters. deMEM enables existing

methods like MAFFT to align an extremely large number of sequences, including long sequences that cannot be directly aligned, such as those in a dataset of a thousand monkeypox virus genomes. The deMEM package is free and available at https://github.com/malabz/deMEM.

Introduction

Multiple sequence alignment (MSA) is a fundamental problem in bioinformatics. The quality of sequence alignment significantly impacts biological sequence analysis, especially that in next-generation sequencing [1, 2]. MSA results are widely used in various applications, including *de novo* genome assembly [3, 4], detection of single-cell genomes based on sequence alignment [5] and taxonomic ~~affiliation for~~assignment of newly sequenced ~~sequences~~data [6, 7].

In the last few decades, researchers have shown an increased interest on developing efficient MSA methods to enhance alignment accuracy. The guide tree for aligning MSA is a heuristic approach that aligns sequences based on a pre-built guide tree [8]. Guide tree can be categorized into two types: the center star guide tree and the distance estimation tree, with the latter serving as the basis for progressive alignment. The center star guide tree strategy tree has been utilized in HAlign series [9-12], while the progressive alignment method is employed in several tools, like Clustal [13], MAFFT [14], MUSCLE 3 [15] and FAMSA [16]. WMSA [17] combined center star tree and distance-based guide tree for alignment. The center star guide tree can align a large number of sequences with relatively low alignment quality. In contrast, the progressive alignment method generally produces slightly better-quality alignments, though it is still limited by the quality of the guide tree. To improve alignment quality, researchers have developed post-processing methods, such as ReformAlign [18], TPMA [19] and ReAlign-N [20].

To address the challenge of large-scale, high-quality MSA, researchers have developed seed-and-extension strategy and graph-based strategy [21-24]. The seed-and-extension strategy alignment to reduce the MSA problem by focusing on aligning and extending seed regions. Minimap2 [25] employed seed-and-extension strategy for pairwise sequence alignment. FAME [26] designed a state-of-art model for aligning long sequences through three steps: identifying common seeds based on the determined seed patterns, creating chains from seeds, and generating splitting alignments by chains. FMAlign series [27, 28], inspired by FAME, generate the multiple sequences chain by Maximum Exact Matches (MEM) based on FM-index. FMAlign2 further generates MEM based on LCP extension and supports sequence search with MEM. Graph-based alignment methods provide another approach to solve MSA. EulerAlign [29, 30] proposed MSA by generating and aligning sequences with consensus sequence determined by de Bruijn graph [31], and POA [32] proposed another graph representation to express and generate MSA. abPOA [33] significantly enhances computational efficiency through adaptive-band dynamic programming and SIMD parallelization. deBGA [34] utilizes aligning sequence reads based on de Bruijn graph. MEMs are fundamental for constructing de Bruijn graph in MSA. SplitMEM [35] is an efficient method for generating de Bruijn graph for multiple sequences or genomes. Baier et al. [36] enhanced SplitMEM by the Burrows-Wheeler Transform (BWT) to generate MEM, which significantly reduced the time complexity of the process to O(|Σ|), which Σ is the length of all sequences.

Although there are huge numbers of MSA methods, most of these methods have suffered from various methodological limitations. Firstly, guide-tree based methods generally been restricted to the quality of guide tree and the principle "once a gap, always a gap", where awhereby any gap is inserted during progressive profile-profile alignment, it remains fixed and cannot be

~~removed~~corrected or ~~adjusted~~refined in ~~later~~subsequent steps. Secondly, due to the nature of MEM, seed-and-extension strategy-based methods are focusing the high similarity sequence alignment, with little attention of low similarity sequence alignment. Thirdly, POA is ~~limited~~used for ~~finding~~generating consensus sequences, particularly in third-generation sequencing, but is neither considered nor discussed by the sub-alignment methods in the seed-and-extension strategy, which limits the application of POA. Lastly, the research for de Bruijn graph has tended to focus on third-generation reads data analysis rather than MSA.

~~Our method,~~To address these challenges, we developed deMEM, ~~employs the divide-and-conquer strategy achieve~~an efficient and accurate multiple sequence alignment~~.~~ method based on divide-and-conquer strategy. It works by (a) splitting the sequences into clusters using enhanced version of SplitMEM, ~~then~~(b) aligning ~~those~~ clusters into profiles by the MEMs, and ~~finally~~(c) merge profiles by profile-profile alignment. Our method ~~enhance quality and demonstrated superior~~ enhances alignment quality and demonstrates superior performance compared to traditional, seed-and-extension-free MSA strategies like MAFFT [14], WSMA [17] and abPOA [33] ~~in test experiments~~ on low~~ conserved~~ similarity datasets. Additionally, it supports the alignment of extremely long sequences that these seed-and-extension-free methods cannot handle. Furthermore, deMEM ~~made better performance on diving sequences~~outperforms sequence division methods like FMAlign2 [28] and FAME [26]~~,~~ in handling challenging ~~sequence~~ alignment tasks, ~~like the~~such as aligning extremely long sequences.

Methods

The framework of deMEM

Our developed framework is named deMEM. The architecture of deMEM can be described as follows (Figure 1):

- Step 1: Input the sequence file S, convert sequences to k-mer de Bruijn graph representation with determined threshold k, find MEMs and cluster sequences based on MEMs;
- Step 2: We generated n clusters in Step 1. For each cluster $C_i, i = 1,2,\ldots,n$, we align the cluster by our divide-and-conquer framework based on MEMs obtained from step 1. All clusters are aligned into profiles $P_i, i = 1,2,\ldots,n$;
- Step 3: Align n clusters by determined method, like MAFFT profile merge [14], WMSA [17] or abPOA [33].

Sequence Clustering by de Bruijn graph

We utilize a clustering algorithm based on the BWT-enhanced SplitMEM [35] algorithm [36]. For graph construction, we employ a disjoint-set union data structure [37] to represent cluster affiliation of each sequence. In particular, we modify Algorithm 2 in BWT-enhanced SplitMEM [36] to calculate the cluster. The pseudo code of modified algorithm is shown in Algorithm ~~1~~S1.

~~Algorithm 1: Sequence clustering based on compressed de Bruijn graph.~~

~~Input: Multiple sequences $S_1, \ldots, S_n$, k-mer size k~~

~~Output: Clusters $C_1, C_2, \ldots C_m$ contains n sequences~~

~~Cluster_sequences($S_1, \ldots, S_n$) {~~

```
// this part is same as Algorithm 2 in BWT-enhanced SplitMEM [36] line 2-8
dsu = new array(n); // disjoint-set data structure
for(i = 0; i < n; ++i) dsu[i] = i; // Initialize each element in the disjoint set to itself
for(p = len_sum; p >= 2; --p) {
  // this part is same as Algorithm 2 in [36] line 10-16
  if(number != ⊥) {
    G[cur].posList.push_front(p); G[number].adjList.push_front(cur); G[number].len = k;
    dsu_merge(cur, number); // found same MEM and merge two disjoint-set unions
    cur = number; }
  else G[cur].len++; }
}
```

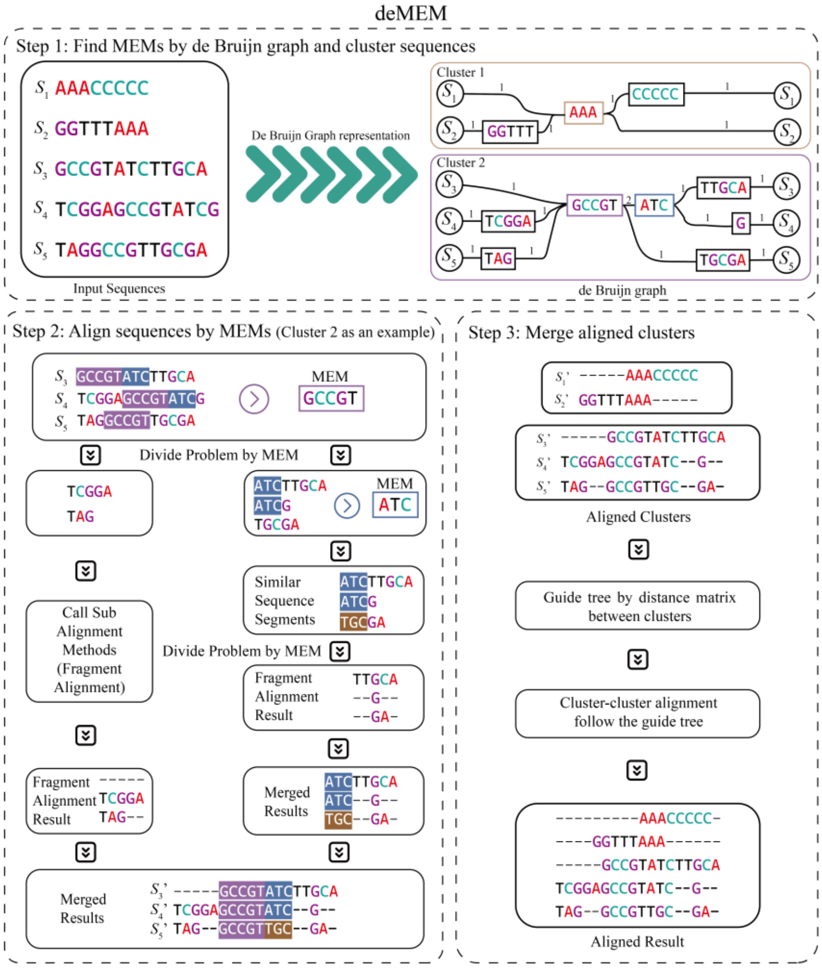

Figure 1: The framework of deMEM. (a) Find MEMs by de Bruijn graph and generate clusters: sequences are read and converted into clusters using SplitMEM [35], which represents the sequences as de Bruijn graph. The number on the edge in the de Bruijn graph represents the edge weight; (b) Align sequences by MEMs: For each cluster, sequences are aligned by a divide-and-conquer framework to process the MEMs. For every MEM, the alignment problem is divided into three subproblems: the left subproblem, the right subproblem, and the down subproblem. For

sequences not covered by any MEM, the SSW library [38] is used to identify similar fragments. If no similar fragment is found, the sequence is processed in the down subproblem. For subproblems without MEMs, external MSA methods such as MAFFT [14], WMSA [17] or abPOA [33] are employed for alignment. The resulting alignments are then merged to form an aligned cluster; (c) Merge aligned clusters to generate MSA: aligned clusters are combined using external profile-profile merge strategy, like MAFFT profile merge, WMSA or abPOA, to generate the final MSA result.

As shown in ~~algorithm 1, the core of~~Algorithm S1, this algorithm ~~is use~~uses disjoint-set data structure to measure the affiliation of sequences. When identical MEMs are found in different sequences, we merge the two disjoint-set unions that represent these sequences. ~~The~~Once the sequence affiliations are established, we apply the original strategy from SplitMEM, which uses Depth-First Search (DFS) to traverse the de Bruijn graph and generate MEMs for each cluster. After generating the MEMs for the clusters, we leverage this information to perform MSA for each cluster using the divide-and-conquer framework.

In conclusion, the time complexity of ~~algorithm 1~~sequence clustering is $O(n(\log\sigma + \alpha(n,n)))$, where σ is the size of alphabet ($\sigma = 4$ in DNA sequences), and $\alpha(n,n)$ is inverse Ackermann function.

~~State-of-the-Art~~ Divide-and-Conquer ~~Algorithm~~Framework for MSA Using MEMs

In this section, we describe the divide-and-conquer ~~algorithm~~framework which the inputs are

MEMs provided by ~~SplitMEM~~Algorithm S1. The core ~~component~~ of deMEM is ~~a~~the divide-and-conquer ~~module implementation, which inputs~~framework, where MEMs ~~for dividing sequences.~~are used to split the alignment problem into smaller subproblems. For ~~every~~each cluster, we sort the MEMs by area, with the ~~highest area~~largest MEM is the first in array. Once ~~the sort~~sorting is complete, ~~we input~~the sorted MEMs ~~and~~with the sequences ~~affiliated by this~~are assigned to cluster ~~to~~, fed the cluster into the divide-and-conquer ~~module~~framework to generate MSA. We start by defining MEM and its area in multiple strings, then define MEM with similar fragments and their area, to support the representation of sequences that lack MEMs but share similarity with them. After defining MEM with similar fragments, we proceed to introduce the alignment process within the divide-and-conquer framework, using the sorted MEMs to produce the final alignment results for the sequences corresponding to the clusters.

The definition of ~~MEMs~~MEM

In this section, we will discuss the definition of MEM in detail. Considering the nature of MEMs, when different sequences share similar strings, our method treats these similar strings as fragments and merges them into the MEM. We first provide a formal definition of MEM, followed by the introduction of the concept of MEM with similar fragments, which allows us to represent these MEMs during the sequence alignment process.

The definition of MEM in two strings is exact matches between two strings that cannot be extended in either direction towards the beginning or end of two strings without allowing for a mismatch [39]. ~~Because of~~Since our ~~question is in~~problem involves multiple sequences, we need to ~~define~~ extend the definition of MEM ~~in~~to accommodate multiple sequences. Definition 1

definesprovides the definition of MEM in multiple strings.:

Definition 1: MEM $\boldsymbol{M}$ in multiple strings $s_1, s_2, \dots, s_n$ is exact matches between multiple strings with match length L and that cannot be extended in either direction towards the beginning or end of multiple strings without allowing for a mismatch, which the intervals $[x_1, x_1+L), [x_2, x_2+L), \dots, [x_n, x_n+L)$ are corresponding with stringstrings $s_1, s_2, \dots, s_n$. In other words, $\boldsymbol{M} = \{L, (1, x_1), (2, x_2), \dots, (n, x_n)\}$, which means the length of MEM is L, the MEM occurs at sequences $s_1, s_2, \dots, s_n$ with begins at $x_1, x_2, \dots, x_n$. The number of strings contains in $\boldsymbol{M}$ is $|\boldsymbol{M}| = n$. If MEM $\boldsymbol{M}$ only occurs at sequence $s_{ID_1}, s_{ID_2}, \dots, s_{ID_d}$, which occurs at the intervals $[x_{ID_1}, x_{ID_1}+L), [x_{ID_2}, x_{ID_2}+L), \dots, [x_{ID_d}, x_{ID_d}+L)$, we define the MEM $\boldsymbol{M} = \{L, (s_{ID_1}, x_{ID_1}), (s_{ID_2}, x_{ID_2}), \dots, (s_{ID_d}, x_{ID_d})\}$, and the length of $\boldsymbol{M}$ is $|\boldsymbol{M}| = d$.

As shown in Definition 1, MEM in multiple strings can be common seeds in measuring the similarity of all sequences, but the distances in exact matches must be measured in sequences. As a result, we need to define the area of MEM for measuring the importance of every MEM. The definition of MEM area in multiple sequences is shown in Definition 2.

Definition 2: The area a of MEM $\boldsymbol{M}$ is defined as formula (1):

$$a = \max_{c}\left(\sum_{i}^{IDs}(L - |x_i - c|)\right) \tag{1}$$

where IDs is the set contains all sequence identifiers in this MEM, c means the "center" place of every sequence in MEM. It's worth noting that, the definition of "center" refers to the position with the highest occurrence frequency in the MEM, which is set as the maximum area to ensure a unique area calculation. In particular, center c can be calculated as formula (2):

$$c = \max\_occur\_times_{i \in IDs}(x_i) \tag{2}$$

where max_occur_times function calculates highest occurrence frequency in the list of start

positions list $\{x_i, i \in IDs\}$. If multiple values have the same maximum frequency, the middle value (i.e., the median among the tied candidates) is selected. If there are two middle values, we compute the corresponding area a for each candidate x_i and choose the one with the maximum area. As shown in formula (2), the meaning of c is the "center" of MEM. If we choose any other $c' \neq c$, we cannot determine the area a uniquely. The example of area calculation is shown in Figure 2.

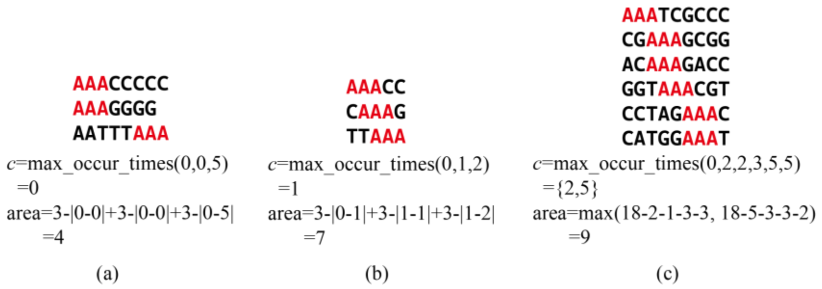

Figure 2: Examples for MEM and MEM area calculation. (a) The definition of MEM is $\boldsymbol{M}_1 = \{3, (0,0), (1,0), (2,5)\}$. The center of $\boldsymbol{M}_1$ is 0, because 0 occurs twice and 5 occurs only once. The area of MEM is calculated by formula (1); (b) The definition of MEM is $\boldsymbol{M}_2 = \{3, (0,0), (1,1), (2,2)\}$. The center of $\boldsymbol{M}_2$ is 1, because 0, 1 and 2 occurs once, we choose the medium number 1 to represent the center; (c) The definition of MEM is $\boldsymbol{M}_3 = \{3, (0,0), (1,2), (2,2), (3,3), (4,5), (5,5)\}$. The center of $\boldsymbol{M}_3$ can be 2 or 5, because both 2 and 5 occur twice in $\boldsymbol{M}_3$. Because 2 and 5 both are medium numbers, we need to calculate area to determine the center. If we choose 2 to be the center of $\boldsymbol{M}_3$, the area is 9; in other words, choose 5 to the center of $\boldsymbol{M}_3$, we can calculate the area of $\boldsymbol{M}_3$ is 5. As a result, we choose 2 to be center of $\boldsymbol{M}_3$.

In Definition 1, the MEM is defined as having the same length and across sequences. However, for the representation of similar fragments, we need to redefine MEM. We refer to this as the “MEM with similar fragments”. The definition of a MEM with similar fragments is provided in Definition

3, and the definition for the area of a MEM with similar fragments is given in Definition 4:

Definition 3: MEM with similar ~~fragment *M*~~fragments $\boldsymbol{MX}$ in multiple strings $s_1, s_2, \dots, s_n$ is exact matches between multiple strings with match length L and that cannot be extended in either direction towards the beginning or end of multiple strings without allowing for a mismatch, after finding similar parts in MEM, strings $s_{n+1}, \dots, s_f$ found similar parts with ~~*M*~~$\boldsymbol{MX}$, which the interval $[x_1, x_1 + L), [x_2, x_2 + L), \dots, [x_n, x_n + L), [x_{n+1}, y_{n+1}), \dots, [x_f, y_f)$ corresponding with string $s_1, s_2, \dots, s_f$. It's worth noting that, the similar parts are identified using the SSW library. In other words, $\boldsymbol{MX} = \{L, (ID_1, x_1, 0), \dots, (ID_n, x_n, 0), (ID_{n+1}, x_{n+1}, y_{n+1} - x_{n+1} - L), \dots, (ID_f, x_f, y_f - x_f - L)\}$, which means the length of MEM is L, the MEM occurs at sequences $ID_1, \dots, ID_n$ with begins at $x_1, \dots, x_n$, found similar ~~string~~strings in sequences $ID_{n+1}, \dots, ID_f$ with ~~starts~~start at $x_{n+1}, \dots, x_f$ with length $y_{n+1} - x_{n+1}, \dots, y_f - x_f$. The number of strings contains in $\boldsymbol{MX}$ is $|\boldsymbol{MX}| = f$.

Definition 4: The area a of MEM with similar fragment ~~*M'*~~$\boldsymbol{MX}$ is defined as formula (3):

$$a = \max_{c} \left(\sum_{i}^{IDs} (y_i - x_i - |x_i - c|) \right) \tag{3}$$

where IDs means sequence identifiers in this MEM, IDs is the set contains all sequence IDs in this MEM, c means the center place of every sequence in MEM. The calculation of center c is same as formula (2).

Align by sorted MEMs

In this section, we use sorted MEMs to make alignment. It's worth noting that, MEMs are sorted by area, ~~the MEM~~ with the ~~highest~~MEM having the largest area placed first in ~~all MEMs is~~ the ~~first item in MEMs~~ array. For every MEM, we ~~obey the advice of MEM,~~ follow its guidance to

divide ~~determined~~the corresponding sequences into three parts: left block, right block and down block (details shown in Figure 3~~ (a)).~~). For ~~every sequence~~sequences not included in the MEM, we use the SSW library [38] to find similar ~~fragment. For sequences in~~ fragments and incorporate these fragments into the MEM, resulting in a new MEM referred to as "MEM with similar ~~fragment, we divide them as~~ fragments". Sequences in the "MEM with similar fragments" are divided into left and right blocks~~. As for~~, while sequences ~~which~~ not included in the "MEM with similar ~~fragment, we put them into~~fragments" are placed in the down block. ~~Algorithm 2 shows the~~ The core of the divide-and-conquer ~~method~~framework for aligning sequences ~~by~~using sorted MEMs is shown in Algorithm S2, and the illustration of Algorithm S2 is shown in Figure 3~~:~~.

~~Algorithm 2: Align by determined block, divide other blocks, or call final alignment.~~

~~Input: Multiple sequences $S_1, \dots, S_n$, interval for these sequences $[B_1, E_1), \dots, [B_n, E_n)$, determined MEM block $\boldsymbol{MX}$ with $|\boldsymbol{MX}| = m\ (m \le n)$, other MEMs $\boldsymbol{MX_1}, \boldsymbol{MX_2}, \dots, \boldsymbol{MX_M}$~~

~~Output: Aligned sequences, which aligned intervals $[B_1, E_1), \dots, [B_n, E_n)$ for corresponding sequences $S_1, \dots, S_n$~~

~~Sub_block_align($S_1, \dots, S_n, B_1, \dots, B_n, E_1, \dots, E_n, b, \boldsymbol{MX_1}, \dots \boldsymbol{MX_b}$) {~~

~~if(b == 0) call_alignment($S_1, \dots, S_n, B_1, \dots, B_n, E_1, \dots, E_n$); // call alignment method~~

~~else {~~

~~Sort($MX_1, \dots MX_b$); // sort the blocks by area~~

~~Align($S_1, \dots, S_n, B_1, \dots, B_n, E_1, \dots, E_n, \boldsymbol{MX_1}, \dots \boldsymbol{MX_b}$); }~~

~~}~~

~~Align($S_1, \dots, S_n, B_1, \dots, B_n, E_1, \dots, E_n, \boldsymbol{MX}, \boldsymbol{MX_1}, \boldsymbol{MX_2}, \dots, \boldsymbol{MX_M}$) {~~

~~// Part 1: use SSW to find sequences~~

```
// assume sequences m+1, ..., n has no information in MX, findsame function returns MEM with similar fragment, found the similar part for k sequences
MX'=findsame(MX, [S_{m+1}, ..., S_n], [B_m, E_m), ..., [B_n, E_n))
// Align MEM block with found similar blocks [L_{m+1}, R_{m+1}), ..., [L_{m+k}, R_{m+k})
Align_block([MX, [L_{m+1}, R_{m+1}), ..., [L_{m+k}, R_{m+k})]);
// Part 2: divide other blocks into left blocks, right blocks or down blocks
for(i=0; i<M; ++i) {
  for(interval in MX_i) {
    if(ID(interval) in Block_list) {
      if (interval both in left and right) interval.split_and_append(this_left, this_right);
      else if(interval in left) this_left.append(interval);
      else this_right.append(interval);
      }
    else this_down.append(interval);
  }
  // check the size of this block parted blocks, if size < 2, drop it
  if(this_left.size>=2) left_blocks.append(this_left);
  if(this_right.size>=2) right_blocks.append(this_right);
  if(this_down.size>=2) down_blocks.append(this_down);
}
// Part 3: call sub-alignment process
Sub_block_align(S_1, ..., S_{m+k}, B_1, ..., B_{m+k}, L_1, ..., L_{m+k}, left_blocks.size, left_blocks);
```

~~Sub_block_align($S_1, \dots, S_{m+k}, R_1, \dots, R_{m+k}, E_1, \dots, E_{m+k}$, right_blocks.size, right_blocks);~~

~~if ($m + k \neq n$) {~~

~~Sub_block_align($S_{m+k+1}, \dots, S_n, B_{m+k+1}, \dots, B_n, E_{m+k+1}, \dots, E_n$, down_blocks.size,~~

~~down_blocks);~~

~~ProfileProfileAlign([$S_1, \dots, S_{m+k}, B_1, \dots, B_{m+k}, E_1, \dots, E_{m+k}$], [$S_{m+k+1}, \dots, S_n, B_{m+k+1}, \dots,$ $B_n, E_{m+k+1}, \dots, E_n$]); // Profile-profile alignment of two parts~~

~~}~~

~~}~~

As shown in Algorithm ~~2~~S2, the alignment procedure can be summarized as follows: we sort MEM blocks according to their area, then call the main divide-and-conquer function for alignment based on MEM blocks. The internal logic of divide-and-conquer function can be concluded as follows: Firstly, the MEM may not contain all input sequences, therefore, we use the SSW algorithm to identify the sequences not covered by the MEM. After identifying these sequences, we merge the MEM with the corresponding intervals found by SSW. This combined block is treated as a MEM block with fragment parts. Using the MEM block with fragment parts, we divide the remaining sequences into three subproblems: align the left part, the right part and the down part. For each MEM, we determine the corresponding appearance in each sequence. The divide-and-conquer function is recursively called to align the subproblems. It is important to note that, if the down part is present, we must align the sequences in the down part with those in the MEM that contain the fragments, each of which has been aligned previously. To accelerate the divide-and-conquer procedure, we adopt a parallelization strategy similar to that used in FORAlign [40], using the fork-join model with work stealing to enhance computational efficiency.

Profile-profile Alignment and Fragment Alignment based on Existing Approaches

In previous sections, we introduced the alignment based on MEMs. In Figure 1 ~~(b),~~Step 2, after processing MEM, we call the sub alignment methods to make alignment. The sub alignment method is same as the father alignment method, except for the condition of no MEMs. If any part has no MEMs, we call the fragment alignment method. We determine the fragment alignment method like MAFFT FFT-NS-1 [14], WMSA [17] or abPOA [33]. Like the sub alignment, we also compute profiles alignment by these methods. Since these methods cannot make profile-profile directly, we modified them to support profiles alignment. In particular, we made a list for representing sequences to profiles, and call profile-profile alignment to make the real alignment.

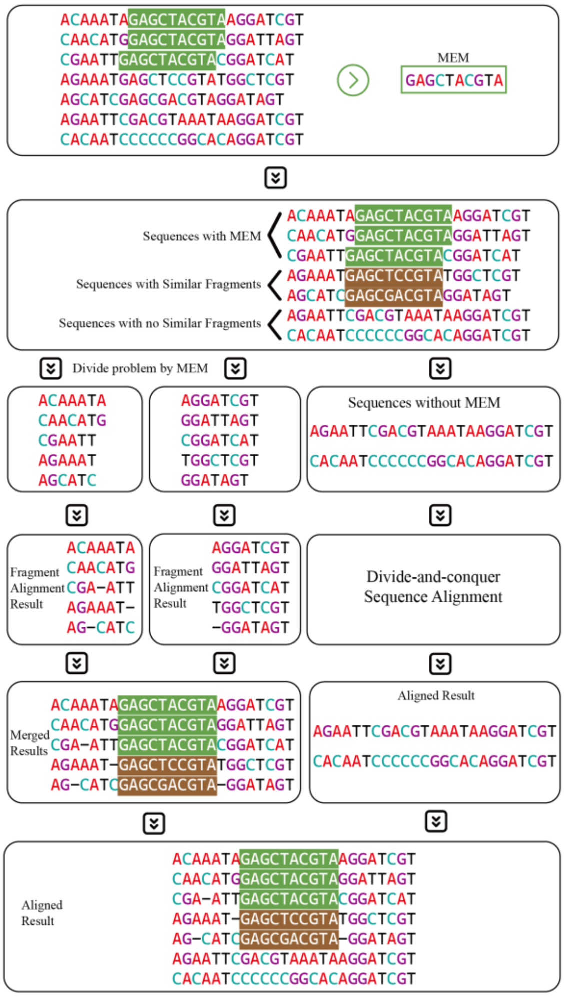
MEM
GAGCTACGTA
Sequences with MEM
Sequences with Similar Fragments
Sequences with no Similar Fragments
Divide problem by MEM
Sequences without MEM
Fragment Alignment Result
Divide-and-conquer Sequence Alignment
Merged Results
Aligned Result
Aligned Result

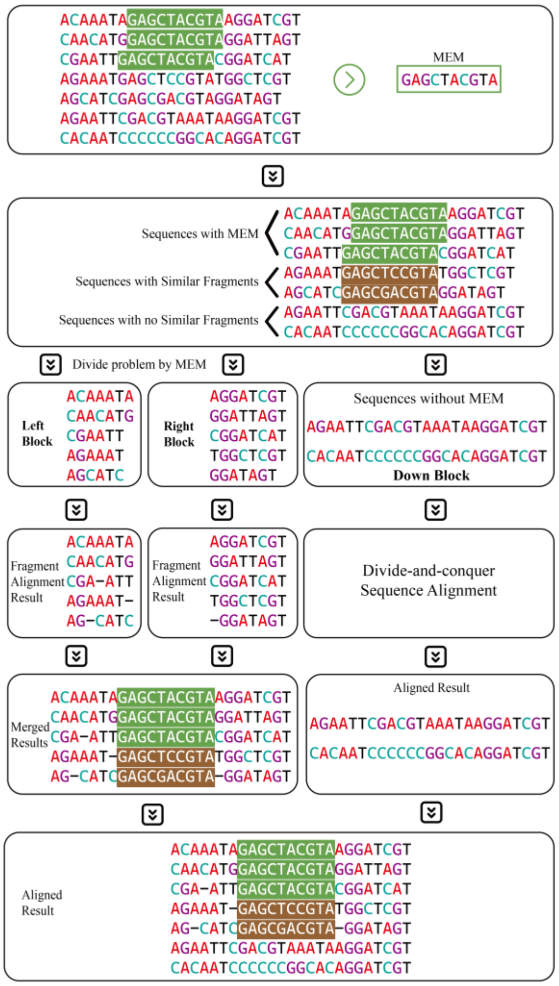

Figure 3: Details for aligning sequences by sorted MEMs. We found MEM “GAGCTACGTA” in these sequences, the MEM occurs on the first, second and third sequences (colored area). After that, we call SSW function, found the similar parts “GAGCTCCGTA” and “GAGCGACTA”. As a conclusion, the MEM with similar fragment is on the 1st~5th strings. After generating MEM with similar fragment, the left and right blocks is generated, and the down block is also generated. After finding similar sequence segment, we divide sequences into four parts: left block, right block, down block and MEM with similar

fragment. The left, right and down block are aligned by sub alignment function (Sub_block_align function in Algorithm 2). We need to wait left and right block alignment to generate the sequence alignment result for the sequences in MEM with similar fragment. After generating sequence result, we need to wait down block to make alignment with sequences in MEM and sequences out MEM to generate the final result

Time and space analysis for the whole algorithm

deMEM can be divided on three modules: (A) Identifying MEMs and clustering sequences based on the de Bruijn graph; (B) Aligning sequences within each cluster using the divide-and-conquer ~~method~~framework based on MEMs to generate profiles; (C) Performing profile-profile alignment between different clusters. The time and space complexity analysis of the algorithm is presented below:

(A) Find MEMs and make clusters based on de Bruijn graph: assume we have n sequences, the length of sequences is S. We use BWT enhanced SplitMEM to find MEMs, the time complexity of this algorithm is $O\big((n+S)\log\sigma\big) \approx O(n+S)$, the space complexity is $O(n+S)$ to store all nodes in de Bruijn graph; make clusters based on MEMs, we need to use disjoint-set for making clusters, the time complexity of clustering is $O((n+S)\alpha(n,n)) \approx O(n+S)$. In conclusion, the time and space complexity of step (A) is $O(n+S)$;

(B) Divide-and-conquer ~~method~~framework based on MEMs: assume we have n sequences with the minimum sequence length m, we can infer that the graph length in step (A) has

maximum $O(nm)$ nodes. For every MEM, calculate the area needs $\mathrm{O}(n\log n)$ time. As for graph has at most $O(nm)$ nodes, the time complexity of sort is $O(nm\log(nm))$. Next, we align blocks by sorted MEMs. For every MEM, assume this size of MEM is $A\times y$, and the size of align region is $n\times m$, which A is the number of sequences, y is the length of MEM, n is the number of sequences, and m is the length of all sequences. Time complexity of region alignment is $T(n,m)=T(A,l)+T(A,r)+T(n-A,m)+S(n,m)$. We discuss the result of $S(n,m)$: firstly, we try to use SSW to find similarity part, we need to find $n-A$ sequences, the time complexity of SSW is $\mathrm{O}((n-A)ky)$ (use K-band) or $\mathrm{O}\big((n-A)my\big)$ (no K-band); then, we divide other MEMs into three parts, which need $O(nm)$ time; next, we wait the results of sub-function, merge and refine the results, which need $O(A)$; lastly, the border condition of the whole alignment is no MEMs in part, which we call MAFFT, WMSA or abPOA to make alignment, the time complexity of the whole algorithm may be influenced by determined algorithm: if we use WMSA with K-band, the time complexity of align is $O(xyk)$; otherwise, the time complexity of align is $O(xy^2)$; in conclusion, $S(n,m)=O(knm)$ (with K-band) ~ $O(nm^2)$ (without K-band). The analysis for $T(n,m)$ is similar with [41]. As for a result, $T(n,m)=O(knm)$ (with K-band) ~ $O(nm^2)$ (without K-band);

(C) Profile-profile alignment between different clusters: assume we generate C clusters in step (A), the maximum length of profile is P. the profile-profile alignment step requires a progressive profile-profile alignment process. Since $C\ll P$, so the time complexity of this step is $\mathrm{O}(C^3+C^2P^2)\approx\mathrm{O}(C^2P^2)$, and the space complexity is $\mathrm{O}(C^2P^2)$.

As a result, the time and space complexity of the whole deMEM algorithm is

$O(Cnm^2 + C^2P^2)$, which C represents the number of clusters, n is the number of sequences, m is the length of the longest sequence, P is the length of the aligned profiles.

Datasets and measurement

To evaluate the alignment results of our proposed method, we developed a software package called deMEM. In this section, we first introduce the datasets used to compare deMEM with other methods, followed by a description of the test methods. Finally, we outline the evaluation metrics and computational resources utilized in this experiment.

Experimental Datasets: Because deMEM ~~breaks~~divides sequences into multiple parts, ~~we require~~ multiple conditions ~~for showing~~are required to demonstrate the advantages ~~for~~of our ~~methods~~method. To ~~broadly test our methods, we test the~~comprehensively evaluate its performance, we conducted experiments on both real ~~datasets~~ and simulated ~~tests~~datasets. Thus, we choose the following datasets, shown in Table 1 and Table 2:

Table 1 Description of the datasets tested in deMEM (Real data)

Dataset name	Source of dataset	Sequences	Average sequence length	Length distribution	References
mt1x	Mt genomes	672	16568.3	16555~16578	[11, 17, 26, 42]
mt20x		13440			
Complete156	SARS-CoV-2	156	29855.1	29409~29927	[17, 42]
Mix1t		1024	27556.8	64~29981	
MPoX	Monkey Pox	1739	197084.9	183230~210918	[42, 43]

	virus				
Variola	Variola virus	4	186374.3	186064~186677	[26]
Mycoplasma	*Mycoplasma bovis*		579708.8	579504~579977	
Streptococcus	*Streptococcus pneumonia*		2160522	2111882~ 2184682	
Ecoli	*Escherichia coli*		4633445.8	4578159~ 4686137	
Nerisseria	*Nerisseria meningitidis*	5	2190087.6	2145295~ 2272360	First collected
23sr	*Mycobacterium* 23S rRNA	641	3113.1	1909~3485	[11]

Table 2 Description of the datasets tested in deMEM (Simulated data)

Test Name	Sequences	Average length	Length distribution	Test cases	Reference
RNA-255	255	1527	1518~1542	10	[44]
RNA-511	511	1528	1518~1542		
RNA-1023	1023	1527	1517~1542		
RNA-2047	2047	1527	1517~1542		
RNA-4095	4095	1527	1516~1542		
mt-similarity	112	15860±115	15719±220~ 15992±12	9	[11]

SARS-CoV-2-similarity	112	29675±118	29404±316 ~ 30000±0		

In Table 1, we newly collected the *Nerisseria meningitidis* sequences to show the quality for our methods.

Experimental methods: We compared our method with FAME [26] and FMAlign2 [28], both of which use chain-based strategies. As described before, we employed MAFFT FFT-NS-1 [14], abPOA [33] and WMSA [17] to calculate sub alignments. Our experiments can be divided into two main parts: (a) evaluating the improvements by FAME, FMAlign2 and our method for sub alignment strategies like MAFFT FFT-NS-1; (b) compare the different sub alignment strategies like MAFFT FFT-NS-1, abPOA and WMSA, under two approaches: treating all sequences as a cluster (methods *-H in result tables) or grouping sequences into multiple clusters (methods *-L in result tables). ~~For consistency, all alignment methods were tested using~~It is worth noting that if deMEM does not find any MEMs, the program falls back to the original alignment method. For consistency, since different alignment methods employ distinct scoring systems (e.g., abPOA uses two-piece gap affine penalty scoring system, while WMSA and MAFFT use simple gap affine penalty scoring system), all alignment methods were evaluated using their default parameters.

Experimental Metrics: We measure the real data alignment results by SP score introduced in [27], with match score=0, mismatch score=-1 and gap score=-2. A lower SP score indicates fewer inserted gaps, reflecting improved alignment consistency and overall quality. For simulated datasets, we use Q and TC score designed in MUSCLE [15] for measuring the results for all methods.

Computational Resources: Our experiment is tested on a workstation with 1TB main memory, an Intel(R) Xeon(R) Gold 6230 CPU processor with 80 cores with 2.10GHz CPU frequency under the

Ubuntu 20.04 operating system.

Results

Experimental Results on real datasets

In this section, we present the results of real datasets. A summary of results is provided in ~~Table 3 and Figure 4.~~Figure 4, which the detailed results are shown in Table S1. From ~~Table 3~~Table S1 and Figure 4, we observe that our method improves the SP Scores in real datasets, particularly in extremely large datasets. Compared to seed-and-extension MSA methods, our method achieved higher SP scores more than FMAlign2 and FAME. A key advantage of deMEM is its ability to ~~ability to~~ integrate both vertical and horizontal sequence information, leading to more comprehensive sequence alignments. For extremely large and long sequences, such as those in the MPoX dataset, although FAME ~~demonstrated fast~~achieved faster alignment with ~~low~~lower memory ~~usage but~~consumption, it produced lower-quality ~~alignments~~results compared to deMEM. Specifically, deMEM improved alignment quality by approximately 50.3% over FAME and 32.0% over FMAlign2 on the MPoX dataset.

Comparison between seed-and-extension MSA methods

To evaluate the enhancement for SP scores between FMAlign2, FAME and our method, we independently ran the MAFFT FFT-NS-1 method and calculated the SP score using the aforementioned methods. The results are presented in Table 3. Due to the limitations of MAFFT FFT-NS-1, it can only directly align sequences in the cases listed in Table 3. It's worth noting that, compared to FAME and FMAlign2, our method enhanced the performance of FFT-NS-1 when

aligning large sequences. Specifically, compared to FAME, deMEM can align extremely long sequences, without any decrease in SP scores and demonstrates improved robustness, consistently aligning sequences with stability. Compared to FMAlign2, our method performs better on mt sequences. For extremely large and long sequences, such as *Mycoplasma bovis* sequences, deMEM offers modest improvements in alignment quality but uses significantly less memory.

Table 3 Results in real datasets. "Block size" in this table means the maximum SP score aligned by the determined MEM block size. “ ” in SP means the corresponding method can not make alignment. We tested FAME as the default argument

Method Name	mt1x				mt20x				Complete156			
	Block Size	SP	Time /s	Memory /MB	Block Size	SP	Time /s	Memory /MB	Block Size	SP	Time /s	Memory /MB
FAME	-	-212.1	9.0	119.2	-	-211.9	**35.4**	2109.7	7	-403.3	4.5	99.1
FMAlign2	20	-150.7	**5.9**	314.3	500	-152.3	1202.1	4890.1	1000	-261.6	**1.2**	105.8
deMEM-abPOA-L	10	-154.6	88.0	1465.5	20000	-158.2	882.0	3827.0	200	-299.2	12.6	622.1
deMEM-abPOA-H	5000	-156.1	20.4	354.3	5000	-154.1	823.8	1065.3	800	-338.2	3.7	73.3
deMEM-FFTNS1-L	50	-153.6	215.0	2402.5	15000	-152.2	270289.0	128959.8	20000	-273.8	32.9	326.9
deMEM-FFTNS1-H	100	**-148.2**	67.1	337.3	100	**-148.2**	12810.0	3683.1	7000	-261.8	7.7	**42.2**
deMEM-WMSA-L	5000	-149.5	432.1	1068.9	50	-149.5	1133.8	50371.2	20000	**-244.5**	31.7	352.7
deMEM-WMSA-H	5000	-149.1	27.6	**56.2**	5000	-149.0	581.2	**1065.3**	12000	-244.8	8.7	49.0
Method Name	Mix1t				23sr				*Variola virus*			
	Block Size	SP	Time /s	Memory /MB	Block Size	SP	Time /s	Memory /MB	Block Size	SP	Time /s	Memory /MB
FAME	-	-	-	-	-	-	-	-	-	-2913.3	8.6	37.7
FMAlign2	2000	-46860.8	1654.8	2290.6	1000	-2367.0	12.8	178.5	100	-2700.3	3.4	20.6
deMEM-abPOA-L	50	-14490.2	1483.1	23572.9	1200	-4348.5	61.9	394.5	800	-2363.7	4.1	254.3
deMEM-abPOA-H	100	-51592.0	3094.6	34026.5	1200	-3377.5	2.9	**44.0**	1000	-2203.7	**1.9**	76.7
deMEM-FFTNS1-L	100	-18886.8	11462.0	68385.3	2000	-2367.0	10.5	526.2	5000	-2696.0	39.5	67.3
deMEM-FFTNS1-H	100	-18886.8	10907.0	67101.7	2000	-2367.0	11.1	553.1	800	-2703.2	77.2	7.8
deMEM-WMSA-L	100	**-13036.7**	79.0	4677.1	20	**-1742.6**	10.0	403.5	20000	**-2164.0**	20.3	78.9
deMEM-WMSA-H	100	**-13036.7**	**75.4**	**4298.9**	2000	-2408.5	**2.6**	178.3	500	-2712.8	7.5	**7.4**
Method Name	MPoX				*Mycoplasma bovis*				*Streptococcus pneumonia*			
	Block Size	SP	Time /s	Memory /MB	Block Size	SP	Time /s	Memory /MB	Block Size	SP	Time /s	Memory /MB
FAME	-	-206051.9	**36.8**	**1770.2**	-	-	-	-	-	-	-	-
FMAlign2	10	-150564.9	510629.0	9512.8	500	**-7127.8**	**2.4**	60.2	2000	**-889430.7**	2748.4	**217.5**

deMEM-abPOA-L	-	Out of Memory			500	-8062.0	29.8	2102.9	-	Out of Memory		
deMEM-abPOA-H	10000	-387056.7	459.3	4217.9	5000	-7872.8	60.7	15546.7	1000	-2660563.2	**319.8**	11752.0
deMEM-FFTNS1-L	12000	-182553.8	52783.0	48684.3	10000	-7149.0	339.2	718.1	1000	-1225436.5	1185.8	520.5
deMEM-FFTNS1-H	10000	-398205.1	1571.5	8288.8	5000	-7130.3	199.0	**52.9**	10000	-1826520.3	27839.0	1671.0
deMEM-WMSA-L	12000	**-102311.0**	41257.0	99238.5	5000	-7342.7	3015.9	319.0	1000	-901459.0	79250.0	150746.9
deMEM-WMSA-H	10000	-436952.5	1642.2	4440.4	5000	-7583.5	27.0	96.5	10000	-1364741.2	15354.0	127781.6

Method Name	*Escherichia coli*				*Nerisseria meningitidis*			
	Block Size	SP	Time /s	Memory /MB	Block Size	SP	Time /s	Memory /MB
FAME	-	-	-	-	-	-	-	-
FMAlign2	100	-2894827	**727**	12773	2000	**-2980447**	166637	2767
deMEM-abPOA-L	-	Out of Memory			-	Out of Memory		
deMEM-abPOA-H	500	-16962803	358	259807	1000	-8853709	**515**	105222
deMEM-FFTNS1-L	2000	-1663531	4757	**1487**	2000	-3281288	60664	1347
deMEM-FFTNS1-H	500	-16868812	1555	2040	5000	-4023464	39066	**678**
deMEM-WMSA-L	2000	**-1558240**	86174	508726	-	Out of Memory		
deMEM-WMSA-H	500	-16744612	2833	156754	2000	-6102398	6624	210283

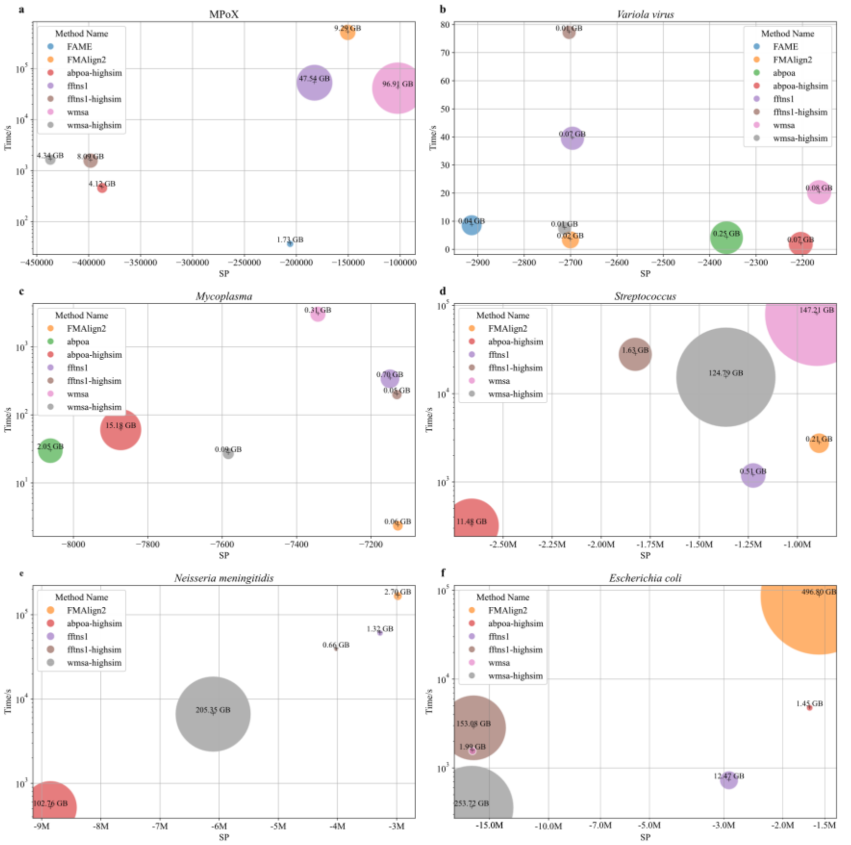
a
MPoX
Method Name
FAME
FMAlign2
abpoa-highsim
fftns1
fftns1-highsim
wmsa
wmsa-highsim
9.29 GB
47.54 GB
96.91 GB
4.34 GB
8.09 GB
4.12 GB
1.73 GB
Time/s
SP
b
Variola virus
Method Name
FAME
FMAlign2
abpoa
abpoa-highsim
fftns1
fftns1-highsim
wmsa
wmsa-highsim
0.01 GB
0.07 GB
0.08 GB
0.04 GB
0.01 GB
0.02 GB
0.25 GB
0.07 GB
Time/s
SP
c
Mycoplasma
Method Name
FMAlign2
abpoa
abpoa-highsim
fftns1
fftns1-highsim
wmsa
wmsa-highsim
0.31 GB
0.70 GB
0.05 GB
15.18 GB
2.05 GB
0.09 GB
0.06 GB
Time/s
SP
d
Streptococcus
Method Name
FMAlign2
abpoa-highsim
fftns1
fftns1-highsim
wmsa
wmsa-highsim
147.21 GB
1.63 GB
124.79 GB
0.21 GB
0.51 GB
11.48 GB
Time/s
SP
e
Neisseria meningitidis
Method Name
FMAlign2
abpoa-highsim
fftns1
fftns1-highsim
wmsa-highsim
2.70 GB
1.32 GB
0.66 GB
205.35 GB
102.76 GB
Time/s
SP
f
Escherichia coli
Method Name
FMAlign2
abpoa-highsim
fftns1
fftns1-highsim
wmsa
wmsa-highsim
496.80 GB
1.45 GB
153.08 GB
1.99 GB
12.47 GB
253.72 GB
Time/s
SP

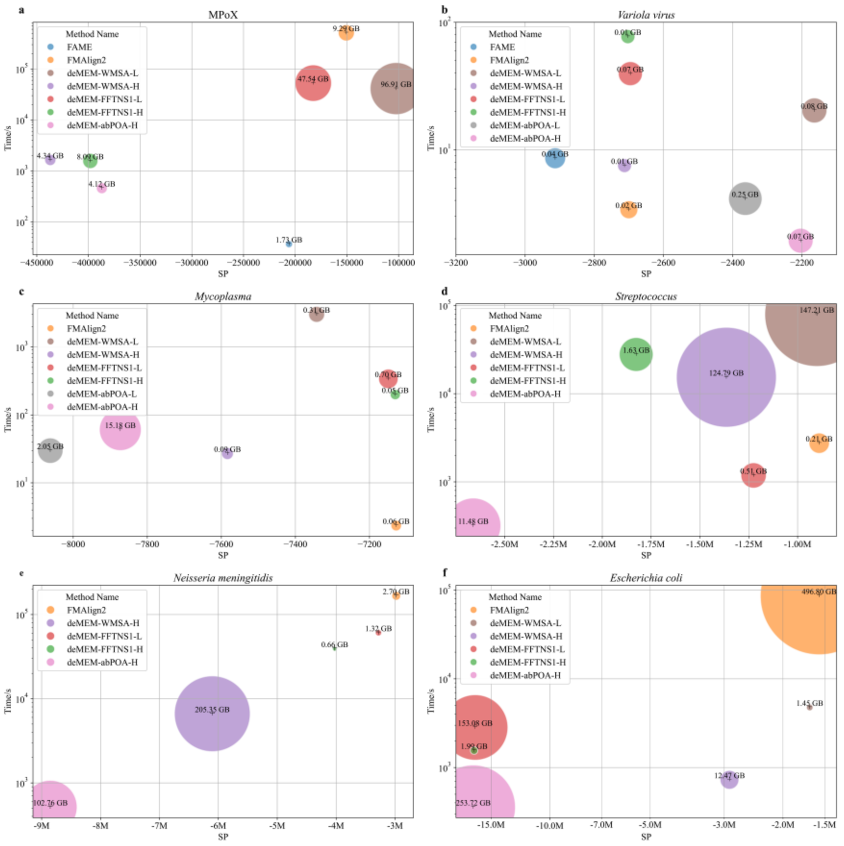

Figure 4. Illustration of real dataset results in different methods. The area of circle is the memory usage of the determined method. This figure shows the best SP values between same method, excluded the out of memory methods. Lower absolute SP scores indicate better alignment quality

Table 3 Result difference between seed-and-extension methods and MAFFT FFT-NS-1. "Block Size" in this table means the maximum SP score aligned by the determined MEM block size. ΔSP means the difference value between the determined method with MAFFT FFT-NS-1

Method Name	mt1x	mt20x	*Mycoplasma bovis*

	Block Size	ΔSP	Block Size	ΔSP	Block Size	ΔSP
FAME	-	-52.3	-	-52.2	-	-
FMAlign2	20	9.1	500	7.4	500	**21.2**
deMEM-FFTNS1-L	50	6.2	15000	7.6	10000	0.0
deMEM-FFTNS1-H	100	**11.6**	100	**11.6**	5000	18.7

Comparison between sub alignment methods in deMEM

deMEM supports various sub-alignment methods, including abPOA, FFT-NS-1 and WMSA. The quality and speed of alignment are influenced by the choice of sub-alignment method. Each method has its own advantages: abPOA can align smaller sequences quickly with minimal memory usage, but it is less suitable for long and large sequences; FFT-NS-1 is optimal for aligning highly similar sequences; WMSA fits in aligning huge and long sequences with low similarity. As shown in Figure 4, our method effectively aligns extremely large datasets, demonstrating its scalability.

To further evaluate the impact between different methods, we tested the mt1x and mt20x datasets use these sub-alignment methods, as shown in Figure 5. The results indicate that sub-alignment methods primarily influence memory and runtime efficiency. Notably, abPOA exhibited slow alignment speeds because it was executed on a single-threaded process, significantly affecting performance.

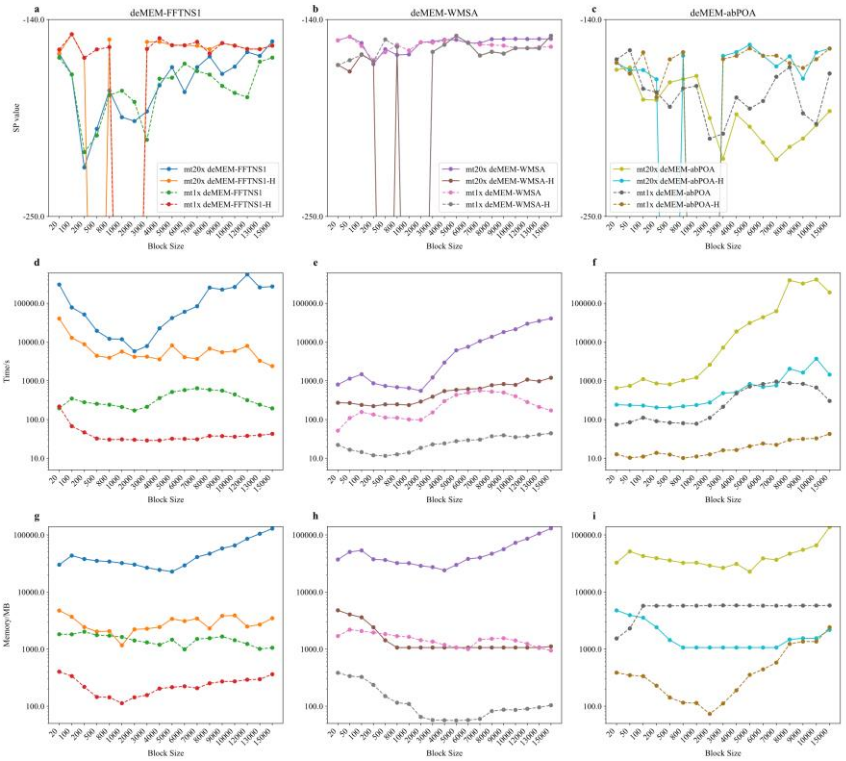

Figure 5 SP Scores, time and memory change with block size in mt1x and mt20x datasets.

Experimental Results on simulated datasets

Simulated datasets provide real alignment results, allowing for a direct comparison between

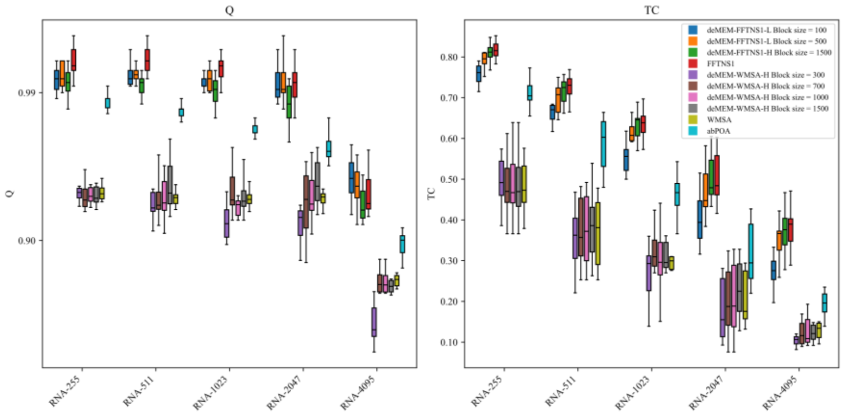

~~Figure 6 Q and TC Score in RNA simulated tests. Only show the enhanced deMEM methods.~~

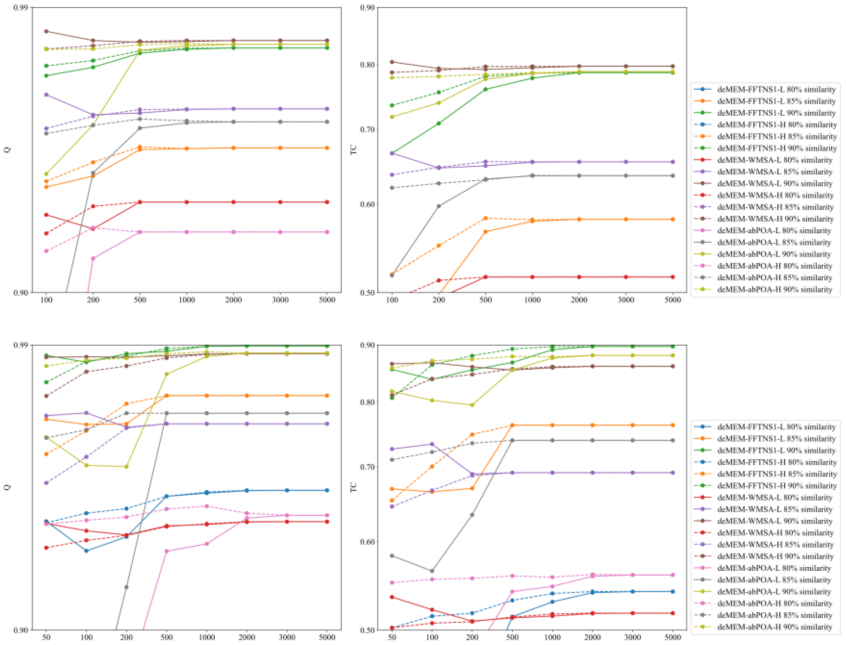

~~Figure 7 Q and TC score in two similarity datasets. Only shown Q and TC score in 80%-90% similarity.~~

~~Upper: mt-similarity dataset; lower: SARS-CoV-2 similarity dataset~~

alignment results and actual alignments. We evaluated our method on two simulation datasets, as shown in Figure 6 and Figure 7. Figure 6Figure 6 and Figure 7. Figure 6 presents the Q and TC score improvements between sub alignment methods and raw alignment methods. Our method achieved slightly higher quality alignment result compared to WMSA raw methods in simulated RNA test cases. Figure 7Figure 7 highlights the impact of small MEM blocks on alignment accuracy. The results indicate that incorporating small MEM blocks enhances both Q-_score and TC-_score, leading to improved overall alignment quality.

Formatted: Indent: First line: 2 ch

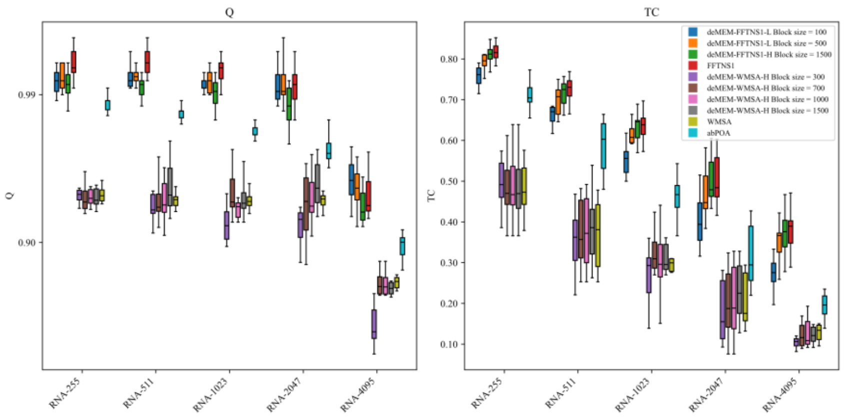

Figure 6 Q and TC Score in RNA simulated tests. Only show the enhanced deMEM methods.

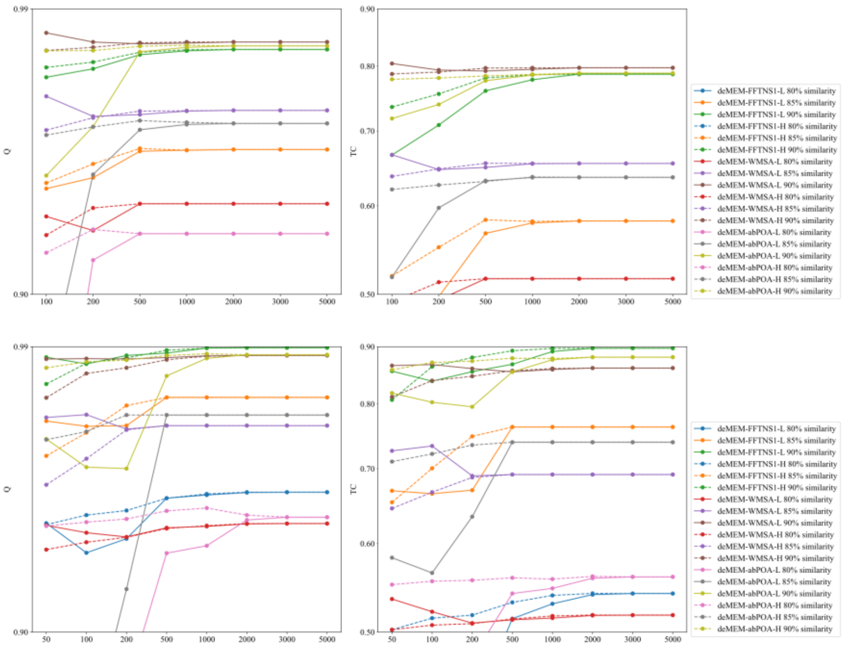

Figure 7 Q and TC score in two similarity datasets. Only shown Q and TC score in 80%~90% similarity. Upper: mt-similarity dataset; lower: SARS-CoV-2 similarity dataset

Conclusion

In this paper, we introduced deMEM, a novel framework for MSA, supports horizontal and vertical problem decomposition based on MEMs. The method follows a three-step approach: First, deMEM identifies MEMs and clusters with all input sequences using de Bruijn graph. Next, sequences in clusters are aligned into profiles by divide-and-conquer ~~methods~~framework based on MEMs. Lastly, profiles are progressively aligned to obtain the final MSA. The core of deMEM is the divide-and-conquer framework, where MEMs are used to split the alignment problem into smaller subproblems in both vertical and horizonal conditions. Experiments showed that deMEM

outperforms its counterpart tools at large scale data, such as a thousand monkeypox genomes., which improved alignment quality by approximately 50.3% over FAME and 32.0% over FMAlign2 on the MPoX dataset. Additionally, experiments on simulated data indicate that deMEM achieves improved alignment accuracy with slight enhancements. As a future work, we plan to improve deMEM method to optimize the space usage to support extremely long and large-scale sequences alignment. Moreover, we will focus on protein sequence alignment in the future to extend the application of our method.

The deMEM package is freely available at https://github.com/malabz/deMEM. It has been tested on the Linux and Windows. This package is also available on conda.

Declaration of competing interest

The authors declare that they have no known competing financial interests or personal relationships that could have appeared to influence the work reported in this paper.

Data availability

The datasets and are publicly available at https://doi.org/10.5281/zenodo.14989520.

Availability of source code and requirements

- Project name: deMEM
- Project home page: https://github.com/malabz/deMEM
- Operating system(s): Linux (Recommended) & Windows
- Programming language: C++
- Other requirements: Anaconda (Recommended)
- License: MIT
- Any restrictions to use by non-academics: License needed

Funding

This work was supported by the National Natural Science Foundation of China [grant number 62472344, 62452107, 62072353 and 62272065]; and Xidian University Specially Funded Project for Interdisciplinary Exploration (No. TZJH2024027).

Acknowledgments

We acknowledge the help from the other group members: Yixiao Zhai, Tong Zhou, and Qinzhong Tian for providing critical opinions during the preparation.

References

1. Wang GH, Liu YL, Zhu DX, Klau GW and Feng WX. Bioinformatics Methods and Biological Interpretation for Next-Generation Sequencing Data. Biomed Res Int. 2015;2015 doi:10.1155/2015/690873.
2. Yin C, Wang R, Qiao J, Shi H, Duan H, Jiang X, et al. NanoCon: contrastive learning-based deep hybrid network for nanopore methylation detection. Bioinformatics. 2024;40 2:btae046.
3. Chitsaz H, Yee-Greenbaum JL, Tesler G, Lombardo MJ, Dupont CL, Badger JH, et al. Efficient de novo assembly of single-cell bacterial genomes from short-read data sets. Nat Biotechnol. 2011;29 10:915-21. doi:10.1038/nbt.1966.
4. Sohn JI and Nam JW. The present and future of de novo whole-genome assembly. Brief Bioinform. 2018;19 1:23-40. doi:10.1093/bib/bbw096.
5. Muyas F, Sauer CM, Valle-Inclan JE, Li R, Rahbari R, Mitchell TJ, et al. De novo detection of somatic mutations in high-throughput single-cell profiling data sets. Nat Biotechnol. 2024;42 5:758-67. doi:10.1038/s41587-023-01863-z.
6. Tian Q, Zhang P, Zhai Y, Wang Y, Zou Q and Stairs C. Application and Comparison of Machine Learning and Database-Based Methods in Taxonomic Classification of High-Throughput Sequencing Data. Genome Biology and Evolution. 2024;16 5 doi:10.1093/gbe/evae102.
7. Wang L, Ding Y, Tiwari P, Xu J, Lu W, Muhammad K, et al. A deep multiple kernel learning-based higher-order fuzzy inference system for identifying DNA N4-methylcytosine sites. Information Sciences. 2023;630:40-52. doi:10.1016/j.ins.2023.01.149.
8. Chao J, Tang F and Xu L. Developments in Algorithms for Sequence Alignment: A Review. Biomolecules. 2022;12 4 doi:10.3390/biom12040546.

Formatted: Line spacing: single

9. Zou Q, Hu Q, Guo M and Wang G. HAlign: Fast multiple similar DNA/RNA sequence alignment based on the centre star strategy. Bioinformatics. 2015;31 15:2475-81. doi:10.1093/bioinformatics/btv177.
10. Wan S and Zou Q. HAlign-II: efficient ultra-large multiple sequence alignment and phylogenetic tree reconstruction with distributed and parallel computing. Algorithms Mol Biol. 2017;12:25. doi:10.1186/s13015-017-0116-x.
11. Tang F, Chao J, Wei Y, Yang F, Zhai Y, Xu L, et al. HAlign 3: Fast Multiple Alignment of Ultra-Large Numbers of Similar DNA/RNA Sequences. Mol Biol Evol. 2022;39 8 doi:10.1093/molbev/msac166.
12. Zhou T, Zhang P, Zou Q and Han W. HAlign 4: a new strategy for rapidly aligning millions of sequences. Bioinformatics. 2024;40 12 doi:10.1093/bioinformatics/btae718.
13. Higgins DG and Sharp PM. CLUSTAL: a package for performing multiple sequence alignment on a microcomputer. Gene. 1988;73 1:237-44. doi:10.1016/0378-1119(88)90330-7.
14. Katoh K, Misawa K, Kuma K and Miyata T. MAFFT: a novel method for rapid multiple sequence alignment based on fast Fourier transform. Nucleic Acids Res. 2002;30 14:3059-66. doi:DOI 10.1093/nar/gkf436.
15. Edgar RC. MUSCLE: multiple sequence alignment with high accuracy and high throughput. Nucleic Acids Res. 2004;32 5:1792-7. doi:10.1093/nar/gkh340.
16. Deorowicz S, Debudaj-Grabysz A and Gudys A. FAMSA: Fast and accurate multiple sequence alignment of huge protein families. Sci Rep. 2016;6:33964. doi:10.1038/srep33964.
17. Wei Y, Zou Q, Tang F and Yu L. WMSA: a novel method for multiple sequence alignment of DNA sequences. Bioinformatics. 2022;38 22:5019-25. doi:10.1093/bioinformatics/btac658.
18. Lyras DP and Metzler D. ReformAlign: improved multiple sequence alignments using a profile-based meta-alignment approach. BMC Bioinformatics. 2014;15 1:265. doi:10.1186/1471-2105-15-265.
19. Zhai Y, Chao J, Wang Y, Zhang P, Tang F and Zou Q. TPMA: A two pointers meta-alignment tool to ensemble different multiple nucleic acid sequence alignments. PLOS Computational Biology. 2024;20 4 doi:10.1371/journal.pcbi.1011988.
20. Zhai Y, Zhou T, Wei Y, Zou Q and Wang Y. ReAlign-N: an integrated realignment approach for multiple nucleic acid sequence alignment, combining global and local realignments. NAR Genomics and Bioinformatics. 2024;6 4 doi:10.1093/nargab/lqae170.
21. Liu Y, Shen X, Gong Y, Liu Y, Song B and Zeng X. Sequence Alignment/Map format: a comprehensive review of approaches and applications. Briefings in Bioinformatics. 2024;24 5:bbad320.
22. Qiao J, Jin J, Yu H and Wei L. Towards Retraining-free RNA Modification Prediction with Incremental Learning. Information Sciences. 2024:120105.
23. Li H and Liu B. BioSeq-Diabolo: Biological sequence similarity analysis using Diabolo. PLOS Computational Biology. 2023;19 6:e1011214.
24. Li H, Pang Y and Liu B. BioSeq-BLM: a platform for analyzing DNA, RNA, and protein sequences based on biological language models. Nucleic Acids Res. 2021;49

22:e129.
25. Li H. Minimap2: pairwise alignment for nucleotide sequences. Bioinformatics. 2018;34 18:3094-100. doi:10.1093/bioinformatics/bty191.
26. Naznooshsadat E, Elham P and Ali S-Z. FAME: fast and memory efficient multiple sequences alignment tool through compatible chain of roots. Bioinformatics. 2020;36 12:3662-8.
27. Liu H, Zou Q and Xu Y. A novel fast multiple nucleotide sequence alignment method based on FM-index. Brief Bioinform. 2022;23 1 doi:10.1093/bib/bbab519.
28. Zhang P, Liu H, Wei Y, Zhai Y, Tian Q and Zou Q. FMAlign2: a novel fast multiple nucleotide sequence alignment method for ultralong datasets. Bioinformatics. 2024;40 1 doi:10.1093/bioinformatics/btae014.
29. Zhang Y and Waterman MS. An Eulerian path approach to global multiple alignment for DNA sequences. J Comput Biol. 2003;10 6:803-19. doi:10.1089/106652703322756096.
30. Zhang Y and Waterman MS. An Eulerian path approach to local multiple alignment for DNA sequences. Proc Natl Acad Sci U S A. 2005;102 5:1285-90. doi:10.1073/pnas.0409240102.
31. De Bruijn NG. A combinatorial problem. Proceedings of the Section of Sciences of the Koninklijke Nederlandse Akademie van Wetenschappen te Amsterdam. 1946;49 7:758-64.
32. Lee C, Grasso C and Sharlow MF. Multiple sequence alignment using partial order graphs. Bioinformatics. 2002;18 3:452-64. doi:10.1093/bioinformatics/18.3.452.
33. Gao Y, Liu Y, Ma Y, Liu B, Wang Y and Xing Y. abPOA: an SIMD-based C library for fast partial order alignment using adaptive band. Bioinformatics. 2021;37 15:2209-11. doi:10.1093/bioinformatics/btaa963.
34. Liu B, Guo H, Brudno M and Wang Y. deBGA: read alignment with de Bruijn graph-based seed and extension. Bioinformatics. 2016;32 21:3224-32. doi:10.1093/bioinformatics/btw371.
35. Marcus S, Lee H and Schatz MC. SplitMEM: a graphical algorithm for pan-genome analysis with suffix skips. Bioinformatics. 2014;30 24:3476-83. doi:10.1093/bioinformatics/btu756.
36. Baier U, Beller T and Ohlebusch E. Graphical pan-genome analysis with compressed suffix trees and the Burrows-Wheeler transform. Bioinformatics. 2016;32 4:497-504. doi:10.1093/bioinformatics/btv603.
37. Tarjan RE. A class of algorithms which require nonlinear time to maintain disjoint sets. Journal of Computer and System Sciences. 1979;18 2:110-27. doi:10.1016/0022-0000(79)90042-4.
38. Zhao M, Lee WP, Garrison EP and Marth GT. SSW library: an SIMD Smith-Waterman C/C++ library for use in genomic applications. PLoS One. 2013;8 12:e82138. doi:10.1371/journal.pone.0082138.
39. Khan Z, Bloom JS, Kruglyak L and Singh M. A practical algorithm for finding maximal exact matches in large sequence datasets using sparse suffix arrays. Bioinformatics. 2009;25 13:1609-16. doi:10.1093/bioinformatics/btp275.
40. Wei Y, Zhou T, Zhai Y, Yu L and Zou Q. FORAlign: accelerating gap-affine DNA

pairwise sequence alignment using FOR-blocks based on Four Russians approach with linear space complexity. Brief Bioinform. 2025;26 1 doi:10.1093/bib/bbaf061.
41. Hirschberg DS. A linear space algorithm for computing maximal common subsequences. Communications of the ACM. 1975;18 6:341-3. doi:10.1145/360825.360861.
42. Kong X, Shen C and Tang J. CUK-Band: A CUDA-Based Multiple Genomic Sequence Alignment on GPU. In: Singapore, 2024, pp.84-95. Springer Nature Singapore.
43. Ma Y, Chen M, Bao Y and Song S. MPoxVR: A comprehensive genomic resource for monkeypox virus variant surveillance. The Innovation. 2022;3 5 doi:10.1016/j.xinn.2022.100296.
44. Chen J, Chao J, Liu H, Yang F, Zou Q and Tang F. WMSA 2: a multiple DNA/RNA sequence alignment tool implemented with accurate progressive mode and a fast win-win mode combining the center star and progressive strategies. Brief Bioinform. 2023;24 4 doi:10.1093/bib/bbad190.

Manuscript (Clean Version)
Click here to access/download;Manuscript;deMEM-20251103.docx

deMEM: a novel divide-and-conquer framework based on de Bruijn graph for scalable multiple sequence alignment

Yanming Wei[1,2], Zhaoyang Huang[1], Pinglu Zhang[2,3], Yizheng Wang[2,3], Yan Li[4], Liang Yu[1,*, ¶], Quan Zou[2,3,*, ¶]

[1] School of Computer Science and Technology, Xidian University, Xi'an 710126, China
[2] Yangtze Delta Region Institute (Quzhou), University of Electronic Science and Technology of China, Quzhou 324003, China
[3] Institute of Fundamental and Frontier Sciences, University of Electronic Science and Technology of China, Chengdu 610054, China
[4] School of Management, Xi'an Polytechnic University, Xi'an 710121, Shaanxi, China
*To whom correspondence should be addressed: Email: lyu@xidian.edu.cn and zouquan@nclab.net.
¶These authors should be considered as co-corresponding authors.

Keywords: Multiple Sequence Alignment, Maximum Exact Match, de Bruijn Graph, Parallel Algorithm Design

Abstract

Multiple sequence alignment (MSA) continues to be a central challenge in comparative genomics, where the quality of alignment plays a crucial role in determining the accuracy of downstream analyses. However, the challenge of large-scale alignment remains significant. This paper introduces deMEM, a novel and effective framework for DNA multiple sequence alignment, which enables existing MSA methods such as MAFFT, to handle extremely large sequences. deMEM is a three-stage alignment process: (i) representing Maximum Exact Matches using a de Bruijn graph and clustering them based on their area; (ii) employing a novel divide-and-conquer framework for alignment; (iii) profile-profile alignment between different clusters. deMEM enables existing

methods like MAFFT to align an extremely large number of sequences, including long sequences that cannot be directly aligned, such as those in a dataset of a thousand monkeypox virus genomes. The deMEM package is free and available at https://github.com/malabz/deMEM.

Introduction

Multiple sequence alignment (MSA) is a fundamental problem in bioinformatics. The quality of sequence alignment significantly impacts biological sequence analysis, especially that in next-generation sequencing [1, 2]. MSA results are widely used in various applications, including *de novo* genome assembly [3, 4], detection of single-cell genomes based on sequence alignment [5] and taxonomic assignment of newly sequenced data [6, 7].

In the last few decades, researchers have shown an increased interest on developing efficient MSA methods to enhance alignment accuracy. The guide tree for aligning MSA is a heuristic approach that aligns sequences based on a pre-built guide tree [8]. Guide tree can be categorized into two types: the center star guide tree and the distance estimation tree, with the latter serving as the basis for progressive alignment. The center star guide tree strategy tree has been utilized in HAlign series [9-12], while the progressive alignment method is employed in several tools, like Clustal [13], MAFFT [14], MUSCLE 3 [15] and FAMSA [16]. WMSA [17] combined center star tree and distance-based guide tree for alignment. The center star guide tree can align a large number of sequences with relatively low alignment quality. In contrast, the progressive alignment method generally produces slightly better-quality alignments, though it is still limited by the quality of the guide tree. To improve alignment quality, researchers have developed post-processing methods, such as ReformAlign [18], TPMA [19] and ReAlign-N [20].

To address the challenge of large-scale, high-quality MSA, researchers have developed seed-and-extension strategy and graph-based strategy [21-24]. The seed-and-extension strategy alignment to reduce the MSA problem by focusing on aligning and extending seed regions. Minimap2 [25] employed seed-and-extension strategy for pairwise sequence alignment. FAME [26] designed a state-of-art model for aligning long sequences through three steps: identifying common seeds based on the determined seed patterns, creating chains from seeds, and generating splitting alignments by chains. FMAlign series [27, 28], inspired by FAME, generate the multiple sequences chain by Maximum Exact Matches (MEM) based on FM-index. FMAlign2 further generates MEM based on LCP extension and supports sequence search with MEM. Graph-based alignment methods provide another approach to solve MSA. EulerAlign [29, 30] proposed MSA by generating and aligning sequences with consensus sequence determined by de Bruijn graph [31], and POA [32] proposed another graph representation to express and generate MSA. abPOA [33] significantly enhances computational efficiency through adaptive-band dynamic programming and SIMD parallelization. deBGA [34] utilizes aligning sequence reads based on de Bruijn graph. MEMs are fundamental for constructing de Bruijn graph in MSA. SplitMEM [35] is an efficient method for generating de Bruijn graph for multiple sequences or genomes. Baier et al. [36] enhanced SplitMEM by the Burrows-Wheeler Transform (BWT) to generate MEM, which significantly reduced the time complexity of the process to O($|\Sigma|$), which Σ is the length of all sequences.

Although there are huge numbers of MSA methods, most of these methods have suffered from various methodological limitations. Firstly, guide-tree based methods generally been restricted to the quality of guide tree and the principle "once a gap, always a gap", whereby any gap inserted during progressive profile-profile alignment remains fixed and cannot be corrected or refined in

subsequent steps. Secondly, due to the nature of MEM, seed-and-extension strategy-based methods are focusing the high similarity sequence alignment, with little attention of low similarity sequence alignment. Thirdly, POA is used for generating consensus sequences, particularly in third-generation sequencing, but is neither considered nor discussed by the sub-alignment methods in the seed-and-extension strategy, which limits the application of POA. Lastly, the research for de Bruijn graph has tended to focus on third-generation reads data analysis rather than MSA.

To address these challenges, we developed deMEM, an efficient and accurate multiple sequence alignment method based on divide-and-conquer strategy. It works by (a) splitting the sequences into clusters using enhanced version of SplitMEM, (b) aligning clusters into profiles by the MEMs, and (c) merge profiles by profile-profile alignment. Our method enhances alignment quality and demonstrates superior performance compared to traditional, seed-and-extension-free MSA strategies like MAFFT [14], WMSA [17] and abPOA [33] on low-similarity datasets. Additionally, it supports the alignment of extremely long sequences that these seed-and-extension-free methods cannot handle. Furthermore, deMEM outperforms sequence division methods like FMAlign2 [28] and FAME [26] in handling challenging alignment tasks, such as aligning extremely long sequences.

Methods

The framework of deMEM

Our developed framework is named deMEM. The architecture of deMEM can be described as follows (Figure 1):

- Step 1: Input the sequence file S, convert sequences to k-mer de Bruijn graph representation with determined threshold k, find MEMs and cluster sequences based on MEMs;
- Step 2: We generated n clusters in Step 1. For each cluster $C_i, i = 1,2,\ldots,n$, we align the cluster by our divide-and-conquer framework based on MEMs obtained from step 1. All clusters are aligned into profiles $P_i, i = 1,2,\ldots,n$;
- Step 3: Align n clusters by determined method, like MAFFT profile merge [14], WMSA [17] or abPOA [33].

Sequence Clustering by de Bruijn graph

We utilize a clustering algorithm based on the BWT-enhanced SplitMEM [35] algorithm [36]. For graph construction, we employ a disjoint-set union data structure [37] to represent cluster affiliation of each sequence. In particular, we modify Algorithm 2 in BWT-enhanced SplitMEM [36] to calculate the cluster. The pseudo code of modified algorithm is shown in Algorithm S1.

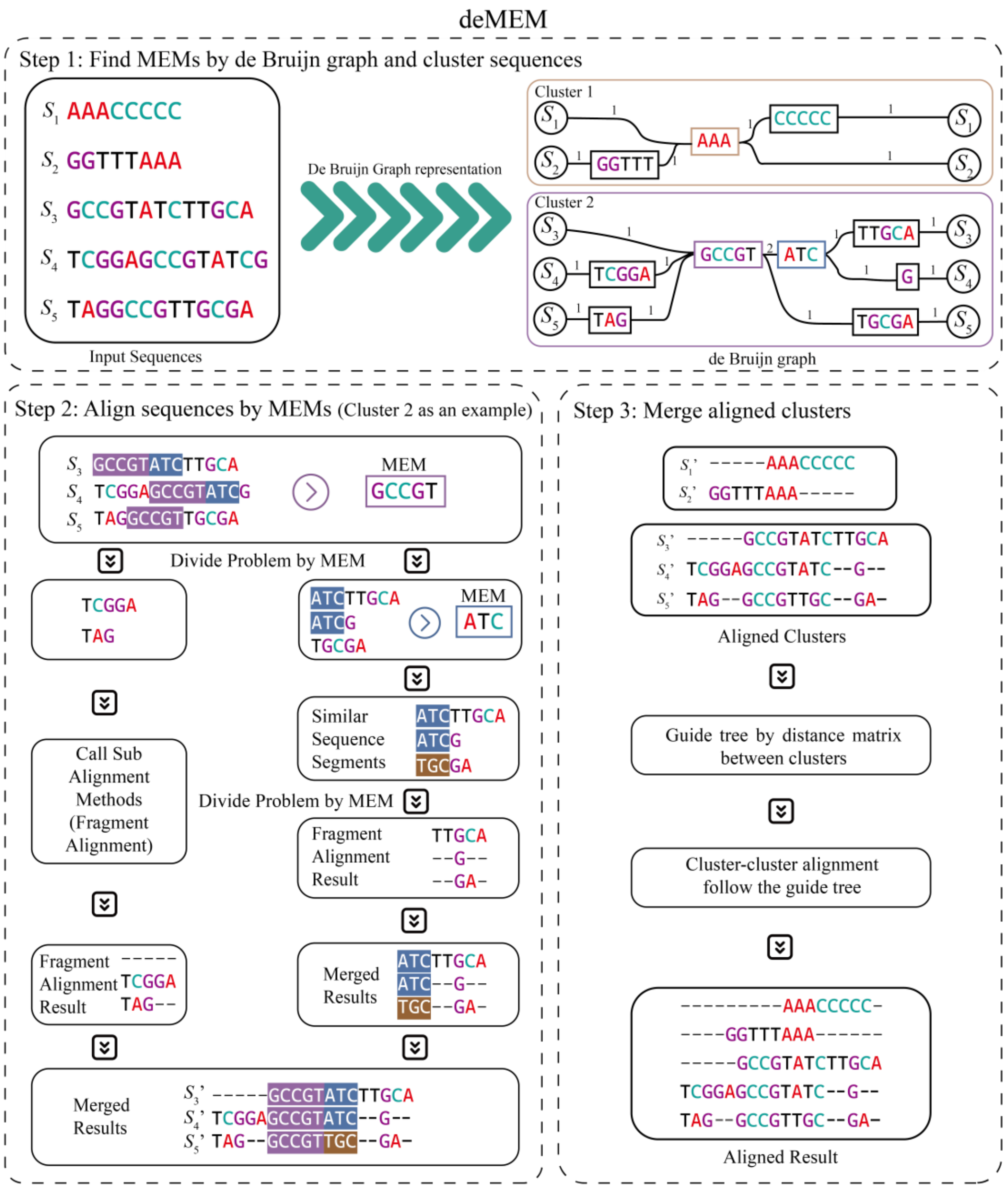

Figure 1: The framework of deMEM. (a) Find MEMs by de Bruijn graph and generate clusters: sequences are read and converted into clusters using SplitMEM [35], which represents the sequences as de Bruijn graph. The number on the edge in the de Bruijn graph represents the edge weight; (b) Align sequences by MEMs: For each cluster, sequences are aligned by a divide-and-conquer framework to process the MEMs. For every MEM, the alignment problem is divided into three subproblems: the left subproblem, the right subproblem, and the down subproblem. For

sequences not covered by any MEM, the SSW library [38] is used to identify similar fragments. If no similar fragment is found, the sequence is processed in the down subproblem. For subproblems without MEMs, external MSA methods such as MAFFT [14], WMSA [17] or abPOA [33] are employed for alignment. The resulting alignments are then merged to form an aligned cluster; (c) Merge aligned clusters to generate MSA: aligned clusters are combined using external profile-profile merge strategy, like MAFFT profile merge, WMSA or abPOA, to generate the final MSA result.

As shown in Algorithm S1, this algorithm uses disjoint-set data structure to measure the affiliation of sequences. When identical MEMs are found in different sequences, we merge the two disjoint-set unions that represent these sequences. Once the sequence affiliations are established, we apply the original strategy from SplitMEM, which uses Depth-First Search (DFS) to traverse the de Bruijn graph and generate MEMs for each cluster. After generating the MEMs for the clusters, we leverage this information to perform MSA for each cluster using the divide-and-conquer framework.

In conclusion, the time complexity of sequence clustering is $O(n(\log\sigma + \alpha(n,n)))$, where σ is the size of alphabet ($\sigma = 4$ in DNA sequences), and $\alpha(n,n)$ is inverse Ackermann function.

Divide-and-Conquer Framework for MSA Using MEMs

In this section, we describe the divide-and-conquer framework which the inputs are MEMs provided by Algorithm S1. The core of deMEM is the divide-and-conquer framework, where MEMs are used to split the alignment problem into smaller subproblems. For each cluster, we sort the MEMs by area, with the largest MEM is the first in array. Once sorting is complete, the sorted

MEMs with the sequences are assigned to cluster, fed the cluster into the divide-and-conquer framework to generate MSA. We start by defining MEM and its area in multiple strings, then define MEM with similar fragments and their area, to support the representation of sequences that lack MEMs but share similarity with them. After defining MEM with similar fragments, we proceed to introduce the alignment process within the divide-and-conquer framework, using the sorted MEMs to produce the final alignment results for the sequences corresponding to the clusters.

The definition of MEM

In this section, we will discuss the definition of MEM in detail. Considering the nature of MEMs, when different sequences share similar strings, our method treats these similar strings as fragments and merges them into the MEM. We first provide a formal definition of MEM, followed by the introduction of the concept of MEM with similar fragments, which allows us to represent these MEMs during the sequence alignment process.

The definition of MEM in two strings is exact matches between two strings that cannot be extended in either direction towards the beginning or end of two strings without allowing for a mismatch [39]. Since our problem involves multiple sequences, we need to extend the definition of MEM to accommodate multiple sequences. Definition 1 provides the definition of MEM in multiple strings:

Definition 1: MEM $\boldsymbol{M}$ in multiple strings $s_1, s_2, \dots, s_n$ is exact matches between multiple strings with match length L and that cannot be extended in either direction towards the beginning or end of multiple strings without allowing for a mismatch, which the intervals $[x_1, x_1 + L), [x_2, x_2 + L), \dots, [x_n, x_n + L)$ are corresponding with strings $s_1, s_2, \dots, s_n$. In other words, $\boldsymbol{M} =$

$\{L, (1, x_1), (2, x_2), \ldots, (n, x_n)\}$, which means the length of MEM is L, the MEM occurs at sequences $s_1, s_2, \ldots, s_n$ with begins at $x_1, x_2, \ldots, x_n$. The number of strings contains in $\boldsymbol{M}$ is $|\boldsymbol{M}| = n$. If MEM $\boldsymbol{M}$ only occurs at sequence $s_{ID_1}, s_{ID_2}, \ldots, s_{ID_d}$, which occurs at the intervals $[x_{ID_1}, x_{ID_1} + L), [x_{ID_2}, x_{ID_2} + L), \ldots, [x_{ID_d}, x_{ID_d} + L)$, we define the MEM $\boldsymbol{M} = \{L, (s_{ID_1}, x_{ID_1}), (s_{ID_2}, x_{ID_2}), \ldots, (s_{ID_d}, x_{ID_d})\}$, and the length of $\boldsymbol{M}$ is $|\boldsymbol{M}| = d$.

As shown in Definition 1, MEM in multiple strings can be common seeds in measuring the similarity of all sequences, but the distances in exact matches must be measured in sequences. As a result, we need to define the area of MEM for measuring the importance of every MEM. The definition of MEM area in multiple sequences is shown in Definition 2.

Definition 2: The area a of MEM $\boldsymbol{M}$ is defined as formula (1):

$$a = \max_{c}\left(\sum_{i}^{IDs}(L - |x_i - c|)\right) \tag{1}$$

where IDs is the set contains all sequence identifiers in this MEM, c means the “center” place of every sequence in MEM. It’s worth noting that, the definition of “center” refers to the position with the highest occurrence frequency in the MEM, which is set as the maximum area to ensure a unique area calculation. In particular, center c can be calculated as formula (2):

$$c = \underset{i \in IDs}{\text{max\_occur\_times}}(x_i) \tag{2}$$

where max_occur_times function calculates highest occurrence frequency in the list of start positions list $\{x_i, i \in IDs\}$. If multiple values have the same maximum frequency, the middle value (i.e., the median among the tied candidates) is selected. If there are two middle values, we compute the corresponding area a for each candidate x_i and choose the one with the maximum area. As shown in formula (2), the meaning of c is the "center" of MEM. If we choose any other $c' \neq c$, we cannot determine the area a uniquely. The example of area calculation is shown in Figure 2.

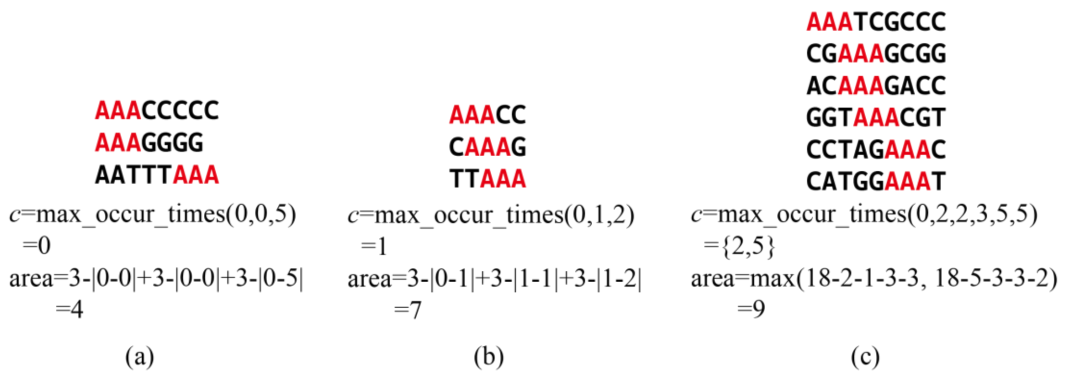

Figure 2: Examples for MEM and MEM area calculation. (a) The definition of MEM is $\boldsymbol{M}_1 = \{3, (0,0), (1,0), (2,5)\}$. The center of $\boldsymbol{M}_1$ is 0, because 0 occurs twice and 5 occurs only once. The area of MEM is calculated by formula (1); (b) The definition of MEM is $\boldsymbol{M}_2 = \{3, (0,0), (1,1), (2,2)\}$. The center of $\boldsymbol{M}_2$ is 1, because 0, 1 and 2 occurs once, we choose the medium number 1 to represent the center; (c) The definition of MEM is $\boldsymbol{M}_3 = \{3, (0,0), (1,2), (2,2), (3,3), (4,5), (5,5)\}$. The center of $\boldsymbol{M}_3$ can be 2 or 5, because both 2 and 5 occur twice in $\boldsymbol{M}_3$. Because 2 and 5 both are medium numbers, we need to calculate area to determine the center. If we choose 2 to be the center of $\boldsymbol{M}_3$, the area is 9; in other words, choose 5 to the center of $\boldsymbol{M}_3$, we can calculate the area of $\boldsymbol{M}_3$ is 5. As a result, we choose 2 to be center of $\boldsymbol{M}_3$.

In Definition 1, the MEM is defined as having the same length and across sequences. However, for the representation of similar fragments, we need to redefine MEM. We refer to this as the “MEM with similar fragments”. The definition of a MEM with similar fragments is provided in Definition 3, and the definition for the area of a MEM with similar fragments is given in Definition 4:

Definition 3: MEM with similar fragments $\boldsymbol{MX}$ in multiple strings $s_1, s_2, \dots, s_n$ is exact matches between multiple strings with match length L and that cannot be extended in either direction towards the beginning or end of multiple strings without allowing for a mismatch, after finding similar parts in MEM, strings $s_{n+1}, \dots, s_f$ found similar parts with $\boldsymbol{MX}$, which the interval

$[x_1, x_1 + L), [x_2, x_2 + L), \dots, [x_n, x_n + L), [x_{n+1}, y_{n+1}), \dots, [x_f, y_f)$ corresponding with string $s_1, s_2, \dots, s_f$. It's worth noting that, the similar parts are identified using the SSW library. In other words, $\boldsymbol{MX} = \{L, (ID_1, x_1, 0), \dots, (ID_n, x_n, 0), (ID_{n+1}, x_{n+1}, y_{n+1} - x_{n+1} - L), \dots, (ID_f, x_f, y_f - x_f - L)\}$, which means the length of MEM is L, the MEM occurs at sequences $ID_1, \dots, ID_n$ with begins at $x_1, \dots, x_n$, found similar strings in sequences $ID_{n+1}, \dots, ID_f$ with start at $x_{n+1}, \dots, x_f$ with length $y_{n+1} - x_{n+1}, \dots, y_f - x_f$. The number of strings contains in $\boldsymbol{MX}$ is $|\boldsymbol{MX}| = f$.

Definition 4: The area a of MEM with similar fragment $\boldsymbol{MX}$ is defined as formula (3):

$$a = \max_c \left(\sum_i^{IDs} (y_i - x_i - |x_i - c|) \right) \tag{3}$$

where IDs means sequence identifiers in this MEM, IDs is the set contains all sequence IDs in this MEM, c means the center place of every sequence in MEM. The calculation of center c is same as formula (2).

Align by sorted MEMs

In this section, we use sorted MEMs to make alignment. It's worth noting that, MEMs are sorted by area, with the MEM having the largest area placed first in the array. For every MEM, we follow its guidance to divide the corresponding sequences into three parts: left block, right block and down block (details shown in Figure 3). For sequences not included in the MEM, we use the SSW library [38] to find similar fragments and incorporate these fragments into the MEM, resulting in a new MEM referred to as "MEM with similar fragments". Sequences in the "MEM with similar fragments" are divided into left and right blocks, while sequences not included in the "MEM with similar fragments" are placed in the down block. The core of the divide-and-conquer framework for aligning sequences using sorted MEMs is shown in Algorithm S2, and the illustration of Algorithm

S2 is shown in Figure 3.

As shown in Algorithm S2, the alignment procedure can be summarized as follows: we sort MEM blocks according to their area, then call the main divide-and-conquer function for alignment based on MEM blocks. The internal logic of divide-and-conquer function can be concluded as follows: Firstly, the MEM may not contain all input sequences, therefore, we use the SSW algorithm to identify the sequences not covered by the MEM. After identifying these sequences, we merge the MEM with the corresponding intervals found by SSW. This combined block is treated as a MEM block with fragment parts. Using the MEM block with fragment parts, we divide the remaining sequences into three subproblems: align the left part, the right part and the down part. For each MEM, we determine the corresponding appearance in each sequence. The divide-and-conquer function is recursively called to align the subproblems. It is important to note that, if the down part is present, we must align the sequences in the down part with those in the MEM that contain the fragments, each of which has been aligned previously. To accelerate the divide-and-conquer procedure, we adopt a parallelization strategy similar to that used in FORAlign [40], using the fork-join model with work stealing to enhance computational efficiency.

Profile-profile Alignment and Fragment Alignment based on Existing Approaches

In previous sections, we introduced the alignment based on MEMs. In Figure 1 Step 2, after processing MEM, we call the sub alignment methods to make alignment. The sub alignment method is same as the father alignment method, except for the condition of no MEMs. If any part has no MEMs, we call the fragment alignment method. We determine the fragment alignment method like

MAFFT FFT-NS-1 [14], WMSA [17] or abPOA [33]. Like the sub alignment, we also compute profiles alignment by these methods. Since these methods cannot make profile-profile directly, we modified them to support profiles alignment. In particular, we made a list for representing sequences to profiles, and call profile-profile alignment to make the real alignment.

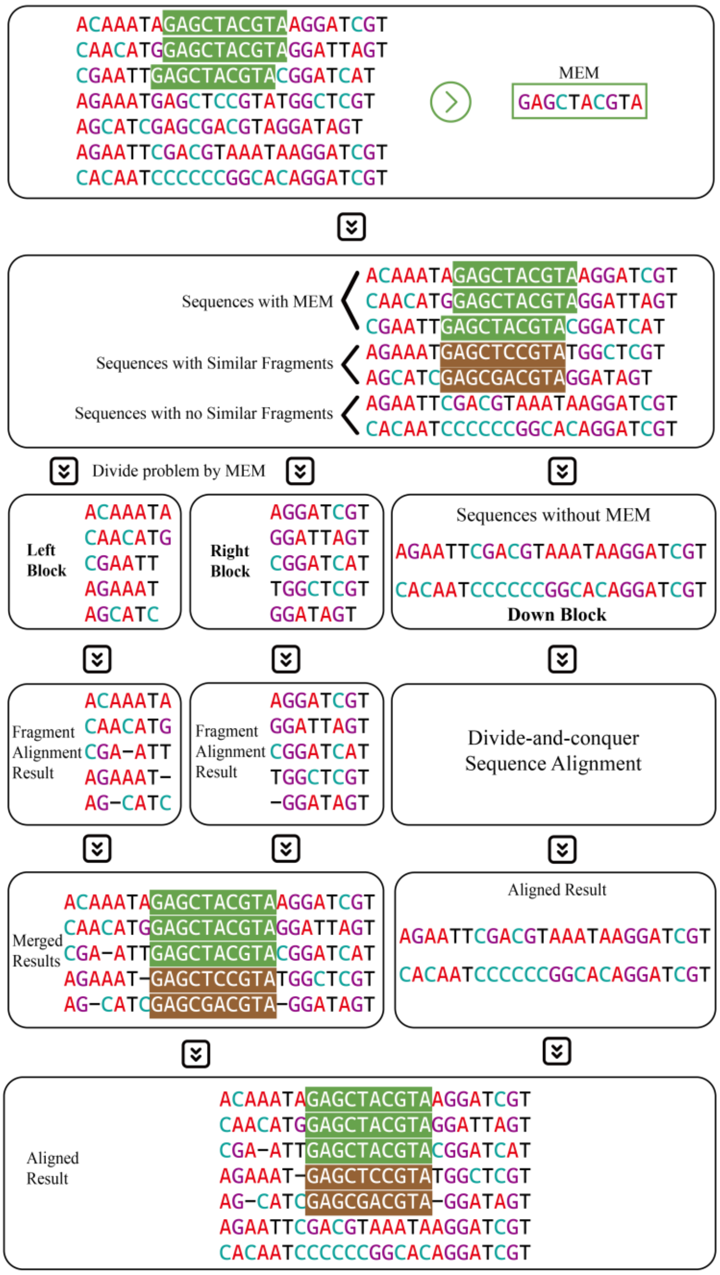

Figure 3: Details for aligning sequences by sorted MEMs. We found MEM “GAGCTACGTA” in these

sequences, the MEM occurs on the first, second and third sequences (colored area). After that, we call SSW function, found the similar parts "GAGCTCCGTA" and "GAGCGACTA". As a conclusion, the MEM with similar fragment is on the $1^{st}\sim5^{th}$ strings. After generating MEM with similar fragment, the left and right blocks is generated, and the down block is also generated. After finding similar sequence segment, we divide sequences into four parts: left block, right block, down block and MEM with similar fragment. The left, right and down block are aligned by sub alignment function (Sub_block_align function in Algorithm 2). We need to wait left and right block alignment to generate the sequence alignment result for the sequences in MEM with similar fragment. After generating sequence result, we need to wait down block to make alignment with sequences in MEM and sequences out MEM to generate the final result

Time and space analysis for the whole algorithm

deMEM can be divided on three modules: (A) Identifying MEMs and clustering sequences based on the de Bruijn graph; (B) Aligning sequences within each cluster using the divide-and-conquer framework based on MEMs to generate profiles; (C) Performing profile-profile alignment between different clusters. The time and space complexity analysis of the algorithm is presented below:

(A) Find MEMs and make clusters based on de Bruijn graph: assume we have n sequences, the length of sequences is S. We use BWT enhanced SplitMEM to find MEMs, the time complexity of this algorithm is $O\big((n+S)\log\sigma\big) \approx O(n+S)$, the space complexity is $O(n+S)$ to store all nodes in de Bruijn graph; make clusters based on MEMs, we need

to use disjoint-set for making clusters, the time complexity of clustering is $O((n+S)\alpha(n,n)) \approx O(n+S)$. In conclusion, the time and space complexity of step (A) is $O(n+S)$;

(B) Divide-and-conquer framework based on MEMs: assume we have n sequences with the minimum sequence length m, we can infer that the graph length in step (A) has maximum $O(nm)$ nodes. For every MEM, calculate the area needs $\mathrm{O}(n \log n)$ time. As for graph has at most $O(nm)$ nodes, the time complexity of sort is $O(nm\log(nm))$. Next, we align blocks by sorted MEMs. For every MEM, assume this size of MEM is $A \times y$, and the size of align region is $n \times m$, which A is the number of sequences, y is the length of MEM, n is the number of sequences, and m is the length of all sequences. Time complexity of region alignment is $T(n,m) = T(A,l) + T(A,r) + T(n-A,m) + S(n,m)$. We discuss the result of $S(n,m)$: firstly, we try to use SSW to find similarity part, we need to find $n-A$ sequences, the time complexity of SSW is $\mathrm{O}((n-A)ky)$ (use K-band) or $\mathrm{O}\big((n-A)my\big)$ (no K-band); then, we divide other MEMs into three parts, which need $O(nm)$ time; next, we wait the results of sub-function, merge and refine the results, which need $O(A)$; lastly, the border condition of the whole alignment is no MEMs in part, which we call MAFFT, WMSA or abPOA to make alignment, the time complexity of the whole algorithm may be influenced by determined algorithm: if we use WMSA with K-band, the time complexity of align is $O(xyk)$; otherwise, the time complexity of align is $O(xy^2)$; in conclusion, $S(n,m) = O(knm)$ (with K-band) ~ $O(nm^2)$ (without K-band). The analysis for $T(n,m)$ is similar with [41]. As for a result, $T(n,m) = O(knm)$ (with K-band) ~ $O(nm^2)$ (without K-band);

(C) Profile-profile alignment between different clusters: assume we generate C clusters in step (A), the maximum length of profile is P. the profile-profile alignment step requires a progressive profile-profile alignment process. Since $C \ll P$, so the time complexity of this step is $O(C^3 + C^2P^2) \approx O(C^2P^2)$, and the space complexity is $O(C^2P^2)$.

As a result, the time and space complexity of the whole deMEM algorithm is $O(Cnm^2 + C^2P^2)$, which C represents the number of clusters, n is the number of sequences, m is the length of the longest sequence, P is the length of the aligned profiles.

Datasets and measurement

To evaluate the alignment results of our proposed method, we developed a software package called deMEM. In this section, we first introduce the datasets used to compare deMEM with other methods, followed by a description of the test methods. Finally, we outline the evaluation metrics and computational resources utilized in this experiment.

Experimental Datasets: Because deMEM divides sequences into multiple parts, multiple conditions are required to demonstrate the advantages of our method. To comprehensively evaluate its performance, we conducted experiments on both real and simulated datasets. Thus, we choose the following datasets, shown in Table 1 and Table 2:

Table 1 Description of the datasets tested in deMEM (Real data)

Dataset name	Source of dataset	Sequences	Average sequence length	Length distribution	References
mt1x	Mt genomes	672	16568.3	16555~16578	[11, 17, 26,

mt20x		13440			42]
Complete156	SARS-CoV-2	156	29855.1	29409~29927	[17, 42]
Mix1t		1024	27556.8	64~29981	
MPoX	Monkey Pox virus	1739	197084.9	183230~210918	[42, 43]
Variola	Variola virus	4	186374.3	186064~186677	[26]
Mycoplasma	*Mycoplasma bovis*		579708.8	579504~579977	
Streptococcus	*Streptococcus pneumonia*		2160522	2111882~ 2184682	
Ecoli	*Escherichia coli*		4633445.8	4578159~ 4686137	
Nerisseria	*Nerisseria meningitidis*	5	2190087.6	2145295~ 2272360	First collected
23sr	*Mycobacterium* 23S rRNA	641	3113.1	1909~3485	[11]

Table 2 Description of the datasets tested in deMEM (Simulated data)

Test Name	Sequences	Average length	Length distribution	Test cases	Reference
RNA-255	255	1527	1518~1542	10	[44]
RNA-511	511	1528	1518~1542		
RNA-1023	1023	1527	1517~1542		

RNA-2047	2047	1527	1517~1542		
RNA-4095	4095	1527	1516~1542		
mt-similarity	112	15860±115	15719±220~ 15992±12	9	[11]
SARS-CoV-2-similarity	112	29675±118	29404±316 ~ 30000±0		

In Table 1, we newly collected the *Nerisseria meningitidis* sequences to show the quality for our methods.

Experimental methods: We compared our method with FAME [26] and FMAlign2 [28], both of which use chain-based strategies. As described before, we employed MAFFT FFT-NS-1 [14], abPOA [33] and WMSA [17] to calculate sub alignments. Our experiments can be divided into two main parts: (a) evaluating the improvements by FAME, FMAlign2 and our method for sub alignment strategies like MAFFT FFT-NS-1; (b) compare the different sub alignment strategies like MAFFT FFT-NS-1, abPOA and WMSA, under two approaches: treating all sequences as a cluster (methods *-H in result tables) or grouping sequences into multiple clusters (methods *-L in result tables). It is worth noting that if deMEM does not find any MEMs, the program falls back to the original alignment method. For consistency, since different alignment methods employ distinct scoring systems (e.g., abPOA uses two-piece gap affine penalty scoring system, while WMSA and MAFFT use simple gap affine penalty scoring system), all alignment methods were evaluated using their default parameters.

Experimental Metrics: We measure the real data alignment results by SP score introduced in [27], with match score=0, mismatch score=-1 and gap score=-2. A lower SP score indicates fewer

inserted gaps, reflecting improved alignment consistency and overall quality. For simulated datasets, we use Q and TC score designed in MUSCLE [15] for measuring the results for all methods.

Computational Resources: Our experiment is tested on a workstation with 1TB main memory, an Intel(R) Xeon(R) Gold 6230 CPU processor with 80 cores with 2.10GHz CPU frequency under the Ubuntu 20.04 operating system.

Results

Experimental Results on real datasets

In this section, we present the results of real datasets. A summary of results is provided in Figure 4, which the detailed results are shown in Table S1. From Table S1 and Figure 4, we observe that our method improves the SP Scores in real datasets, particularly in extremely large datasets. Compared to seed-and-extension MSA methods, our method achieved higher SP scores more than FMAlign2 and FAME. A key advantage of deMEM is its ability to integrate both vertical and horizontal sequence information, leading to more comprehensive sequence alignments. For extremely large and long sequences, such as those in the MPoX dataset, although FAME achieved faster alignment with lower memory consumption, it produced lower-quality results compared to deMEM. Specifically, deMEM improved alignment quality by approximately 50.3% over FAME and 32.0% over FMAlign2 on the MPoX dataset.

Comparison between seed-and-extension MSA methods

To evaluate the enhancement for SP scores between FMAlign2, FAME and our method, we independently ran the MAFFT FFT-NS-1 method and calculated the SP score using the

aforementioned methods. The results are presented in Table 3. Due to the limitations of MAFFT FFT-NS-1, it can only directly align sequences in the cases listed in Table 3. It's worth noting that, compared to FAME and FMAlign2, our method enhanced the performance of FFT-NS-1 when aligning large sequences. Specifically, compared to FAME, deMEM can align extremely long sequences, without any decrease in SP scores and demonstrates improved robustness, consistently aligning sequences with stability. Compared to FMAlign2, our method performs better on mt sequences. For extremely large and long sequences, such as *Mycoplasma bovis* sequences, deMEM offers modest improvements in alignment quality but uses significantly less memory.

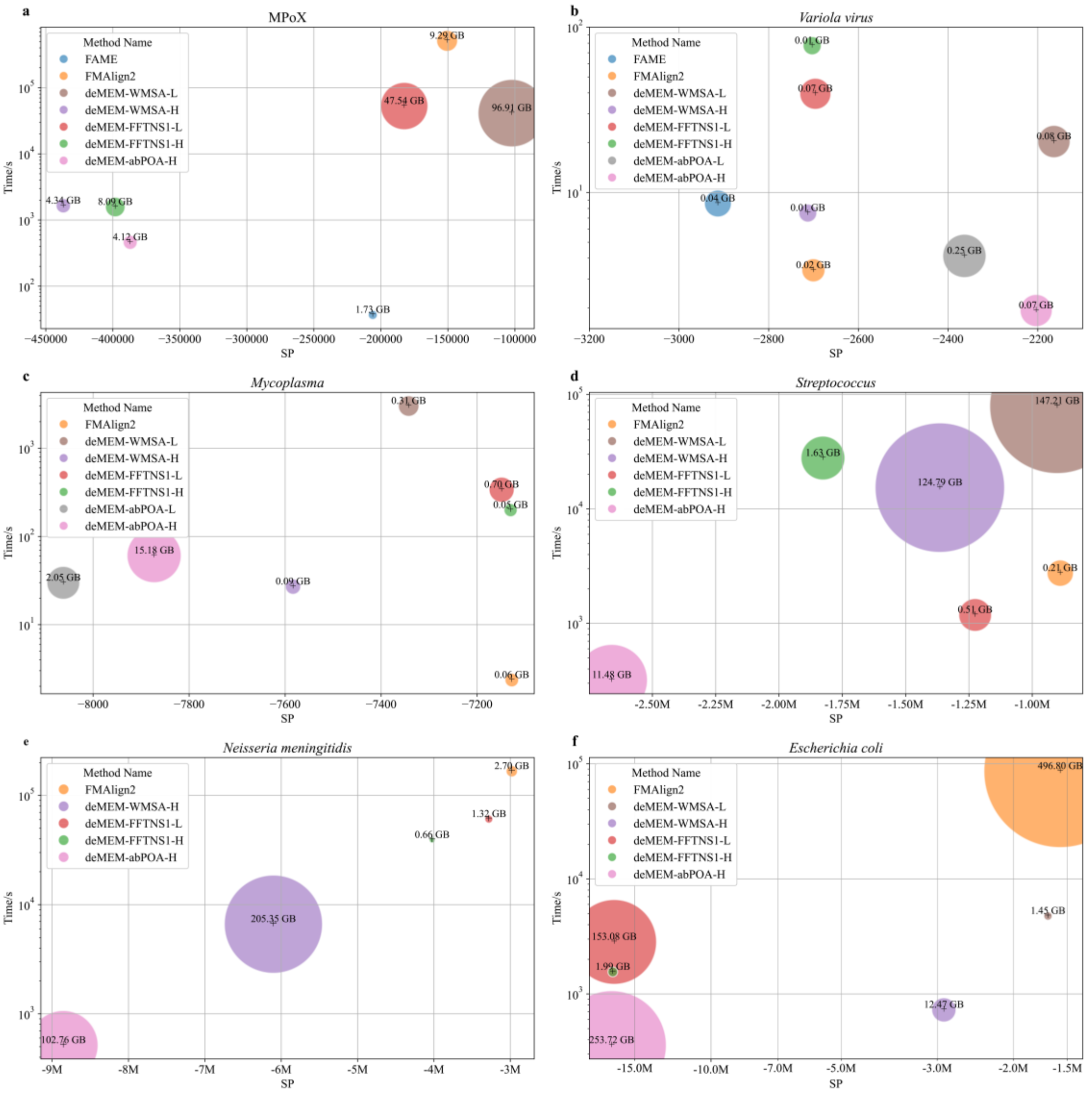

Figure 4. Illustration of real dataset results in different methods. The area of circle is the memory

usage of the determined method. This figure shows the best SP values between same method, excluded the out of memory methods. Lower absolute SP scores indicate better alignment quality

Table 3 Result difference between seed-and-extension methods and MAFFT FFT-NS-1. “Block Size” in this table means the maximum SP score aligned by the determined MEM block size. ΔSP means the difference value between the determined method with MAFFT FFT-NS-1

Method Name	mt1x		mt20x		*Mycoplasma bovis*	
	Block Size	ΔSP	Block Size	ΔSP	Block Size	ΔSP
FAME	-	-52.3	-	-52.2	-	-
FMAlign2	20	9.1	500	7.4	500	**21.2**
deMEM-FFTNS1-L	50	6.2	15000	7.6	10000	0.0
deMEM-FFTNS1-H	100	**11.6**	100	**11.6**	5000	18.7

Comparison between sub alignment methods in deMEM

deMEM supports various sub alignment methods, including abPOA, FFT-NS-1 and WMSA. The quality and speed of alignment are influenced by the choice of sub-alignment method. Each method has its own advantages: abPOA can align smaller sequences quickly with minimal memory usage, but it is less suitable for long and large sequences; FFT-NS-1 is optimal for aligning highly similar sequences; WMSA fits in aligning huge and long sequences with low similarity. As shown in Figure 4, our method effectively aligns extremely large datasets, demonstrating its scalability.

To further evaluate the impact between different methods, we tested the mt1x and mt20x

datasets use these sub-alignment methods, as shown in Figure 5. The results indicate that sub-alignment methods primarily influence memory and runtime efficiency. Notably, abPOA exhibited slow alignment speeds because it was executed on a single-threaded process, significantly affecting performance.

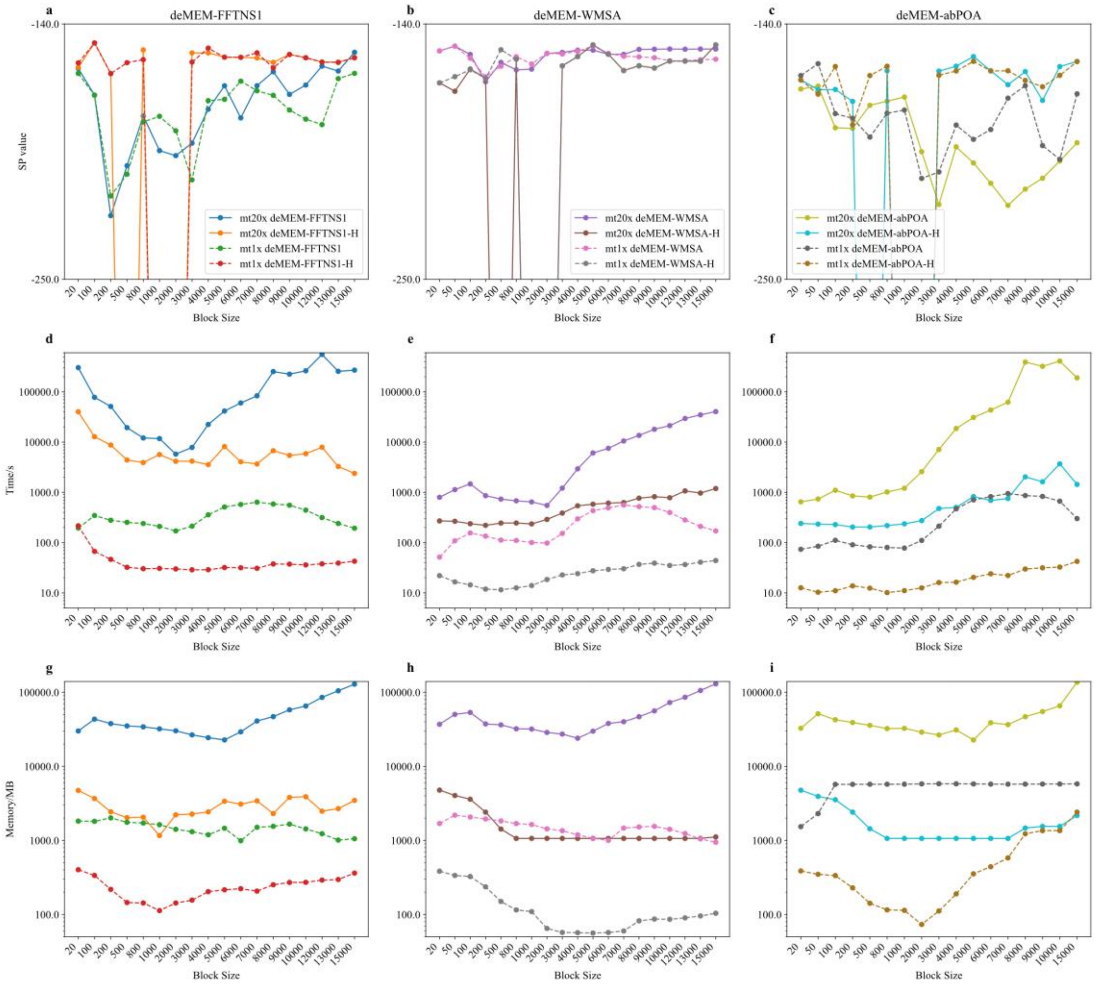

Figure 5 SP Scores, time and memory change with block size in mt1x and mt20x datasets.

Experimental Results on simulated datasets

Simulated datasets provide real alignment results, allowing for a direct comparison between alignment results and actual alignments. We evaluated our method on two simulation datasets, as

shown in Figure 6 and Figure 7. Figure 6 presents the Q and TC score improvements between sub alignment methods and raw alignment methods. Our method achieved slightly higher quality alignment result compared to WMSA raw methods in simulated RNA test cases. Figure 7 highlights the impact of small MEM blocks on alignment accuracy. The results indicate that incorporating small MEM blocks enhances both Q score and TC score, leading to improved overall alignment quality.

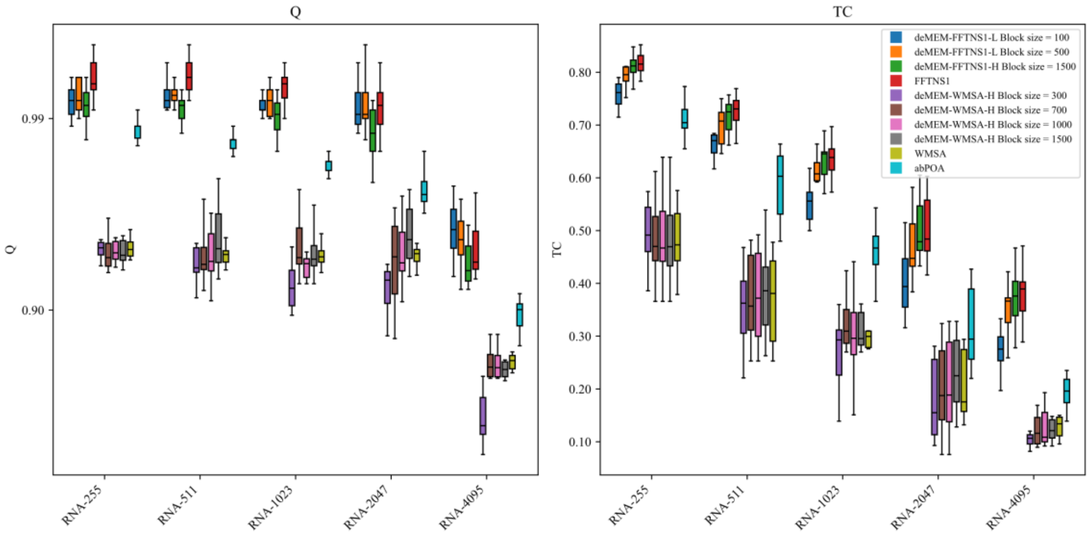

Figure 6 Q and TC Score in RNA simulated tests. Only show the enhanced deMEM methods.

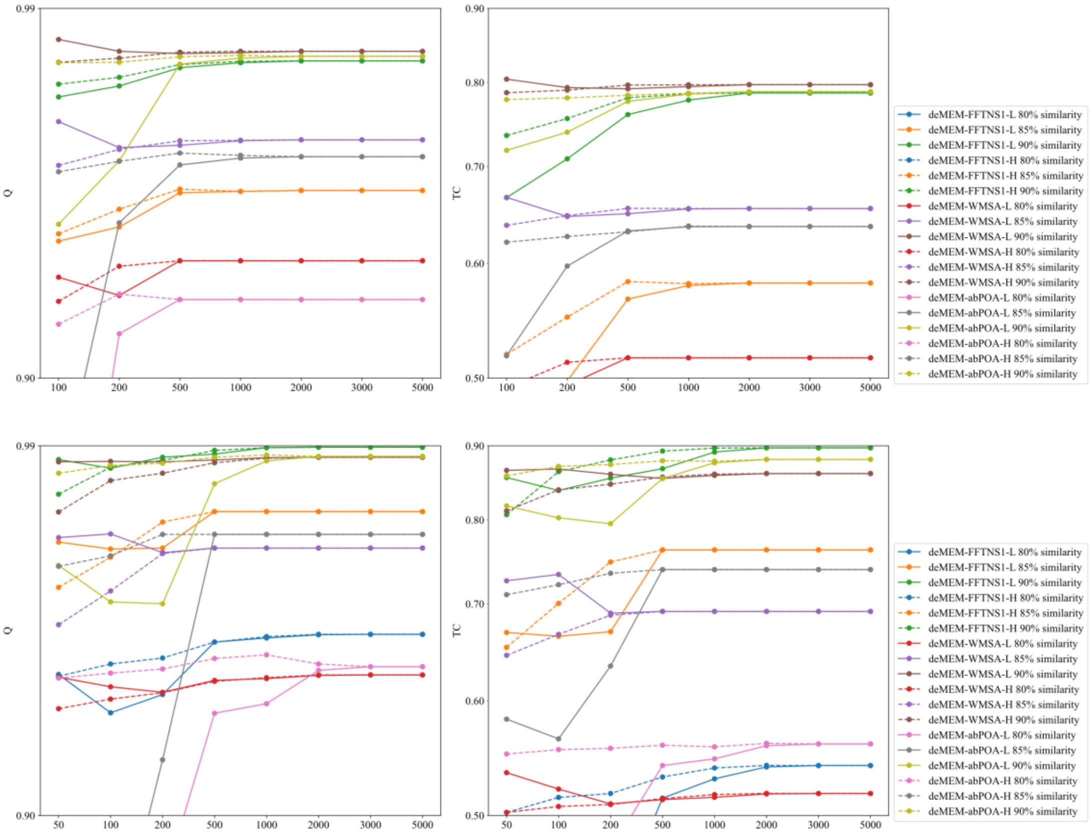

Figure 7 Q and TC score in two similarity datasets. Only shown Q and TC score in 80%~90% similarity. Upper: mt-similarity dataset; lower: SARS-CoV-2 similarity dataset

Conclusion

In this paper, we introduced deMEM, a novel framework for MSA, supports horizontal and vertical problem decomposition based on MEMs. The method follows a three-step approach: First, deMEM identifies MEMs and clusters with all input sequences using de Bruijn graph. Next, sequences in clusters are aligned into profiles by divide-and-conquer framework based on MEMs. Lastly, profiles are progressively aligned to obtain the final MSA. The core of deMEM is the divide-and-conquer framework, where MEMs are used to split the alignment problem into smaller subproblems in both vertical and horizonal conditions. Experiments showed that deMEM

outperforms its counterpart tools at large scale data, such as a thousand monkeypox genomes, which improved alignment quality by approximately 50.3% over FAME and 32.0% over FMAlign2 on the MPoX dataset. Additionally, experiments on simulated data indicate that deMEM achieves improved alignment accuracy with slight enhancements. As a future work, we plan to improve deMEM method to optimize the space usage to support extremely long and large-scale sequences alignment. Moreover, we will focus on protein sequence alignment in the future to extend the application of our method.

The deMEM package is freely available at https://github.com/malabz/deMEM. It has been tested on the Linux and Windows. This package is also available on conda.

Declaration of competing interest

The authors declare that they have no known competing financial interests or personal relationships that could have appeared to influence the work reported in this paper.

Data availability

The datasets and are publicly available at https://doi.org/10.5281/zenodo.14989520.

Availability of source code and requirements

- Project name: deMEM
- Project home page: https://github.com/malabz/deMEM
- Operating system(s): Linux (Recommended) & Windows
- Programming language: C++
- Other requirements: Anaconda (Recommended)
- License: MIT
- Any restrictions to use by non-academics: License needed

Funding

This work was supported by the National Natural Science Foundation of China [grant number 62472344, 62452107, 62072353 and 62272065]; and Xidian University Specially Funded Project for Interdisciplinary Exploration (No. TZJH2024027).

Acknowledgments

We acknowledge the help from the other group members: Yixiao Zhai, Tong Zhou, and Qinzhong Tian for providing critical opinions during the preparation.

References

1. Wang GH, Liu YL, Zhu DX, Klau GW and Feng WX. Bioinformatics Methods and Biological Interpretation for Next-Generation Sequencing Data. Biomed Res Int. 2015;2015 doi:10.1155/2015/690873.
2. Yin C, Wang R, Qiao J, Shi H, Duan H, Jiang X, et al. NanoCon: contrastive learning-based deep hybrid network for nanopore methylation detection. Bioinformatics. 2024;40 2:btae046.
3. Chitsaz H, Yee-Greenbaum JL, Tesler G, Lombardo MJ, Dupont CL, Badger JH, et al. Efficient de novo assembly of single-cell bacterial genomes from short-read data sets. Nat Biotechnol. 2011;29 10:915-21. doi:10.1038/nbt.1966.
4. Sohn JI and Nam JW. The present and future of de novo whole-genome assembly. Brief Bioinform. 2018;19 1:23-40. doi:10.1093/bib/bbw096.
5. Muyas F, Sauer CM, Valle-Inclan JE, Li R, Rahbari R, Mitchell TJ, et al. De novo detection of somatic mutations in high-throughput single-cell profiling data sets. Nat Biotechnol. 2024;42 5:758-67. doi:10.1038/s41587-023-01863-z.
6. Tian Q, Zhang P, Zhai Y, Wang Y, Zou Q and Stairs C. Application and Comparison of Machine Learning and Database-Based Methods in Taxonomic Classification of High-Throughput Sequencing Data. Genome Biology and Evolution. 2024;16 5 doi:10.1093/gbe/evae102.
7. Wang L, Ding Y, Tiwari P, Xu J, Lu W, Muhammad K, et al. A deep multiple kernel learning-based higher-order fuzzy inference system for identifying DNA N4-methylcytosine sites. Information Sciences. 2023;630:40-52. doi:10.1016/j.ins.2023.01.149.
8. Chao J, Tang F and Xu L. Developments in Algorithms for Sequence Alignment: A Review. Biomolecules. 2022;12 4 doi:10.3390/biom12040546.

9. Zou Q, Hu Q, Guo M and Wang G. HAlign: Fast multiple similar DNA/RNA sequence alignment based on the centre star strategy. Bioinformatics. 2015;31 15:2475-81. doi:10.1093/bioinformatics/btv177.
10. Wan S and Zou Q. HAlign-II: efficient ultra-large multiple sequence alignment and phylogenetic tree reconstruction with distributed and parallel computing. Algorithms Mol Biol. 2017;12:25. doi:10.1186/s13015-017-0116-x.
11. Tang F, Chao J, Wei Y, Yang F, Zhai Y, Xu L, et al. HAlign 3: Fast Multiple Alignment of Ultra-Large Numbers of Similar DNA/RNA Sequences. Mol Biol Evol. 2022;39 8 doi:10.1093/molbev/msac166.
12. Zhou T, Zhang P, Zou Q and Han W. HAlign 4: a new strategy for rapidly aligning millions of sequences. Bioinformatics. 2024;40 12 doi:10.1093/bioinformatics/btae718.
13. Higgins DG and Sharp PM. CLUSTAL: a package for performing multiple sequence alignment on a microcomputer. Gene. 1988;73 1:237-44. doi:10.1016/0378-1119(88)90330-7.
14. Katoh K, Misawa K, Kuma K and Miyata T. MAFFT: a novel method for rapid multiple sequence alignment based on fast Fourier transform. Nucleic Acids Res. 2002;30 14:3059-66. doi:DOI 10.1093/nar/gkf436.
15. Edgar RC. MUSCLE: multiple sequence alignment with high accuracy and high throughput. Nucleic Acids Res. 2004;32 5:1792-7. doi:10.1093/nar/gkh340.
16. Deorowicz S, Debudaj-Grabysz A and Gudys A. FAMSA: Fast and accurate multiple sequence alignment of huge protein families. Sci Rep. 2016;6:33964. doi:10.1038/srep33964.
17. Wei Y, Zou Q, Tang F and Yu L. WMSA: a novel method for multiple sequence alignment of DNA sequences. Bioinformatics. 2022;38 22:5019-25. doi:10.1093/bioinformatics/btac658.
18. Lyras DP and Metzler D. ReformAlign: improved multiple sequence alignments using a profile-based meta-alignment approach. BMC Bioinformatics. 2014;15 1:265. doi:10.1186/1471-2105-15-265.
19. Zhai Y, Chao J, Wang Y, Zhang P, Tang F and Zou Q. TPMA: A two pointers meta-alignment tool to ensemble different multiple nucleic acid sequence alignments. PLOS Computational Biology. 2024;20 4 doi:10.1371/journal.pcbi.1011988.
20. Zhai Y, Zhou T, Wei Y, Zou Q and Wang Y. ReAlign-N: an integrated realignment approach for multiple nucleic acid sequence alignment, combining global and local realignments. NAR Genomics and Bioinformatics. 2024;6 4 doi:10.1093/nargab/lqae170.
21. Liu Y, Shen X, Gong Y, Liu Y, Song B and Zeng X. Sequence Alignment/Map format: a comprehensive review of approaches and applications. Briefings in Bioinformatics. 2024;24 5:bbad320.
22. Qiao J, Jin J, Yu H and Wei L. Towards Retraining-free RNA Modification Prediction with Incremental Learning. Information Sciences. 2024:120105.
23. Li H and Liu B. BioSeq-Diabolo: Biological sequence similarity analysis using Diabolo. PLOS Computational Biology. 2023;19 6:e1011214.
24. Li H, Pang Y and Liu B. BioSeq-BLM: a platform for analyzing DNA, RNA, and protein sequences based on biological language models. Nucleic Acids Res. 2021;49

22:e129.
25. Li H. Minimap2: pairwise alignment for nucleotide sequences. Bioinformatics. 2018;34 18:3094-100. doi:10.1093/bioinformatics/bty191.
26. Naznooshsadat E, Elham P and Ali S-Z. FAME: fast and memory efficient multiple sequences alignment tool through compatible chain of roots. Bioinformatics. 2020;36 12:3662-8.
27. Liu H, Zou Q and Xu Y. A novel fast multiple nucleotide sequence alignment method based on FM-index. Brief Bioinform. 2022;23 1 doi:10.1093/bib/bbab519.
28. Zhang P, Liu H, Wei Y, Zhai Y, Tian Q and Zou Q. FMAlign2: a novel fast multiple nucleotide sequence alignment method for ultralong datasets. Bioinformatics. 2024;40 1 doi:10.1093/bioinformatics/btae014.
29. Zhang Y and Waterman MS. An Eulerian path approach to global multiple alignment for DNA sequences. J Comput Biol. 2003;10 6:803-19. doi:10.1089/106652703322756096.
30. Zhang Y and Waterman MS. An Eulerian path approach to local multiple alignment for DNA sequences. Proc Natl Acad Sci U S A. 2005;102 5:1285-90. doi:10.1073/pnas.0409240102.
31. De Bruijn NG. A combinatorial problem. Proceedings of the Section of Sciences of the Koninklijke Nederlandse Akademie van Wetenschappen te Amsterdam. 1946;49 7:758-64.
32. Lee C, Grasso C and Sharlow MF. Multiple sequence alignment using partial order graphs. Bioinformatics. 2002;18 3:452-64. doi:10.1093/bioinformatics/18.3.452.
33. Gao Y, Liu Y, Ma Y, Liu B, Wang Y and Xing Y. abPOA: an SIMD-based C library for fast partial order alignment using adaptive band. Bioinformatics. 2021;37 15:2209-11. doi:10.1093/bioinformatics/btaa963.
34. Liu B, Guo H, Brudno M and Wang Y. deBGA: read alignment with de Bruijn graph-based seed and extension. Bioinformatics. 2016;32 21:3224-32. doi:10.1093/bioinformatics/btw371.
35. Marcus S, Lee H and Schatz MC. SplitMEM: a graphical algorithm for pan-genome analysis with suffix skips. Bioinformatics. 2014;30 24:3476-83. doi:10.1093/bioinformatics/btu756.
36. Baier U, Beller T and Ohlebusch E. Graphical pan-genome analysis with compressed suffix trees and the Burrows-Wheeler transform. Bioinformatics. 2016;32 4:497-504. doi:10.1093/bioinformatics/btv603.
37. Tarjan RE. A class of algorithms which require nonlinear time to maintain disjoint sets. Journal of Computer and System Sciences. 1979;18 2:110-27. doi:10.1016/0022-0000(79)90042-4.
38. Zhao M, Lee WP, Garrison EP and Marth GT. SSW library: an SIMD Smith-Waterman C/C++ library for use in genomic applications. PLoS One. 2013;8 12:e82138. doi:10.1371/journal.pone.0082138.
39. Khan Z, Bloom JS, Kruglyak L and Singh M. A practical algorithm for finding maximal exact matches in large sequence datasets using sparse suffix arrays. Bioinformatics. 2009;25 13:1609-16. doi:10.1093/bioinformatics/btp275.
40. Wei Y, Zhou T, Zhai Y, Yu L and Zou Q. FORAlign: accelerating gap-affine DNA

pairwise sequence alignment using FOR-blocks based on Four Russians approach with linear space complexity. Brief Bioinform. 2025;26 1 doi:10.1093/bib/bbaf061.
41. Hirschberg DS. A linear space algorithm for computing maximal common subsequences. Communications of the ACM. 1975;18 6:341-3. doi:10.1145/360825.360861.
42. Kong X, Shen C and Tang J. CUK-Band: A CUDA-Based Multiple Genomic Sequence Alignment on GPU. In: Singapore, 2024, pp.84-95. Springer Nature Singapore.
43. Ma Y, Chen M, Bao Y and Song S. MPoxVR: A comprehensive genomic resource for monkeypox virus variant surveillance. The Innovation. 2022;3 5 doi:10.1016/j.xinn.2022.100296.
44. Chen J, Chao J, Liu H, Yang F, Zou Q and Tang F. WMSA 2: a multiple DNA/RNA sequence alignment tool implemented with accurate progressive mode and a fast win-win mode combining the center star and progressive strategies. Brief Bioinform. 2023;24 4 doi:10.1093/bib/bbad190.

Click here to access/download

Supplementary Material

Algorithms_supplementary_material.docx

Supplementary Material (Table S1)

Click here to access/download
Supplementary Material
Table S1_supplementary_material.docx

Reviewers' Comments to Author:

Reviewer #1: This manuscript introduces deMEM, a novel framework for multiple sequence alignment (MSA) of large-scale DNA datasets. The work addresses the significant challenge of applying existing MSA tools to datasets with an extremely large number of long sequences. The core contribution is a three-stage, divide-and-conquer strategy that first uses a de Bruijn graph to identify Maximum Exact Matches (MEMs) for clustering sequences. Subsequently, it aligns sequences within each cluster using a recursive, MEM-based approach before finally merging the resulting profiles. In essence, deMEM functions as a scalable wrapper that enables established MSA methods to process datasets that would otherwise be computationally intractable. The results show that it improves alignment quality on large real-world datasets, such as a collection of a thousand monkeypox genomes, outperforming other methods designed for large-scale alignment.

Response: Thank you very much for taking the time to comment on our manuscript. Your questions have been addressed point by point below.

Given the authors' extensive previous work on large-scale MSA tools like the HAlign series, the manuscript would be significantly strengthened by a discussion comparing deMEM to these methods. It is currently unclear where deMEM fits within this body of work—is it an alternative, a successor, or a tool for a different niche? A direct comparison or at least a clear positioning statement is needed.

Response: Thank you for your comments. deMEM is a novel divide-and-conquer based MSA **tool**, which supports horizonal and vertical division for aligning extremely large sequences like a thousand monkeypox sequences.

While the manuscript is generally well-written, the core contributions could be emphasized more clearly. The authors might consider adding a bulleted list of contributions in the introduction to help the reader quickly grasp the key innovations of deMEM.

Response: We added lists in the introduction:

deMEM is a three-stage alignment process: (i) representing Maximum Exact Matches using a de Bruijn graph and clustering them based on their area; (ii) employing a novel divide-and-conquer framework for alignment; (iii) profile-profile alignment between different clusters.

The primary weakness of the manuscript is the presentation of the results, which in their current form are extremely hard to read and often confusing. Table 3, for instance, is a dense and challenging nightmare to parse. To improve clarity, I suggest showing results for fewer methods or parameter configurations in the main text, perhaps choosing a single representative block size and moving the rest to supplementary materials. It would also be beneficial to use multiple, smaller tables for the different datasets. The readability could be enhanced by changing the units to be more intuitive, such as using GB for memory and minutes for time, to minimize the number of printed digits. For the SP score, printing a delta from an optimal value could make comparisons easier. Adding colors to distinguish the proposed methods from the state of the art would also

help.
Response: Thanks for pointing this. The content of Table 3 is identical to that of Figure 4, so we have moved Table 3 to the Supplementary Materials (Table S1) to improve clarity for readers. Due to the differences in problem size (e.g., 23sr and mt1x used less memory), we did not modify the memory usage shown in megabytes (MB).

Figure 4 is also hard to read, primarily because the current state of its axes is confusing. Both axes should represent metrics where either lower is better or higher is better. It would be useful to highlight the methods on the Pareto front. Once again, choosing a color palette with a clear semantic meaning would help the reader follow what is state of the art versus what is proposed, with related tools having similar colors. It is also unclear why sub-figure (b) is not in log time when the others are; consider putting both axes in log scale. For Figures 6 and 7, please consider using a meaningful color palette with similar colors for a given tool.

Response: In SP axis, the lower absolute SP scores indicate better alignment quality. We have added "Lower absolute SP scores indicate better alignment quality" in the legend of Figure 4. We have changed the sub-figure (b) to log time. The Figures 6 and 7 showed the different simulated test cases, and Figure 6 showed the results in the fixed block size, and Figure 7 showed the results in the fixed dataset. Therefore, the color schemes between the two figures may differ.

The conclusion of the paper is incredibly vague. The statement that deMEM "outperforms its counterpart tools at large scale data" is not sufficiently supported without specifying the context. The authors should explicitly state on which metrics, such as SP score or runtime, it outperforms, and over which specific tools. Similarly, the phrase "achieves improved alignment accuracy with slight enhancement" is ambiguous. It must be clarified what the baseline for this improvement is and whether it is an enhancement or an overall improved alignment.

Response: Thanks for your comments. We have expanded the conclusion section to include a discussion of the experimental results:
Experiments showed that deMEM outperforms its counterpart tools at large scale data, such as a thousand monkeypox genomes, which improved alignment quality by approximately 50.3% over FAME and 32.0% over FMAlign2 on the MPoX dataset.

Reviewer #2: The paper describes a new method for multiple sequence alignment. The core idea is to use the most reliable alignment blocks first and use them to split the problem into subproblems. While the general approach is interesting the poor quality of writing does not allow to clearly understand the details. The results presented in the paper are not convincing. Please find below a more detailed list of comments.

Response: Thank you very much for taking the time to comment on our manuscript. Your questions have been addressed point by point below.

List of unclear statements in the paper:
Page 3: "once a gap always a gap" principle is not explained properly.

Response: Thanks for pointing this. We have added the explanation of the principle “once a gap always a gap”: “whereby any gap inserted during progressive profile-profile alignment remains fixed and cannot be corrected or refined in subsequent steps”.

Page 4: "POA is limited for finding consensus sequences." this is included in a list of general criticisms of existing approaches. Why specifically POA deficiency is included in this list? Is it different from other methods?

Response: The primary application of POA methods is to generate consensus sequences in third-generation sequencing. In this revision, we discuss the potential for integrating sub-alignment methods within the seed-and-extension strategy, and we have modified the corresponding explanation:
Thirdly, POA is used for generating consensus sequences, particularly in third-generation sequencing, but is neither considered nor discussed by the sub-alignment methods in the seed-and-extension strategy, which limits the application of POA.

Page 4: "Furthermore, deMEM made better performance on diving sequences methods" I am not sure what is meant by diving sequences and it looks like the language is at least a part of the problem.

Response: Thanks for pointing this. This is a typo, we have modified “diving” to “dividing”.

Page 5: What is len_sum, number, G, and cur in the pseudocode? Why does pseudo-code contain details of disjoint set implementation, but uses multiple variables, which are never introduced? Where is the output of the procedure? No return line is present in the code and no mention of C (which is declared as a procedure result) is in the code. Is C the same as G? Then what is posList? Area of MEM is used multiple times before being introduced. This adds significant confusion.

Response: Thanks for comments for the pseudocode. We have carefully checked and rewritten the pseudocode of Algorithm 1, which has now been moved to the Supplementary Materials for clarity. In this revision, we added the cluster generation process and included the return variable C, representing the resulting clusters, in Algorithm 1. G denotes the compressed de Bruijn graph generated by the BWT-enhanced SplitMEM, and posList is also derived from the de Bruijn graph. Additionally, we have revised the structure of the section describing the area of MEM to improve readability and coherence:

We start by defining MEM and its area in multiple strings, then define MEM with similar fragments and their area, to support the representation of sequences that lack MEMs but share similarity with them. After defining MEM with similar fragments, we proceed to introduce the alignment process within the divide-and-conquer framework, using the sorted MEMs to produce the final alignment results for the sequences corresponding to the clusters.

In definition of area of MEM: why is c first introduced as "center place" of all "sequences in MEM" and then reintroduced as the most frequent value among MEM start positions? The first definition

is strange and vague, but I do not see how it can be the same as more precise second definition. Moreover, is there a reason such a strange value to be used rather than median or truncated average?

Response: Thanks for pointing this. We defined the “center place” **as** the most frequent value among MEM start positions, ensuring that the calculated MEM area is maximized.

The wording in "MEM with similar fragment" definition is very confusing. The word "similar" should not be used in formal definition without proper clarification. Notation "MX" is used without explanation (I assume that this is notation for formal description of "MEM with similar fragment", but when a notion is introduced, nothing should be assumed, everything should be described clearly).

Response: Thanks for pointing this. The word “similar” refers to sequence fragments share similarity with the original MEM sequence, which the similarity strings are founded by SSW library. We have revised the Definitions 3 and 4 to clarify the notation “MX”.

Discussion at the start of "Align by sorting MEMs" section suggests that MEMs can be present only in a subset of input sequences and then alignment of MEM sequence is used to extend MEM to "MEM with similar fragment". It is unclear how the initial set of MEMs is constructed in such a case because MEMs were defined for a given set of input strings and have to occur in each of them.

Response: During the clustering step, the initial set of MEMs is generated by the BWT-enhanced SplitMEM algorithm, which also produces the associated sequence clusters.

Algorithm 2 has the same problems as Algorithm 1: variables that appear out of nowhere.

Response: Thanks for comments for the pseudocode. We have carefully checked and rewritten the pseudocode of Algorithm 2, which has now been moved to the Supplementary Materials for clarity.

It is unclear how the presented algorithm addresses the issues in existing algorthims described in the introduction.

Response: We have revised the following statements to address the issues in existing algorithms discussed in the introduction:

Our method enhances alignment quality and demonstrates superior performance compared to traditional, seed-and-extension-free MSA strategies like MAFFT [14], WMSA [17] and abPOA [33] on low-similarity datasets. Additionally, it supports the alignment of extremely long sequences that these seed-and-extension-free methods cannot handle. Furthermore, deMEM outperforms sequence division methods like FMAlign2 [28] and FAME [26] in handling challenging alignment tasks, such as aligning extremely long sequences.

In results it is unclear why the method is not compared to all MSA tools, listed in the introduction.

Response: Our study focuses on seed-and-extension methods, aiming to improve alignment quality relative to approaches such as FMAlign2 and FAME. Therefore, we did not compare our method to all MSA tools listed in the introduction.

deMEM tool presented in this paper is represented in results as a whole family of tools, when the deMEM pipeline is combined with other alignment tools. This makes it difficult to understand and evaluate the results. Normally in such cases there is a separate comparison between variations of a tool, the best parameters are chosen based on this comparison and in main benchmarking table only one version of the parameters is present. Otherwise the comparison is not fair since competing tools do not have opportunity to iterate through their possible parameter values.
Overall it does not look like deMEM provides consistent advantage in alignment quality, speed or memory consumption. However this may be the result of true advantage hidden behind many different versions of deMEM.

Response: Thanks for pointing this. We have shown the best SP Score for all arguments in different methods. It's worth noting that, different sub methods (MAFFT, abPOA and WMSA) has different alignment scoring systems, we thought the scores are best fit for comparison. For extremely large and long sequences, such as those in the MPoX dataset, although FAME achieved faster alignment with lower memory consumption, it produced lower-quality results compared to deMEM. Specifically, deMEM **improved alignment quality** by approximately 50.3% over FAME and 32.0% over FMAlign2 on the MPoX dataset.

It is unclear why figure 4 has different set of labels compared to the results table.

Response: Figure 4 illustrates the results across multiple datasets, noting that some methods were only applicable to certain cases (e.g., FAME could not be evaluated on the Mycoplasma dataset). The figure labels have been revised to enhance clarity.

List of language problems in the paper:
Page 2: "newly sequenced sequences"
Page 4: "Our method, deMEM, employs the divide-and-conquer strategy achieve efficient and accurate multiple sequence alignment."
Page 4: "Our method enhance quality and demonstrated superior alignment quality compared to MAFFT [14], WMSA [17] and abPOA [33]"
Page 4: "Furthermore, deMEM made better performance"
Page 7: "State-of-the-Art Divide-and-Conquer Algorithm for MSA Using MEMs". Please do not call your method state-of-the-art when you describe it. Let the readers decide it for themselves later when they see benchmarking results.
Page 8: "MEM in multiple strings 1 , 2 , … , is exact matches between multiple strings with match length "
The actual list of language problems is much longer and the general quality deteriorates towards the end of the paper, but I will stop here.

Response: We appreciate the reviewer's comment regarding language issues and have thoroughly

revised the manuscript to improve clarity and readability throughout in response to these suggestions.

Reviewer #3: This paper presents a new framework for multiple sequence alignments, deMEM. It is divided in three steps, which can be quickly summarized by clustering by MEMs using de Brujin graphs, the core functionality of the framework of using divide-and-conquer to align each cluster, and to merge the aligned clusters by some chosen existing method. In the second step, MEMs get relaxed to "MEMs with similar fragments", and the sequences get divided until no more such relaxed MEMs are found.
I find the divide-and-conquer idea very elegant and natural. The framework itself is simple to install and use, and the README is a very helpful resource. However, the paper is unfortunately hard to read, and the evaluation of the results is a bit lacking. Some major issues I listed require more work (like the overall language of the paper), others can be solved rather quickly, but I consider them essential for the understanding of the methods.

Response: Thank you very much for taking the time to comment on our manuscript. Your questions have been addressed point by point below.

Major issues:
- The language is grammatically in a bad shape. Many sentences are very hard to parse.

Response: We appreciate the reviewer's comment regarding language issues and have thoroughly revised the manuscript to improve clarity and readability.

- I think Algorithm 2 needs to be written in a more elementary manner. For example, I do not understand the line "if (interval both in left and right) interval.split_and_append(this_left, this_right);". What do "this_left" and "this_right" refer to? They are undefined. What does the function "split_and_append" do? What is the variable "Block_list"?

Response: Thanks for comments for the pseudocode. We have modified and added the comments to the Algorithm 2, which has now been moved to the Supplementary Materials for clarity. "this_left" and "this_right" means the new intervals (see Algorithm S2 for more details). The function "split_and_append" means split the other MEM following the intervals (also see Algorithm S2 for more details).

- It is mentioned that MEMs with similar fragments are found using the SSW library. However, it is not explained in this paper what "similar fragment" is supposed to mean. Are they substrings with high pairwise identity? If yes, which value is chosen? Definition 3 needs to be more precise.

Response: Thanks for comment. Similar fragment regions are identified using the SSW library based on high pairwise identity, for which we employed the default parameters provided by the library.

- The figure captions often do not refer to the figures in a direct manner. For example, in Figure 1 and Figure 3, it is not shown what the caption is describing by "left subproblem", the "right

subproblem" and the "down subproblem". I have an intuitive idea what these are supposed to mean, but I am not confident in describing them precisely after reading the paper. This should be explained more thoroughly in the main text or shown directly in the Figure.

Response: We have added the left block, right block and down block in Figure 3.

- The paper concludes that deMEM performs better than FAME or FMAlign2 under low conserved sequences. Which datasets do you refer to in particular?

Response: The low conserved sequences we refer to include the *MPoX*, *Srtepococcus pneumonia*, *Escherichia coli* and *Nerisseria meningitidis* datasets.

- What exactly does "perform better" here mean? Can you explain whether there is any trade-off between runtime, SP score and memory usage?

Response: "Perform better" means deMEM outperforms its counterpart tools at large scale data, such as a thousand monkeypox genomes, which improved alignment quality by approximately 50.3% over FAME and 32.0% over FMAlign2 on the MPoX dataset. Our method achieves higher SP scores with only a modest increase in runtime and memory usage.

- "For extremely large and long sequences, such as Mycoplasma bovis sequences, deMEM offers modest improvements in alignment quality but uses significantly less memory": Looking at the table, both the SP measure and the used memory are almost identical among deMEM-FFTNS1-H and FMAlign2 (the difference in SP score is about 2.5, and the difference in memory is about 7MB), but FMAlign2 runs significantly faster. Do I miss anything here?

Response: "Perform better" means deMEM outperforms its counterpart tools at large scale data, such as a thousand monkeypox genomes, which improved alignment quality by approximately 50.3% over FAME and 32.0% over FMAlign2 on the MPoX dataset. We aim to demonstrate deMEM's ability to align extremely long sequences using the *Mycoplasma bovis* dataset, whereas other methods require less memory when aligning other large-scale datasets, such as a thousand MPoX genomes.

Minor issues:
- Algorithm 1 should be self contained. You can then clarify which lines are a direct copy from Baier et al., and which ones are new. In the current version, you need to switch between two different papers to understand your algorithm.

Response: Thanks for comments for the pseudocode. We simply merge these lines to functions (create_BIT_vectors and which_cluster functions) from Baier et al., which we want to emphasize the disjoint-set union for clustering sequences.

- The paper references Figure 3(a), but the figure contains no part (a).

Response: We have removed (a) in Figure 3(a).
