# Supplementary material for: deMEM: a novel divide-and-conquer framework based on de Bruijn graph for scalable multiple sequence alignment
Source: Gigascience. 2026 Jan 5;15:giaf163. doi: 10.1093/gigascience/giaf163 (PMC12878729; doi:10.1093/gigascience/giaf163)
Supplement: giaf163_Authors_Response_To_Reviewer_Comments_Original_Submission [file giaf163_authors_response_to_reviewer_comments_original_submission.pdf]

# Author's Response To Reviewer Comments

Reviewer #1: This paper has significantly improved over the previous submission. I believe that the paper can be accepted, if the authors address a few comments.

Response: Thank you very much for taking the time to comment on our manuscript. Your questions have been addressed point by point below.

Language: I believe that it has improved a lot. There are still several mistakes, but they do not impact readability negatively anymore, in my opinion.

Response: Thank you for your comments. We have carefully reviewed the manuscript and further improved the language throughout to enhance clarity and readability.

Methods -- points not addressed:
- It is still not explained, what a "similar fragment" is in Definition 3. It is only mentioned that they are found using the SSW library, but it is not explained what this library does. What does *similar* mean? There are many ways to compare strings to each other.

Response: The core algorithm of the SSW library is the Smith-Waterman local pairwise sequence alignment. In this context, "similar" refers to the alignment with the highest score between a MEM sequence and the sequence being compared. In the deMEM algorithm, when comparing sequences not included in the MEM to the MEM sequence, we use the Smith-Waterman algorithm to identify the substring with the highest score in the sequence. This substring is considered as the similar fragment. For example, as illustrated in Figure 3, when comparing the sequence "AGAAATGAGCTCCGTATGGCTCGT" with the MEM sequence "GAGCTACGTA", the Smith-Waterman alignment identifies the most similar substring as "GAGCTCCGTA", which is then treated as the similar fragment. We have added this explanation and the parameters of Smith-Waterman algorithm into the manuscript to enhance clarity (line 196 on Page 10).

Methods -- points addressed:
- Algorithm 2 is a little bit easier to understand with the added comments. However, if a pseudo-code can only be understood with extensive comment explanation, it only hardly counts as psuedo-code.

Response: Thanks for pointing this. We agree that the previous version of Algorithm S2 relied too heavily on C++-style syntax and verbose comments, which cluttered the logic. In the revised manuscript, we have completely refactored Algorithm S2 to enhance the readability of pseudo-code. Specifically, we have:
1. Integrated the explanatory logic directly into the flow of the algorithm (e.g., explicitly describing the interval splitting logic in Part 2) rather than hiding it in comments.
2. Used more descriptive function names and abstract variable definitions to improve readability.
We believe the new Algorithm S2 is now self-explanatory and much easier to follow. Please refer to Supplemental Materials for the updated algorithm.

- The figures got improved and now include the "left", "right" and "down" block descriptions.

Response: Thanks for pointing this. We believe that Figure 3 effectively illustrates the core concept of our algorithm.

Results: In this version, I have an easier time understanding the advantages and disadvantages of deMEM. However, the authors introduced a new minor issue that I state below.
New minor issue:
- The authors write "Specifically, deMEM improved alignment quality by approximately 50.3% over FAME and 32.0% over FMAlign2 on the MPoX dataset.". They state it twice, including in the Conclusions. They do not refer to a Figure, though. In the sections, where the Figures are discussed in more detail, this improved alignment quality is not mentioned. How are these percentages computed exactly?

Response: Thanks for your comment. We have clarified the method for calculating the alignment quality improvement in the 'Experimental Metrics' section, under 'Datasets and Measurement'. Specifically, we define the quality improvement as the SP score enhancement, which is detailed in Formula (4). The corresponding SP scores can be found in Supplementary Table S1.

---


Editorial Manager®

## Cookie Preference Center

We use cookies which are necessary to make our site work. We may also use additional cookies to analyze, improve and personalize our content and your digital experience. For more information, see our Cookie Policy.

You may choose not to allow some types of cookies. However, blocking some types may impact your experience of our site and the services we are able to offer. See the different category headings below to find out more or change your settings.

Allow all

## Manage Consent Preferences

### Strictly Necessary Cookies

**Always active**

These cookies are necessary for the website to function and cannot be switched off in our systems. They are usually only set in response to actions made by you which amount to a request for services, such as setting your privacy preferences, logging in or filling in forms. You can set your browser to block or alert you about these cookies, but some parts of the site will not then work.

### Functional Cookies

These cookies enable the website to provide enhanced functionality and personalisation. They may be set by us or by third party providers whose services we

have added to our pages. If you do not allow these cookies then some or all of these services may not function properly.

**Performance Cookies**

These cookies allow us to count visits and traffic sources so we can measure and improve the performance of our site. They help us to know which pages are the most and least popular and see how visitors move around the site.

**Targeting Cookies**

These cookies may be set through our site by our advertising partners. They may be used by those companies to build a profile of your interests and show you relevant adverts on other sites. If you do not allow these cookies, you will experience less targeted advertising.

## Cookie List

Clear

Apply Cancel
Consent Leg.Interest

Confirm my choices

Powered by onetrust
