# Supplementary material for: deMEM: a novel divide-and-conquer framework based on de Bruijn graph for scalable multiple sequence alignment
Source: Gigascience. 2026 Jan 5;15:giaf163. doi: 10.1093/gigascience/giaf163 (PMC12878729; doi:10.1093/gigascience/giaf163)
Supplement: giaf163_Reviewer_1_Report_Original_Submission — Andreas Grigorjew -- 11/26/2025 [file giaf163_reviewer_1_report_original_submission.pdf]

Reviewer Report

Title: deMEM: a novel divide-and-conquer framework based on de Bruijn graph for scalable multiple sequence alignment

Version: Original Submission **Date:** 11/26/2025

Reviewer name: Andreas Grigorjew

Reviewer Comments to Author:

This paper has significantly improved over the previous submission. I believe that the paper can be accepted, if the authors address a few comments.Language: I believe that it has improved a lot. There are still several mistakes, but they do not impact readability negatively anymore, in my opinion.Methods -- points not addressed:- It is still not explained, what a "similar fragment" is in Definition 3. It is only mentioned that they are found using the SSW library, but it is not explained what this library does. What does *similar* mean? There are many ways to compare strings to each other.Methods -- points addressed:- Algorithm 2 is a little bit easier to understand with the added comments. However, if a pseudo-code can only be understood with extensive comment explanation, it only hardly counts as psuedo-code.- The figures got improved and now include the "left", "right" and "down" block descriptions.Results: In this version, I have an easier time understanding the advantages and disadvantages of deMEM. However, the authors introduced a new minor issue that I state below.New minor issue:- The authors write "Specifically, deMEM improved alignment quality by approximately 50.3% over FAME and 32.0% over FMAlign2 on the MPoX dataset.". They state it twice, including in the Conclusions. They do not refer to a Figure, though. In the sections, where the Figures are discussed in more detail, this improved alignment quality is not mentioned. How are these percentages computed exactly?

Level of Interest

Please indicate how interesting you found the manuscript: Choose an item.

Quality of Written English

Please indicate the quality of language in the manuscript: Choose an item.

Declaration of Competing Interests

Please complete a declaration of competing interests, considering the following questions:

- Have you in the past five years received reimbursements, fees, funding, or salary from an organisation that may in any way gain or lose financially from the publication of this manuscript, either now or in the future?

- Do you hold any stocks or shares in an organisation that may in any way gain or lose financially from the publication of this manuscript, either now or in the future?
- Do you hold or are you currently applying for any patents relating to the content of the manuscript?
- Have you received reimbursements, fees, funding, or salary from an organization that holds or has applied for patents relating to the content of the manuscript?
- Do you have any other financial competing interests?
- Do you have any non-financial competing interests in relation to this paper?

If you can answer no to all of the above, write 'I declare that I have no competing interests' below. If your reply is yes to any, please give details below.

1. no 2. no 3. no 4. no 5. no 6. no

I agree to the open peer review policy of the journal. I understand that my name will be included on my report to the authors and, if the manuscript is accepted for publication, my named report including any attachments I upload will be posted on the website along with the authors' responses. I agree for my report to be made available under an Open Access Creative Commons CC-BY license (http://creativecommons.org/licenses/by/4.0/). I understand that any comments which I do not wish to be included in my named report can be included as confidential comments to the editors, which will not be published.

Choose an item.

To further support our reviewers, we have joined with Publons, where you can gain additional credit to further highlight your hard work (see: https://publons.com/journal/530/gigascience). On publication of this paper, your review will be automatically added to Publons, you can then choose whether or not to claim your Publons credit. I understand this statement.

Yes Choose an item.
